# Screening of environmental fungi from Crete reveals candidates for biological control of mosquitoes

**DOI:** 10.1093/jme/tjag069

**Published:** 2026-05-26

**Authors:** Joel C Couceiro, Martyn J Wood, Juan J Silva, Andronikos Papadopoulos, John Vontas, George Dimopoulos

**Affiliations:** Institute of Molecular Biology and Biotechnology, Foundation for Research and Technology Hellas (IMBB-FORTH), Heraklion, Crete, Greece; Institute of Molecular Biology and Biotechnology, Foundation for Research and Technology Hellas (IMBB-FORTH), Heraklion, Crete, Greece; Institute of Molecular Biology and Biotechnology, Foundation for Research and Technology Hellas (IMBB-FORTH), Heraklion, Crete, Greece; Institute of Molecular Biology and Biotechnology, Foundation for Research and Technology Hellas (IMBB-FORTH), Heraklion, Crete, Greece; Institute of Molecular Biology and Biotechnology, Foundation for Research and Technology Hellas (IMBB-FORTH), Heraklion, Crete, Greece; Pesticide Science Laboratory, Department of Crop Science, Agricultural University of Athens, Athens, Greece; Institute of Molecular Biology and Biotechnology, Foundation for Research and Technology Hellas (IMBB-FORTH), Heraklion, Crete, Greece; W. Harry Feinstone Department of Molecular Microbiology and Immunology, Bloomberg School of Public Health, Johns Hopkins University, Baltimore, MD, USA

**Keywords:** entomopathogen, larvicide, adulticide, mosquito control

## Abstract

Mosquito-borne diseases remain a pressing global health concern, exacerbated by vector range-expansion and insecticide resistance. Entomopathogenic fungi represent a promising alternative to chemical insecticides, yet most mycoinsecticides are based on a few well-studied species. In this study, we surveyed environmental samples from Crete, Greece, isolating 46 isolates representing 13 genera. After a preliminary screening, 8 isolates were selected for larvicidal and adulticidal assays against *Culex pipiens molestus* Forskål (Diptera: Culicidae). The most promising isolate was BS2-C3 (*Metarhizium* Sorokin) which achieved over 90% mortality in both assays. Isolates from other genera (eg *Cladosporium* Link, *Penicillium* Link, *Purpureocillium* Luangsa-ard, Hywel-Jones, Houbraken & Samson) also exhibited activity, warranting further investigation. The performance of BS2-C3 was compared with commercially available strains GHA [*Beauveria bassiana* (Bals.-Criv.) Vuill.] and V275 (*Metarhizium brunneum* Petch), being as efficient as V275 and more efficient than GHA. These findings demonstrate that, in addition to the well-established genera, other fungal taxa may also harbor mosquitocidal potential, underscoring the importance of continued exploration of fungal diversity for biocontrol. Overall, our results expand the pool of fungi with demonstrated mosquito activity and highlight BS2-C3 (*Metarhizium* sp.) as a strong candidate for further development into safe, effective, and environmentally sustainable mycoinsecticides for integrated vector management.

## Graphical Abstract

**Graphical Abstract:**
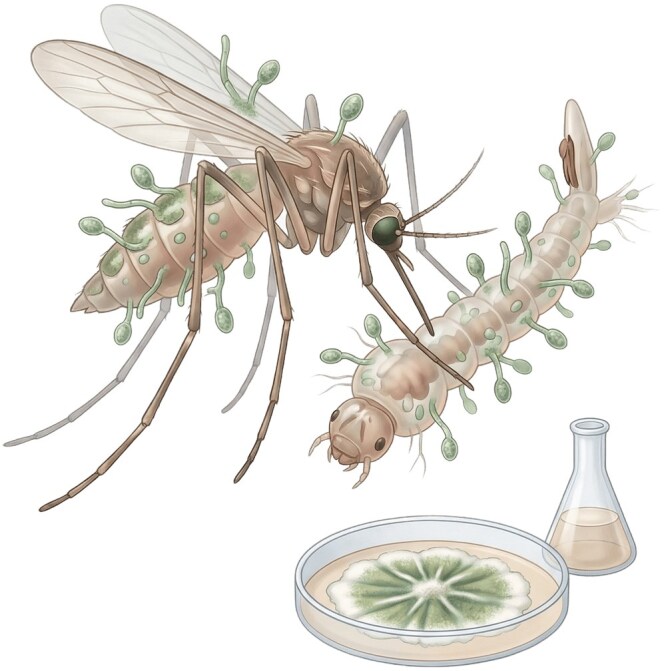

## Introduction

Graphical Abstract

Entomopathogenic fungi (EPF) are widely used as biological control agents against agricultural and urban pests, demonstrating great potential for incorporation into integrated pest management (IPM) strategies (Lacey et al. 2015, Sharma et al. 2023). These fungi infect insect hosts through the cuticle and exhibit generally low toxicity to nontarget organisms, making them safe alternatives for sustainable pest control (Zimmermann 2007a, 2007b, Sharma et al. 2023). In agricultural systems, several EPF-based products have been developed and commercialized as bioinsecticides (Faria and Wraight 2007, Couceiro et al. 2025), demonstrating their practical applicability under field conditions. Their established role in IPM has also driven interest in their use for vector control, where similar principles of efficacy, safety, and environmental compatibility are applied.

In mosquito control, EPF have been primarily investigated using species of *Beauveria* Vuill. and *Metarhizium* Sorokin, which have demonstrated high efficacy against a range of mosquito species, including *Aedes* Meigen, *Anopheles* Meigen, and *Culex* L. (Bukhari et al. 2011, Valero-Jiménez et al. 2014, Greenfield et al. 2015, Lee et al. 2019, Vivekanandhan et al. 2020, Bitencourt et al. 2021, Hamama et al. 2022). Efforts to enhance their performance have focused on improving formulation and delivery systems, as well as genetic modification to increase virulence and environmental persistence (Wang and St Leger 2007, Morales Hernandez et al. 2010, Fang et al. 2011, Peng and Xia 2015, Yaakov et al. 2018, Shin et al. 2023).

Despite these advances, the efficacy of fungal agents in mosquito control remains highly variable and strain-dependent, limiting their consistent application. Although several studies have demonstrated high efficacy of selected *Beauveria* and *Metarhizium* isolates, these reports typically focus on a small number of isolates, often evaluated under different experimental conditions, making comparisons difficult (Qin et al. 2023, Perumal et al. 2024), while research performing broader screening of diverse fungal taxa remains comparatively underexplored (Accoti et al. 2021). Expanding screening efforts to include geographically and ecologically diverse environments, while applying consistent evaluation criteria, is necessary to improve the identification of candidate fungal strains for mosquito control.

The present study was designed as a targeted screening effort to address these limitations by identifying fungal isolates with potential for mosquito control. Environmental samples were collected from diverse habitats on the island of Crete, Greece, a Mediterranean region characterized by heterogeneous ecosystems that may harbor underexplored fungal diversity. Isolates were evaluated against both larval and adult stages of *Culex pipiens molestus* Forskål (Diptera: Culicidae), an important vector of arboviruses. Isolate selection was based on a combination of insecticidal efficacy and conidial production, a key trait for subsequent mass production, formulation, and development of mycoinsecticides (Jackson et al. 2010). By integrating ecological sampling with application-oriented screening criteria, and comparing selected isolates with reference strains, this study aimed to expand the pool of candidate fungi and identify isolates with potential for further development as mosquito control agents.

## Materials and Methods

### Sample Collection, Processing, and Establishment of Fungal Library

Seventeen locations across Crete were selected to represent a broad range of environmental conditions relevant to the occurrence of EPF. Site selection was based on habitat type (riverside, forest, grassland, and agricultural areas), vegetation composition (olive groves, grass patches, shrubs, pine and oak trees, herbaceous plants), soil characteristics (moisture, organic matter content), and presence of insects. Environmental samples were collected using a semi-targeted approach, focusing on rhizosphere soil, plant material (roots, stems, and leaves), and dead insects to increase the likelihood of isolating diverse fungal taxa (Meyling and Eilenberg 2007; Hesketh et al. 2010).

A total of 36 samples were collected: 13 from insects, 9 from soil, 6 from plant roots, 5 from leaves, 2 from stems, and 1 from water. Dead insects (Diptera and Hemiptera) and soil samples were collected in 1.5-ml tubes. Plant material was collected in 15-ml tubes: For grasses, the leaf, stem, and root were carefully removed together, while for larger plants or trees, individual leaves and, when possible, bark were collected using a forceps. A biofilm sample was collected from the surface layer of a small freshwater pond in a 15-ml tube. In 3 locations representing distinct habitat types (riverside, forest, and grassland), bulk soil samples (∼4 kg) were collected in single plastic containers (38 × 28 × 8.5 cm). Geographic coordinates for all sites are available in Supplementary Material S1.

All samples were transported to the laboratory for immediate culture. Insects were surface-sterilized by sequential immersion in 70% alcohol, 1% sodium hypochlorite, and sterile distilled water (30 s each), then plated on Sabouraud Dextrose Agar (SDA). Plant material was surface-sterilized as above, cut into fragments (∼2 cm) and inoculated on SDA. Soil and a biofilm sample were mixed with 1 ml of 1X PBS, vortexed, and 100-μl aliquots were plated on SDA. Each sample was cultured in triplicate, incubated at 27 ± 1 °C for 7 to 14 d; distinct colonies were isolated and cultured under the same conditions.

Fungi present in the bulk soil were isolated employing the insect-baiting technique (Vega et al. 2012) using larvae of *Tenebrio molitor* L. (Coleoptera: Tenebrionidae), a model insect for this method due to its susceptibility to a wide range of EPF and ease of rearing (Bharadwaj and Stafford 2011). Approximately 80 third- to fourth-instar larvae of *T. molitor* were released in each container and allowed to move freely in the soil. Every 2 d, dead larvae were collected, surface-sterilized and placed in moist chambers at 27 ± 1 °C for 7 d. Fungi that emerged from the cadavers were isolated on SDA as above. All isolates were cryopreserved and deposited in the MicroBioPest Microbial Collection (IMBB-FORTH, Heraklion, Crete, Greece) in 20% glycerol at −80 °C.

### Insect Rearing

An autogenous colony of *C. pipiens molestus* was reared in a climate-controlled incubator (HC2A-S-Memmert, Memmert, Germany) at 27 ± 1 °C, 70 ± 5% R.H., 16:8 L:D photoperiod. Larvae were reared in plastic containers (38 × 28 × 8.5 cm) with tap water and fed with flaked fish food (sera Vipan Nature, sera, Heinsberg, Germany) until pupation. Pupae were transferred to 500-ml cups and placed inside BugDorm-1 Insect Cages (30 × 30 × 30 cm; MegaView Science Co., Ltd., Taichung, Taiwan). Adults were fed with a 10% sugar solution, and a water cup was provided for oviposition.

### Preliminary Screening

All isolates were preliminarily screened to identify candidates suitable for further development, based on 3 complementary criteria: ease of culture, conidial production (asexual infective spores), and rapid mortality to target insects. For culturing assessment, each isolate was cultured for 7 d, at 27 ± 1 °C, on SDA, PDA (potato dextrose agar), and SDAY/4 (one-quarter strength Sabouraud dextrose agar supplemented with yeast extract). Based on morphological observations of the culture plates after the growth period, SDA or SDAY/4 supported best growth and were used for subsequent culturing (Table 1).

**Table 1.** List of isolates obtained in the current study

Isolate code	Source	Taxonomic classification	Accession number (GenBank)	Optimal culture medium for growth
**OTN24-CD4**	Leaf endophyte	*Cladosporium* sp.	PX136791	SDA
**OTN58-CD1**	Stagnant water	*Cladosporium* sp.	PX136792	SDA
**OTN75-CD1**	Root endophyte	*Cladosporium* sp.	PX136793	SDA
**OTN83-CD5**	Leaf endophyte	*Aureobasidium* sp.	PX136794	SDA or SDAY/4
**OTN99-C4**	Olive tree bark	*Aureobasidium* sp.	PX136795	SDA or SDAY/4
**ONT100-C1**	Dead insect	*Cladosporium* sp.	PX136796	SDA
**OTN102-C2**	Dead insect	*Cladosporium* sp.	PX136797	SDA
**OTN103-C1**	Dead insect	*Cladosporium* sp.	PX136798	SDA
**OTN108-CD3**	Soil	*Talaromyces* sp.	PX136799	SDAY/4
**OTN122-CD1**	Root endophyte	*Mortierella* sp.	PX136800	SDA
**OTN123-C3**	Soil	*Hyphopichia* sp.	PX136801	SDA or SDAY/4
**OTN123-C4**	Soil	Cordycipitaceae	PX136802	SDA
**OTN123-CD4**	Soil	*Rhodotorula* sp.	PX136803	SDA or SDAY/4
**OTN128-C1**	Stem endophyte	*Fusarium* sp.	PX136804	SDA
**OTN128-CD4**	Stem endophyte	*Cladosporium* sp.	PX136805	SDA
**OTN129-CD5**	Soil	*Penicillium* sp.	PX136806	SDA
**OTN137-C5**	Rhizosphere soil	*Cladosporium* sp.	PX136807	SDA
**OTN139-C2**	Stem endophyte	*Cladosporium* sp.	PX136808	SDA
**OTN145-C3**	Soil	*Mortierella* sp.	PX136809	SDAY/4
**OTN145-CD5**	Soil	*Penicillium* sp.	PX136810	SDA
**OTN148-C1**	Dead insect	*Cladosporium* sp.	PX136811	SDA
**OTN149-C4**	Dead insect	*Cladosporium* sp.	PX136812	SDA
**OTN150-C1**	Dead insect	*Cladosporium* sp.	PX136813	SDA
**OTN151-C1**	Dead insect	*Cladosporium* sp.	PX136814	SDA
**OTN153-C12**	Rhizosphere soil	*Fusarium* sp.	PX136815	SDA
**OTN154-C7**	Stem endophyte	*Fusarium* sp.	PX136816	SDA
**OTN154-C11**	Stem endophyte	*Fusarium* sp.	PX136817	SDA
**OTN158-C1**	Dead insect	*Cladosporium* sp.	PX136818	SDA
**OTN159-C1**	Dead insect	*Cladosporium* sp.	PX136819	SDA
**OTN161-C2**	Leaf endophyte	*Epicoccum* sp.	PX136820	SDA
**OTN163-CD1**	Leaf endophyte	*Penicillium* sp.	PX136821	SDA
**OTN172-C1**	Dead insect	*Cladosporium* sp.	PX136822	SDA
**OTN173-C2**	Dead insect	*Cladosporium* sp.	PX136823	SDA
**OTN177-C2**	Dead insect	*Cladosporium* sp.	PX136824	SDA
**OTN178-CD2**	Root endophyte	*Penicillium* sp.	PX136825	SDA
**OTN178-CD3**	Root endophyte	*Aspergillus* sp.	PX136826	SDA
**OTN179-CD3**	Stem endophyte	*Cladosporium* sp.	PX136827	SDA
**OTN181-CD1**	Soil	*Purpureocillium* sp.	PX136828	SDA
**OTN182-C1**	Dead insect	*Alternaria* sp.	PX136829	SDA
**OTN185-C6**	Leaf endophyte	*Aspergillus* sp.	PX136830	SDA
**OTN185-CD5**	Leaf endophyte	*Cladosporium* sp.	PX136831	SDA
**BS1-C3**	Soil	*Hyphopichia* sp.	PX136832	SDA
**BS1-C4**	Soil	*Penicillium* sp.	PX136833	SDA or SDAY/4
**BS1-C6**	Soil	*Cladosporium* sp.	PX136834	SDA
**BS2-C3**	Soil	*Metarhizium* sp.	PX136835	SDAY/4
**BS2-C4**	Soil	*Fusarium* sp.	PX136836	SDA or SDAY/4

Taxonomic classification is based on sequencing of the ITS region of the rRNA, after BLAST-ing query sequences against the UNITE database. All isolates are deposited in the MicroBioPest Microbial Collection, IMBB-FORTH, Heraklion, Crete, Greece.

To assess insecticidal activity, fungal cultures were grown for 7 d at 27 ± 1 °C. Conidial suspensions were prepared by washing 2 plates per isolate with a 0.05% Tween 80 solution. At this stage, concentrations of the suspensions were not determined, as the objective was to rapidly identify promising isolates based on overall performance, including growth and sporulation capacity. One hundred microliters of each suspension were added to 900 μl of distilled water in 48-well plates, with 4 *C. pipiens molestus* larvae per well (third- and fourth-instar) and 2 wells per isolate (*n* = 8 per isolate). Larvae were fed a small amount of pulverized fish food using a micro spoon spatula, and mortality was recorded daily for 10 d. Isolates that caused 100% larval mortality within 6 d were selected for further screening. For the controls, 100 μl of a 0.05% Tween 80 solution was added to the wells.

The selected isolates were subsequently assessed for quantitative conidial production. Fungal suspensions were prepared as above but using a single culture plate; only isolates yielding at least 10^7^ conidia/ml, as determined using a Neubauer hemocytometer, were selected for efficacy assays. This screening approach allowed rapid identification of promising isolates while accounting for growth, sporulation, and efficacy, and ensured that only isolates meeting the defined thresholds were advanced. Due to the exploratory nature and limited number of larvae and replicates at this stage, data were not subjected to statistical analysis.

### Fungal Identification

#### DNA Extraction and Sequencing

Yeast and yeast-like isolates were identified based on colony morphology and confirmed by microscopic observation. For these isolates, culturing and DNA extraction followed the protocols described in Dymond (2013) with minor modifications. Briefly, cultures were grown overnight in YPD at 30 °C and 200 rpm. Cell pellets were harvested by centrifugation (3,600 rpm for 5 min), resuspended in 1 ml H_2_O and centrifuged again under the same conditions. Supernatants were discarded, then the pellets were resuspended in 190 μl of extraction buffer (0.1 M Tris HCl, 0.05 M EDTA, 0.5 M NaCl) and 20 μl of 20% SDS solution (modification 1), and disrupted by adding glass beads, 400 μl of phenol/chloroform/isoamyl alcohol (25:24:1) and vortexing for 2 min. After phase separation, DNA was precipitated with cold ethanol, washed with 70% ethanol, dried at room temperature for ∼5 min, then resuspended in 500 μl of TE buffer. The samples were treated with 3 μl of RNAse A (10 mg/ml) (modification 2) at 37 °C for 30 min, followed by additional phenol/chloroform/isoamyl alcohol (25:24:1) extraction and ethanol precipitation, including the addition of 5M potassium acetate (modification 3). The resulting pellet was washed with 70% ethanol, dried at room temperature for ∼5 min, and resuspended in 100 μl of TE buffer.

For non-yeast fungi, DNA extraction followed the protocol from Couceiro et al. (2022). Briefly, isolates were grown in YPD broth for 3 d under agitation. Then, fungal material was harvested, lyophilized, and ground to a fine powder. Cells were lysed using 500 μl of CTAB buffer supplemented with 1 μl of 2-mercaptoethanol, 3 μl of RNase A (10 mg/ml), and 3 μl of proteinase K, followed by sequential extractions using 500 μl of phenol/chloroform/isoamyl alcohol (25:24:1) and 500 μl of chloroform/isoamyl alcohol (24:1). DNA was precipitated with isopropanol, washed with ethanol, air-dried, and resuspended in 50 μl of TE buffer.

When the quality of the extraction was low, an alternative protocol was used. Pellets of dried fungal material were put in 1.5-ml tubes and crushed into a powder. To each sample, it was added 200 μl of extraction buffer, 35 μl of 20% SDS solution, 3 μl of RNAse A (10 mg/ml), and 3 μl of proteinase K (10 mg/ml), then the tubes were placed in a heat block at 60 °C for 30 min. After that, 160 μl of 5 M potassium acetate were added, followed by centrifugation at 19,722 *g* and 4 °C for 10 min. The supernatants were transferred to new tubes containing 500 μl of cold isopropanol and stored at −20 °C overnight. The following day, the samples were centrifuged at 19,722 *g* and 4 °C for 10 min. The supernatants were discarded, and 500 μl of cold 70% ethanol were added to each sample, followed by centrifugation at 19,722 *g* and 4 °C for 10 min. The 70% ethanol was discarded, and the DNA was rinsed 3 times with 70% ethanol. The tubes were left to air-dry, then the DNA was dissolved in 50 μl of 1X TE buffer (pre-heated in a heat block at 60 °C). The quality of the extractions was assessed on a 0.8% agarose gel and on NanoDrop ND-1000 Spectrophotometer (Thermo Fisher Scientific), in which case the quality was evaluated based on 260/280 and 260/230 absorbance ratios, and samples with acceptable purity were used for downstream applications.

Polymerase chain reactions (PCR) were conducted for the ITS (internal transcribed spacer) region of the rRNA, using the primers ITS1F (5′-CTTGGTCATTTAGAGGAAGTAA) (Gardes and Bruns 1993) and ITS4 (5′-TCCTCCGCTTATTGATATGC) (White et al. 1990), and PCR conditions were: 95 °C for 5 min; 35 cycles of 94 °C for 30 s, 52 °C for 30 s and 72 °C for 1 min; and 72 °C for 8 min. Amplification success was verified on 1% agarose gels. Samples were purified using NucleoSpin Gel and PCR Clean-up kit (Macherey-Nagel), according to the manufacturer’s instructions, and sequenced by GENEWIZ, from Azenta Life Sciences (Leipzig, Germany).

#### Sequencing Analysis

BioEdit Sequence Alignment Editor version 7.7.1 (https://thalljiscience.github.io/) was used to analyze the chromatograms and edit the sequences. They were deposited in GenBank, and their accession numbers are found in Table 1. Searches using the BLAST algorithm (Basic Local Alignment Search Tool) were performed against UNITE (Abarenkov et al. 2024) using the tool massBLASTer (BLAST+ 2.13.0), with the parameters UNITE (fungi), INSD (fungi) and environmental, in the web-based workbench PlutoF (Abarenkov et al. 2010). Reference sequences were retrieved (GenBank and UNITE accession numbers presented in the Supplementary Material S2), and multiple sequence alignments were performed for each isolate, using MUSCLE v3.8.1551 (Edgar 2004). trimAl 1.2rev59 (Capella-Gutiérrez et al. 2009) was used to trim the alignments (-automated1). Maximum-likelihood trees were constructed using IQ-TREE version 3.0.1 (Wong et al. 2025), applying ModelFinder (-m MFP) (Kalyaanamoorthy et al. 2017) for substitution model selection, and ultrafast bootstrap (Hoang et al. 2018) with 1,000 bootstrap replicates for branch support. Phylogenetic trees were edited using FigTree version 1.4.4 (https://github.com/rambaut/figtree/releases).

### Efficacy Bioassays

#### Fungal Cultivation and Preparation of Suspensions

Isolates were cultured on either SDA or SDAY/4, depending on which medium supported optimal growth, as determined in the preliminary screening, for 7 to 10 d at 27 ± 1 °C, after which conidia were harvested, suspended in a 0.05% Tween 80 solution, and counted using a Neubauer hemocytometer. Suspension concentrations were adjusted to 10^8^ conidia/ml.

#### Larvicidal Assays

Second- and third-instar *C. pipiens molestus* larvae were placed in plastic cups (polypropylene; 180 ml) containing 99 ml of distilled water, to which 1 ml of conidial suspension was added, resulting in a concentration of 10^6^ conidia/ml per cup. Controls received 1 ml of 0.05% Tween 80 solution. Larvae were fed flaked fish food and maintained at 27 ± 1 °C. Larval mortality was evaluated daily for 7 d. Each isolate was tested in triplicate, with 10 larvae per cup (*n* = 30 per isolate), and the whole experiment was repeated 3 times (*n* = 90 per isolate).

#### Adulticidal Assays

Using an air-brush (MAB-34K, Maestro Industrial Equipment, Athens, Greece), 2 ml of the suspensions (10^8^ conidia/ml) were applied to plastic cups (polypropylene; 300 ml). For the controls, 2 ml of 0.05% Tween 80 solution were applied. Cups were left to dry at room temperature under a class II biosafety cabinet, then 10 adult mosquitoes (5 males and 5 females; 5 to 7 d old) were transferred to each cup, with a wet cotton immersed in a 10% sugar solution placed on the lids for feeding. To help avoid loss of humidity inside the cups, a second lid was placed over the cotton. Cups were kept at 27 ± 1 °C, and adult mortality was evaluated daily for 10 d. Each isolate was tested in triplicate (*n* = 30 per isolate), and the whole experiment was repeated 3 times (*n* = 90 per isolate).

The first adulticidal assay included the 8 isolates selected from the preliminary screening. Based on the results, a second adulticidal assay was conducted, comparing isolate BS2-C3 (*Metarhizium* sp.) with 2 commercial EPF isolates, GHA [*Beauveria bassiana* (Bals.-Criv.) Vuill.; Botanigard] and V275 (*Metarhizium brunneum* Petch; Lalguard M52 OD, formerly Met52). Both assays were conducted using the methods described above.

Dead adults were collected, surface-sterilized (described in Section 2.1), and incubated in moist chambers at 27 ± 1 °C for 7 d to allow sporulation. Emerging fungi were cultured on SDA to confirm that they were the applied fungi and not contaminants.

### Statistical Analyses

All analyses were performed using R version 4.4.3 (R Core Team 2025) and assumed a significance threshold of α = 5%. Mortality data were analyzed using Kaplan–Meier survival curves, using the package “survival” version 3.8-3 (Therneau and Grambsch 2000, Therneau 2024). The package “survminer” version 0.5.0 (Kassambara et al. 2024) was used to plot the survival curves and to calculate pairwise comparisons via log-rank tests. Lethal times estimates (LT_50_ and LT_90_) were estimated using parametric Weibull survival models fitted using the package “flexsurv” version 2.3.2 (Jackson 2016). LT_50_ values are presented as descriptive estimates with 95% confidence intervals (CI) and were not subjected to additional statistical comparison, as differences among treatments were evaluated using the log-rank tests.

Sporulation data (proportion of sporulated cadavers relative to the total number of cadavers per treatment) were analyzed using either generalized linear models (GLMs) or generalized linear mixed models (GLMMs), depending on the dataset. Both GLM and GLMM assumed a binomial distribution with a logit link function and were fitted using the package “lme4” version 1.1.36 (Bates et al. 2015). Model selection was based on Akaike’s information criterion, and model fit was evaluated using residual diagnostics from the package “DHARMa” (Hartig 2024). Controls were excluded from the models due to complete separation and were compared separately to each fungal treatment using Fisher’s exact tests with false discovery rate (FDR) adjustment. Post-hoc pairwise comparisons among treatments were performed using the package “emmeans” version 1.11.0 (Lenth 2025).

## Results

### Fungal Sample Collection and Molecular Identification

Internal transcribed spacer-based molecular identification revealed 13 genera. *Cladosporium* Link was the most abundant (21 isolates), followed by *Fusarium* Link and *Penicillium* Link (5 isolates each). *Aspergillus* P. Micheli ex Haller, *Aureobasidium* Viala & G. Boyer, *Hyphopichia* Arx & Van der Walt, and *Mortierella* Coem. were represented by 2 isolates each, while *Alternaria* Nees, *Epicoccum* Link, *Metarhizium*, *Purpureocillium* Luangsa-ard, Hywel-Jones, Houbraken & Samson, *Rhodotorula* F.C. Harrison and *Talaromyces* C.R. Benj. were each represented by a single isolate. One isolate (OTN123-C4) was identified only to family level (Cordycipitaceae) due to insufficient ITS resolution.

Details of each isolate, including origin, taxonomic assignment, and GenBank accession number, are listed in Table 1, whereas phylogenetic trees are available in the Supplementary Material S3.

### Preliminary Screening

The preliminary screening to identify mosquito-pathogenic isolates was exploratory, performed with limited replicates; therefore, no statistical analysis was conducted. Isolates were ranked by LT_100_ (time to 100% mortality), and descriptive values are presented in Table 2. Among the 46 isolates tested, 22 caused 100% larval mortality, with 12 achieving it within 6 d.

**Table 2.** Preliminary screening results for all isolates

Isolate code	LT_100_ (days)	Conidial production	Isolate code	LT_100_ (days)	Conidial production
Control	**–**	**–**	OTN151-C1 (*Cladosporium* sp.)	–	–
OTN24-CD4 (*Cladosporium* sp.)	–	–	OTN153-C12 (*Fusarium* sp.)	9	–
OTN58-CD1 (*Cladosporium* sp.)	–	–	OTN154-C7 (*Fusarium* sp.)	–	–
OTN75-CD1 (*Cladosporium* sp.)	8	–	OTN154-C11 (*Fusarium* sp.)	–	–
OTN83-CD5 (*Aureobasidium* sp.)	6	<10^7^	OTN158-C1 (*Cladosporium* sp.)	10	–
OTN99-C4 (*Aureobasidium* sp.)	5	<10^7^	**OTN159-C1 ** **(*Cladosporium* sp.)**	6	>10^7^
**ONT100-C1 (*Cladosporium* sp.)**	6	**>**10^7^	OTN161-C2 (*Epicoccum* sp.)	–	–
OTN102-C2 (*Cladosporium* sp.)	–	–	OTN163-CD1 (*Penicillium* sp.)	–	–
**OTN103-C1 (*Cladosporium* sp.)**	6	>10^7^	OTN172-C1 (*Cladosporium* sp.)	–	–
**OTN108-CD3 (*Talaromyces* sp.)**	6	>10^7^	OTN173-C2 (*Cladosporium* sp.)	–	–
OTN122-CD1 (*Mortierella* sp.)	–	–	OTN177-C2 (*Cladosporium* sp.)	–	–
OTN123-C3 (*Hyphopichia* sp.)	6	<10^7^	OTN178-CD2 (*Penicillium* sp.)	–	–
OTN123-C4 (Cordycipitaceae)	–	–	OTN178-CD3 (*Aspergillus* sp.)	7	–
OTN123-CD4 (*Rhodotorula* sp.)	7	–	OTN179-CD3 (*Cladosporium* sp.)	9	–
OTN128-C1 (*Fusarium* sp.)	–	–	**OTN181-CD1 ** **(*Purpureocillium* sp.)**	6	>10^7^
OTN128-CD4 (*Cladosporium* sp.)	–	**–**	OTN182-C1 (*Alternaria* sp.)	–	–
OTN129-CD5 (*Penicillium* sp.)	–	–	OTN185-C6 (*Aspergillus* sp.)	–	–
OTN137-C5 (*Cladosporium* sp.)	8	–	OTN185-CD5 (*Cladosporium* sp.)	–	–
OTN139-C2 (*Cladosporium* sp.)	–	–	BS1-C3 (*Hyphopichia* sp.)	7	–
OTN145-C3 (*Mortierella* sp.)	4	<10^7^	**BS1-C4** (***Penicillium* sp.)**	6	>10^7^
OTN145-CD5 (*Penicillium* sp.)	–	**–**	**BS1-C6 ** **(*Cladosporium* sp.)**	6	>10^7^
OTN148-C1 (*Cladosporium* sp.)	–	**–**	**BS2-C3 ** **(*Metarhizium* sp.)**	4	>10^7^
OTN149-C4 (*Cladosporium* sp.)	–	**–**	BS2-C4 (*Fusarium* sp.)	8	–
OTN150-C1 (*Cladosporium* sp.)	9	–			

LT_100_ (time to 100% larval mortality; in days) was recorded for all isolates by exposing *Culex pipiens molestus* larvae to conidial suspensions. Control was exposed to a 0.05% Tween 80 solution. Isolates with LT_100_ ≤ 6 d were further evaluated for conidial production, measured from a single culture plate and reported as higher (>) or lower (<) than 10^7^ conidia/ml. Isolates highlighted in bold met both selection criteria (LT_100_ ≤ 6 d and conidial production >10^7^ conidia/ml) and were selected for subsequent efficacy assays.

Production of high yields of infective propagules is an integral aspect of isolate selection during screening and subsequent development of mycoinsecticides; therefore, we assessed the sporulation of these 12 isolates. Eight candidates produced over 10^7^ conidia/ml from a single culture plate: OTN100-C1, OTN103-C1, OTN159-C1, BS1-C6 (*Cladosporium* sp.), OTN108-CD3 (*Talaromyces* sp.), OTN181-CD1 (*Purpureocillium* sp.), BS1-C4 (*Penicillium* sp.), and BS2-C3 (*Metarhizium* sp.). These isolates were selected for further assays based on their combined performance in mortality and conidial production.

### Efficacy Bioassays

#### Larvicidal Activity

**Fig. 1.**
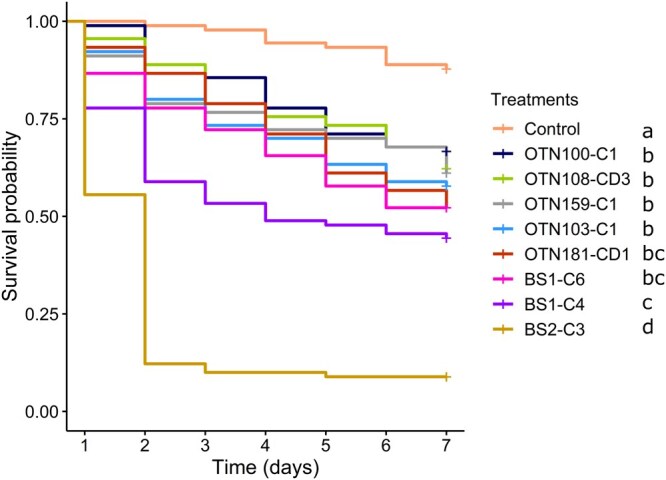
Daily cumulative survival probability (Kaplan–Meier) of *Culex pipiens molestus* larvae exposed to conidial suspensions (10^6^ conidia/ml) of different fungi (*Cladosporium*: OTN100-C1, OTN103-C1, OTN159-C1 and BS1-C6; *Talaromyces*: OTN108-CD3; *Purpureocillium*: OTN181-CD1; *Penicillium*: BS1-C4; *Metarhizium*: BS2-C3). Larvae in the control were exposed to a 0.05% Tween 80 solution. Treatments followed by different letters are significantly different (log-rank test, α = 5%).

Eight isolates selected from the preliminary screening were tested in larvicidal assays against *C. pipiens molestus* larvae. Treatment significantly affected larval survival (Kaplan–Meier: χ^2^ = 259, df = 8, *P *< 0.001). The control showed the highest survival (*P *≤ 0.001 against all fungal treatments; Fig. 1). Isolate BS2-C3 (*Metarhizium* sp.) showed the highest efficacy (*P *< 0.001 against other treatments), with over 90% mortality, an LT_50_ of 1.8 d (CI: 1.5 to 2.1) and an LT_90_ of 4.6 d (CI: 3.8 to 5.4). BS1-C4 (*Penicillium* sp.) was the only other isolate to exceed 50% mortality, with an LT_50_ of 5.4 d (CI: 4.3 to 6.9). The remaining isolates showed no significant differences from each other (*P *> 0.05 for all pairwise comparisons).

#### Primary Screening Against Adult Mosquitoes

**Fig. 2.**
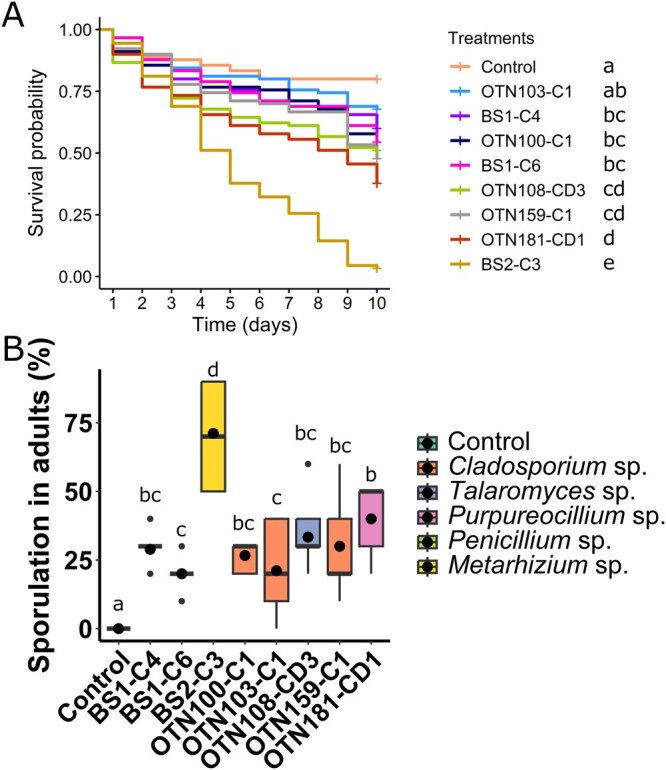
Primary adulticidal screening. A) Daily cumulative survival probability (Kaplan–Meier) of *Culex pipiens molestus* adults exposed to conidial suspensions (10^8^ conidia/ml; dose 2 × 10^8^ conidia/ml) of different fungi. Adults in the control were exposed to a 0.05% Tween 80 solution. B) Percentage of sporulation in adult cadavers. Treatments were compared using a binomial generalized linear mixed model followed by pairwise comparisons of estimated marginal means with FDR-adjusted *P*-values. Boxes show the median, 25th, and 75th percentiles; dots represent treatment means. In each graph, treatments followed by different letters are significantly different (α = 5%). No sporulation was observed in the control group. Fungal genera: *Cladosporium* (OTN100-C1, OTN103-C1, OTN159-C1 and BS1-C6); *Talaromyces* (OTN108-CD3); *Purpureocillium* (OTN181-CD1); *Penicillium* (BS1-C4); *Metarhizium* (BS2-C3).

The 8 selected isolates were also tested against adults of *C. pipiens molestus*. There was a significant effect of treatment on adult survival (χ^2^ = 154, df = 8, *P *< 0.001). The control and isolate OTN103-C1 (*Cladosporium* sp.) showed the highest survival, with no significant difference between them (*P *= 0.145; Fig. 2A). Isolate BS2-C3 (*Metarhizium* sp.) caused the highest mortality (∼97%; *P *< 0.001 against all treatments), with an LT_50_ of 4.1 d (CI: 3.4 to 4.8) and an LT_90_ of 10.4 (CI: 8.8 to 12.4), followed by OTN181-CD1 (*Purpureocillium* sp.), with ∼62% mortality and LT_50_ of 7.7 d (CI: 6.3 to 9.5).

Sporulation data were fitted to a binomial GLMM, with treatment as a fixed effect, and a random intercept for biological replicate. The control treatment was excluded from the model because no sporulation was observed in this group, which caused complete separation and prevented model convergence. Among the 8 fungal treatments included in the model, there was a significant effect of treatment (χ^2^ = 74.52; df = 7; *P *< 0.001; Fig. 2B), with isolate BS2-C3 (*Metarhizium* sp.) showing the highest mean sporulation, exceeding 70% (*P *< 0.001 against all treatments). In contrast, isolates BS1-C6 and OTN103-C1 (both *Cladosporium* sp.) showed the lowest mean sporulation, not differing from each other (*P *= 0.868). The remaining isolates showed similar mean sporulation levels, not differing from BS1-C6 and OTN103-C1 (*P *> 0.05 for all comparisons), except for OTN181-CD1 (*Purpureocillium* sp.), which had significantly higher sporulation than both (*P *= 0.012 and *P *= 0.018, respectively). The control was compared separately to each fungal treatment, confirming that all fungal isolates significantly differed from it (*P *< 0.001 for all comparisons).

#### Adulticidal Activity–Characterization of BS2-C3 (*Metarhizium* sp.)

**Fig. 3.**
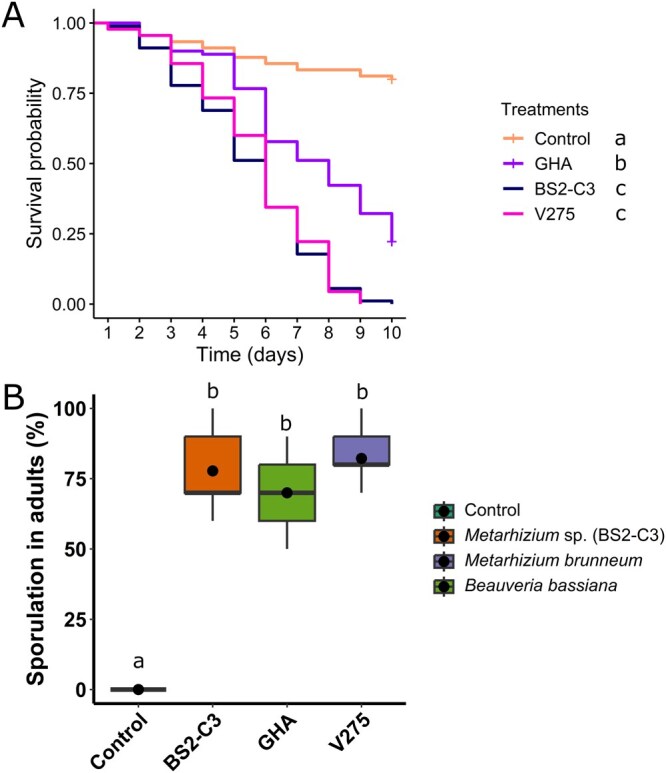
Adulticidal assay comparing isolate BS2-C3 with commercial isolates. A) Daily cumulative survival probability (Kaplan–Meier) of *Culex pipiens molestus* adults exposed to conidial suspensions (10^8^ conidia/ml; dose 2 × 10^8^ conidia/ml) of *Metarhizium* sp. (BS2-C3), *Metarhizium brunneum* (V275) or *Beauveria bassiana* (GHA). Adults in the control were exposed to a 0.05% Tween 80 solution. B) Percentage of sporulation in adult cadavers. Treatments were compared using a binomial generalized linear model followed by pairwise comparisons of estimated marginal means with FDR-adjusted *P*-values (α = 5%). Boxes show the median, 25th, and 75th percentiles; dots represent treatment means. In each graph, treatments followed by different letters are significantly different (α = 5%). No sporulation was observed in the control group.

Based on the results from the primary adulticidal screening, BS2-C3 (*Metarhizium* sp.) was selected for further adulticidal assays, comparing its performance against commercial isolates GHA (*B. bassiana*) and V275 (*M. brunneum*). Mosquito survival differed significantly among treatments (χ^2^ = 182, df = 3, *P *< 0.001). Controls exhibited the highest survival (*P *< 0.001 against all fungal treatments). The 2 *Metarhizium* isolates (BS2-C3 and V275) caused 100% of mortality and did not differ from each other (*P *= 0.55); BS2-C3 had an LT_50_ of 5.4 d (CI: 5.0 to 5.8) and an LT_90_ of 8.2 d (CI: 7.6 to 8.8), and V275 had an LT_50_ of 5.5 d (CI: 5.1 to 5.9) and LT_90_ of 8.4 d (CI: 7.8 to 9.0). Both isolates were significantly more effective than GHA (∼78% mortality; *P *< 0.001) (Fig. 3A), which had an LT_50_ of 7.7 d (CI: 7.1 to 8.4).

Sporulation data were best described by a GLM with binomial distribution, with treatment as a fixed effect. The control was excluded from the model due to absence of sporulation, which would have caused complete separation and prevented model convergence. Among the 3 fungal isolates, there were no differences in sporulation, with all isolates achieving similar levels (χ^2^ = 3.82; df = 2; *P *= 0.15; Fig. 3B). The control was compared separately to each fungal isolate, confirming that all isolates had significantly higher sporulation than it (*P *< 0.001 for all comparisons).

## Discussion

The present study was designed as a targeted screening effort to identify fungal isolates with potential for mosquito control. We evaluated isolates based on their efficacy against larval and adult stages of *C. pipiens molestus*, as well as their capacity for conidial production. By combining broad environmental sampling with application-oriented selection criteria, this approach allowed us to identify suitable candidates for further development of mycoinsecticides.

The larvicidal assays revealed BS2-C3 (*Metarhizium* sp.) as the most effective isolate, causing over 90% mortality within 7 d, with an LT_50_ of less than 2 d and an LT_90_ of less than 5 d. This aligns with previous reports of *B. bassiana*, *Metarhizium anisopliae* (Metsch.) Sorokin and *M. brunneum* achieving 90% to 100% larval mortality against *Anopheles gambiae* Giles, *Aedes aegypti* (L.), *Anopheles stephensi* Liston, and *Culex quinquefasciatus* Say, at comparable concentrations (Greenfield et al. 2015, Tawidian et al. 2023, Ribeiro et al. 2024).

Isolate BS1-C4 (*Penicillium* sp.) was the only other isolate that caused over 50% larval mortality. Although *Penicillium* is not traditionally considered an entomopathogenic genus, there is evidence that some species exhibit insecticidal properties, which could explain the moderate efficacy observed here. However, reported larvicidal activity of *Penicillium* species varies widely, ranging from 0% to 100% mortality, depending on the host species and fungal strain (Costa et al. 1998). Such variability in efficacy suggests that mortality may be mediated by secondary metabolites rather than direct infection, as many of these compounds have demonstrated insecticidal activity against a range of insect pests (Nicoletti et al. 2023).

Similarly to BS1-C4 (*Penicillium* sp.), the other isolates tested also showed lower efficacy to *C. pipiens molestus* larvae compared to BS2-C3 (*Metarhizium* sp.), possibly reflecting interspecific differences in pathogenicity or production of insecticidal metabolites, as well as larval feeding behavior. *Culex* larvae feed in the water column (“collecting-filtering”) (Merritt et al. 1992), therefore, it is plausible that larvae fed on suspended conidia and died as a result of damage to the midgut and possibly the presence of toxins (Bitencourt et al. 2023). As conidia settled, the larvae would be less likely to encounter and feed on them due to their preferential browsing habits within the water column. Even for “sediment-grazing” mosquito larvae, which feed at the bottom, such as *Aedes* spp., loss of efficacy within days of deposition has been reported (Alkhaibari et al. 2023), highlighting the need for suitable formulation or application methods to maintain fungal persistence and avoid the need for frequent reapplications.

Isolate BS2-C3 (*Metarhizium* sp.) also demonstrated strong efficacy against adult mosquitoes, causing approximately 97% mortality within 10 d. This level of activity matches previous findings in which mortalities above 80% were reported for adult *C. quinquefasciatus*, *An. stephensi*, and *An. gambiae* exposed to *M. anisopliae*, *M. brunneum* (reported as *M. anisopliae*) or *B. bassiana* (Scholte et al. 2003, Blanford et al. 2005, Popko et al. 2018).

The second most effective isolate in our adulticidal assay, OTN181-CD1 (*Purpureocillium* sp.), caused approximately 62% mortality, consistent with evidence that *Purpureocillium lilacinum* (Thom) Luangsa-ard, Houbraken, Hywel-Jones & Samson (formerly *Paecilomyces lilacinus*) can also infect and kill insects, despite being better known for its nematicidal activity (Toledo-Hernández et al. 2019, Thi et al. 2023). *Purpureocillium lillacinum* has also shown strong ovicidal activity against *Ae. aegypti* (Luz et al. 2007) and produces secondary metabolites capable of blocking malaria transmission in assays with *Plasmodium falciparum* (Welch) and infected *An. gambiae* (Niu et al. 2021, 2024). Such multifunctional activity suggests that *Purpureocillium* isolates such as OTN181-CD1 may represent promising candidates for development as multifunctional mosquitocides and for integrated use across mosquito life stages in IVM programs. Furthermore, its mosquitocidal activities highlight the value of extending screening efforts beyond classical EPF, given that isolates from other genera with only moderate direct efficacy can still contribute significantly to control strategies through complementary traits.

The efficacy observed for the *Cladosporium* (33% to 48% larval mortality, 32% to 52% adult mortality) and *Talaromyces* (38% larval mortality, 49% adult mortality) isolates indicates limited potential as mosquitocides via direct infection. Although these genera are rarely evaluated for insecticidal activity, their inclusion in our study provides a comparative baseline for future screens; wider efforts to investigate underexplored genera may reveal more efficacious isolates or species that could warrant further investigation. In addition, reports of insecticidal activity from secondary metabolites produced by *Cladosporium* and *Talaromyces* (Castillo et al. 1999, Hayashi et al. 2012a, 2012b, Vinale et al. 2017, Shaker et al. 2019, Wang et al. 2023) suggest that their potential may lie not in direct infection, but in the production of bioactive compounds. Accordingly, these isolates may represent valuable candidates for future metabolite-focused screening approaches, complementing traditional whole-organism bioassays and expanding the scope of fungal-based mosquito control strategies.

Current mosquito control methods employ strains of *Bacillus thuringiensis* subsp. *israelensis* (Bti) and *Lysinibacillus sphaericus* (Meyer and Neide) Ahmed et al. and spinosyns, which cause rapid mortality, usually reaching 100% within 24 h (Nartey et al. 2013, Rojas-Pinzón and Dussán 2017, Su and Liu 2025). However, Bti exhibits limited persistence in the environment (Lacey et al. 2015), and mosquito resistance to *L. sphaericus* and spinosyns has been documented (Su et al. 2018, 2019, Su and Liu 2025), which could restrict long-term efficacy of these control methods. In contrast, despite their slower action, EPF can offer advantages, as they can persist longer in the environment through survival on treated surfaces and recycling via sporulation on cadavers, and resistance has rarely been reported (Dubovskiy et al. 2013, Sharma et al. 2023). These characteristics highlight EPF as complementary agents with distinct advantages, especially in situations where persistence and resistance to conventional methods limit the effectiveness of existing tools.

Variation in sporulation may arise from natural variability among fungal species and isolates, host nutrient content, or physiological condition of the fungus (Sawyer et al. 1997). Additionally, the composition of fatty acids in the host cuticle can reduce or even inhibit sporulation (Boguś et al. 2010). Our data suggest that BS2-C3 (*Metarhizium* sp.) may be better at overcoming such barriers than the other isolates tested, as its sporulation rate exceeded 70%, while the other isolates ranged between 20% to 40%. High sporulation on dead hosts increases pathogen density in the environment, facilitating the occurrence of epizootics and horizontal transmission (Shapiro-Ilan et al. 2012). From a biocontrol perspective, this trait might compensate for a slower kill rate by ensuring that the fungus remains present in the environment for a longer time.

The results from the comparative adulticidal assay highlight the strong efficacy of BS2-C3 (*Metarhizium* sp.), which was more effective than isolate GHA (*B. bassiana*) and showed comparable efficacy to isolate V275 (*M. brunneum*). While its laboratory efficacy equals that of established commercial isolates, the identification of BS2-C3 expands the pool of fungal candidates available for mosquito control. Distinct isolates often vary in traits such as persistence, tolerance to environmental factors, or compatibility with formulation technologies, all of which can influence their performance under field conditions; these differences could make BS2-C3 particularly suitable for certain environments or control strategies. In this context, multi-strain comparative and combination-based screening represents a promising avenue for further research. Previous studies have shown that mixtures of EPF can result in additive, neutral, or antagonistic effects depending on the specific isolates involved (Cruz et al. 2006, Seid et al. 2019). Therefore, systematic evaluation of isolate combinations, particularly among strains with complementary traits (eg high virulence, environmental tolerance, or sporulation capacity), could help identify formulations with improved overall performance compared to single-strain applications.

In summary, our study revealed the presence of diverse fungal taxa in the Cretan environment, including both well-known and less-studied genera. Through targeted isolation and screening, we identified BS2-C3 (*Metarhizium* sp.) with consistent high efficacy against both larvae and adults of *C. pipiens molestus*, and performance comparable to or exceeding that of commercial EPF strains under laboratory conditions. These findings highlight its potential for mosquito control, warranting further evaluation under laboratory, semi-field and field conditions, against multiple mosquito vector species. Its integration into IVM programs (eg through larval source management, application in adult resting surfaces, or incorporation into attract-and-kill devices) could complement existing methods (eg IRS, ITNs), while reducing reliance on chemical insecticides. Furthermore, the moderate activity observed for isolates of *Penicillium*, *Purpureocillium*, *Cladosporium*, and *Talaromyces* suggests that a broader spectrum of fungal genera may harbor biocontrol potential, possibly mediated by secondary metabolites which could be formulated or enhanced to provide greater killing activity. Continued exploration to identify novel insecticidal fungi will be essential for developing effective, safe, and environmentally sustainable alternatives to chemical insecticides in IVM programs.

## Supplementary Material

tjag069_Supplementary_Data

## Data Availability

Data relating to bioassays conducted within the study can be found in the Figshare repository at doi.org/10.6084/m9.figshare.30186355. ITS sequencing data can be found at GenBank under the accession numbers PX136791–PX136836.
