# Supplementary material for: Screening of environmental fungi from Crete reveals candidates for biological control of mosquitoes
Source: J Med Entomol. 2026 May 26;63(3):tjag069. doi: 10.1093/jme/tjag069 (PMC13202464; doi:10.1093/jme/tjag069)
Supplement: tjag069_Supplementary_Data [file tjag069_supplementary_data.zip › Supplementary Material S2.docx]

**Supplemental Material S2.** GenBank and UNITE accession numbers used for multiple sequence alignments and construction of phylogenetic trees of each isolate obtained in the present study. Codes in bold are only available at the UNITE database.

| **Isolates** | **GenBank/UNITE accession numbers** | | | | | |
| --- | --- | --- | --- | --- | --- | --- |
| OTN24-CD4 | MT889844 | MT889849 | MZ568177 | MZ568183 | OP970632 | OR144614 |
|  | MT889845 | MT889850 | MZ568178 | OM218650 | OR144610 | PP464180 |
|  | MT889846 | MT889851 | MZ568179 | OM218654 | OR144611 | PP464181 |
|  | MT889847 | MW729033 | MZ568180 | OM349612 | OR144612 | PP550715 |
|  | MT889848 | MZ568176 | MZ568182 | OP681423 | OR144613 | PP731792 |
| OTN58-CD1 | KY788056 | MF473139 | MK360772 | MK761390 | MW729033 | OR197591 |
|  | KY788058 | MH329777 | MK392007 | MK793189 | ON127865 | PP464176 |
|  | MF077230 | MK111468 | MK690546 | MK818535 | ON127895 | PP464180 |
|  | MF077231 | MK267741 | MK722284 | MK818536 | OP473942 | PP464181 |
|  | MF189920 | MK360768 | MK722286 | MK818537 | OP681423 | PP829042 |
| OTN75-CD1 | MZ568193 | OM415933 | OP178987 | OP584636 | OW985322 | PQ400032 |
|  | MZ568194 | OM415937 | OP179013 | OP584640 | OW986725 | PQ480489 |
|  | MZ568195 | OM877504 | OP584623 | OP970627 | OW987343 | PQ480509 |
|  | OK169485 | OP178980 | OP584630 | OQ236707 | PQ148138 | PQ480524 |
|  | OK274325 | OP178982 | OP584635 | OW983063 | PQ357203 | PQ480550 |
| OTN83-CD5 | GQ911533 | MH399294 | MT821134 | OQ852940 | PP844334 | PQ453757 |
|  | JX188091 | MT112985 | MT862363 | PP580138 | PP844344 | PQ772268 |
|  | JX188092 | MT112987 | MW791899 | PP844179 | PP903744 | PQ796723 |
|  | KP335640 | MT230467 | MW791901 | PP844285 | PP905602 | **UDB028483** |
|  | KX249734 | MT520596 | OK376912 | PP844325 | PQ453750 | **UDB035654** |
| OTN99-C4 | MW447008 | MZ078505 | OK376903 | OK376923 | OK376995 | OP699767 |
|  | MW798756 | MZ823817 | OK376907 | OK376924 | OK661039 | OP699768 |
|  | MW862793 | OK376886 | OK376920 | OK376934 | OM363458 | OP699769 |
|  | MZ078503 | OK376901 | OK376921 | OK376935 | OM363474 | OQ589895 |
|  | MZ078504 | OK376902 | OK376922 | OK376940 | OM363485 | OR392753 |
| OTN100-C1 | KP942857 | PP758283 | PP896549 | PQ037791 | PQ357213 | PQ512104 |
|  | MG669180 | PP800754 | PP896551 | PQ065981 | PQ358272 | PQ584381 |
|  | MG840754 | PP844873 | PP903752 | PQ164571 | PQ358276 | PQ620098 |
|  | PP736376 | PP860258 | PP903779 | PQ213375 | PQ362744 | PQ626053 |
|  | PP736377 | PP860319 | PQ037774 | PQ340158 | PQ453768 | **UDB0799158** |
| OTN102-C2 | MZ568193 | OM415933 | OP178987 | OP584636 | OW985322 | PQ400032 |
|  | MZ568194 | OM415937 | OP179013 | OP584640 | OW986725 | PQ480489 |
|  | MZ568195 | OM877504 | OP584623 | OP970627 | OW987343 | PQ480509 |
|  | OK169485 | OP178980 | OP584630 | OQ236707 | PQ148138 | PQ480524 |
|  | OK274325 | OP178982 | OP584635 | OW983063 | PQ357203 | PQ480550 |
| OTN103-C1 | AJ875362 | JF289132 | KC009539 | KT898690 | MF473134 | MH473978 |
|  | JF289128 | JF289134 | KP701901 | KX674650 | MF473135 | MK095991 |
|  | JF289129 | JF289138 | KT600401 | KY039304 | MF473136 | MK267741 |
|  | JF289130 | JF289139 | KT600402 | KY781780 | MF473137 | MT773569 |
|  | JF289131 | JQ946406 | KT898610 | LC775699 | MF473139 | **UDB0799156** |
| OTN108-CD3 | MK367725 | MK367741 | MK367771 | MN944724 | MT529938 | OM049426 |
|  | MK367729 | MK367753 | MK367772 | MT252503 | MT530049 | OW985396 |
|  | MK367731 | MK367754 | MK808539 | MT529070 | OK087306 | OW988421 |
|  | MK367735 | MK367758 | MN393465 | MT529283 | OK584745 | PP544886 |
|  | MK367740 | MK367770 | MN427868 | MT529653 | OK632392 | PP544887 |
| OTN122-CD1 | FN391354 | FN397313 | HG936566 | KX058068 | MW268806 | OR581594 |
|  | FN391356 | FN397316 | HG936567 | KX343151 | OQ338848 | OR690334 |
|  | FN391357 | FN689671 | KF385305 | MT453274 | OQ726135 | OR817763 |
|  | FN391358 | FN689675 | KF385340 | MT521795 | OQ726139 | OU989405 |
|  | FN397151 | FN812729 | KR709197 | MT913535 | OR206530 | PQ775903 |
| OTN123-C3 | AB236002 | GU246247 | KY103598 | KY103605 | MG554339 | MT892805 |
|  | DQ683006 | KP132303 | KY103599 | MG554278 | MG554358 | MZ422983 |
|  | EF060697 | KP132304 | KY103600 | MG554280 | MG554362 | MZ962715 |
| **Isolates** | **GenBank/UNITE accession numbers** | | | | | |
| OTN123-C3 | EF060719 | KY103596 | KY103603 | MG554321 | MH855890 | MZ962716 |
|  | EU714323 | KY103597 | KY103604 | MG554334 | MH855891 | OW987767 |
| OTN123-C4 | KF411588 | MG920350 | MZ509349 | OL913887 | OR264325 | **UDB01088357** |
|  | KF798202 | MH860502 | OL457280 | OL913895 | OR264337 | **UDB01088361** |
|  | KU293682 | MH931822 | OL913882 | OL913896 | OR564024 | **UDB0780402** |
|  | LT549076 | MT658105 | OL913883 | OM219630 | OW983833 | **UDB0780403** |
|  | MF788188 | MW826176 | OL913884 | OP161502 | **UDB01088321** | **UDB0780408** |
| OTN123-CD4 | JN837088 | JN837094 | KP132577 | KX017571 | MK679593 | MW710681 |
|  | JN837090 | JQ026367 | KU350344 | KY104788 | MK679594 | MW710786 |
|  | JN837091 | JX188230 | KU350345 | KY104789 | MN128863 | NR_073282 |
|  | JN837092 | KJ706313 | KU350350 | LC390312 | MW710630 | OM648204 |
|  | JN837093 | KM014593 | KX017570 | LC390313 | MW710670 | OQ448346 |
| OTN128-C1 | PQ328681 | PQ350469 | PQ350477 | PQ482027 | PQ584392 | **UDB025064** |
|  | PQ340514 | PQ350470 | PQ350483 | PQ482028 | PQ636917 | **UDB035253** |
|  | PQ340515 | PQ350471 | PQ350669 | PQ584344 | PQ655483 | **UDB035663** |
|  | PQ350464 | PQ350472 | PQ380917 | PQ584388 | PQ655485 | **UDB038366** |
|  | PQ350467 | PQ350473 | PQ381655 | PQ584390 | PQ655486 | **UDB0802795** |
| OTN128-CD4 | MK818535 | MT889836 | MT889846 | MT889851 | MZ568179 | OM218654 |
|  | MK818536 | MT889840 | MT889847 | MW729033 | MZ568180 | OP681423 |
|  | MK818537 | MT889841 | MT889848 | MZ568176 | MZ568182 | PP464180 |
|  | MN114156 | MT889844 | MT889849 | MZ568177 | MZ568183 | PP464181 |
|  | MT889834 | MT889845 | MT889850 | MZ568178 | OM218650 | PP731792 |
| OTN129-CD5 | MT461156 | MW269383 | OP178977 | OR019744 | OU989447 | OZ012221 |
|  | MW269120 | MW364392 | OR019723 | OR267517 | OU989452 | PP703024 |
|  | MW269121 | MZ145249 | OR019734 | OR584329 | OU989453 | PP748238 |
|  | MW269351 | OM906088 | OR019735 | OR752277 | OW987187 | PP766694 |
|  | MW269369 | ON935454 | OR019743 | OR793059 | OW987795 | PP869098 |
| OTN137-C5 | MZ568193 | OM415933 | OP178987 | OP584636 | OW985322 | PQ400032 |
|  | MZ568194 | OM415937 | OP179013 | OP584640 | OW986725 | PQ480489 |
|  | MZ568195 | OM877504 | OP584623 | OP970627 | OW987343 | PQ480509 |
|  | OK169485 | OP178980 | OP584630 | OQ236707 | PQ148138 | PQ480524 |
|  | OK274325 | OP178982 | OP584635 | OW983063 | PQ357203 | PQ480550 |
| OTN139-C2 | MF473012 | PP351391 | PP734741 | PP860319 | PQ065981 | PQ358276 |
|  | MG664760 | PP464178 | PP735259 | PP903752 | PQ164571 | PQ512104 |
|  | PP100120 | PP464179 | PP736376 | PP903779 | PQ213375 | PQ584381 |
|  | PP156760 | PP496186 | PP736377 | PQ037774 | PQ340158 | PQ620098 |
|  | PP351370 | PP523931 | PP800754 | PQ037791 | PQ357213 | PQ626053 |
| OTN145-C3 | FN397316 | HG936567 | KX009142 | MT453274 | ON038743 | OR206530 |
|  | FN689671 | KF385305 | KX058068 | MT521795 | ON038748 | OR581594 |
|  | FN689675 | KF385340 | KX343172 | MT913535 | ON193796 | OU989405 |
|  | FN812729 | KR709197 | MG189953 | MW268806 | OQ726135 | PP794882 |
|  | HG936566 | KT582070 | MG189954 | MW268810 | OQ726139 | PQ775903 |
| OTN145-CD5 | AF037435 | HQ608058 | KC344974 | MH045584 | OL764382 | OQ130722 |
|  | AF037436 | HQ646588 | KF381077 | MH045589 | OL764385 | OW988192 |
|  | HG995517 | HQ646589 | KX009143 | MH860152 | OM236508 | PP385372 |
|  | HG996103 | KC344972 | LR994022 | MW301155 | OM964582 | PP851682 |
|  | HQ607978 | KC344973 | MH029828 | OL703505 | OP237299 | PQ358850 |
| OTN148-C1 | PP829042 | PP844370 | PP959230 | PQ415893 | PQ480509 | PQ556188 |
|  | PP844303 | PP844394 | PQ305589 | PQ415931 | PQ480524 | PQ608602 |
|  | PP844306 | PP844441 | PQ350587 | PQ415995 | PQ480546 | PQ657562 |
|  | PP844311 | PP869094 | PQ357203 | PQ454688 | PQ480550 | PQ676541 |
|  | PP844368 | PP959222 | PQ400032 | PQ480489 | PQ480562 | **UDB0799152** |
| OTN149-C4 | MF473012 | OU989333 | OU989340 | OW988083 | PP523931 | PP903779 |
|  | OU989321 | OU989334 | OW982406 | PP097797 | PP734741 | PQ037774 |
|  | OU989324 | OU989335 | OW987312 | PP156760 | PP735259 | PQ037791 |
|  | OU989331 | OU989338 | OW987346 | PP464178 | PP800754 | PQ164571 |
|  | OU989332 | OU989339 | OW988059 | PP464179 | PP903752 | PQ512104 |
| **Isolates** | **GenBank/UNITE accession numbers** | | | | | |
| OTN150-C1 | PP844303 | PP844394 | PQ148138 | PQ415893 | PQ480509 | PQ556188 |
|  | PP844306 | PP844441 | PQ305589 | PQ415931 | PQ480524 | PQ608602 |
|  | PP844311 | PP869094 | PQ350587 | PQ415995 | PQ480546 | PQ657562 |
|  | PP844368 | PP959222 | PQ357203 | PQ454688 | PQ480550 | PQ676541 |
|  | PP844370 | PP959230 | PQ400032 | PQ480489 | PQ480562 | **UDB0799152** |
| OTN151-C1 | MF473012 | PP758283 | PP896549 | PQ037791 | PQ357213 | PQ512104 |
|  | MG664761 | PP800754 | PP896551 | PQ065981 | PQ358272 | PQ584381 |
|  | PP735259 | PP844873 | PP903752 | PQ164571 | PQ358276 | PQ620098 |
|  | PP736376 | PP860258 | PP903779 | PQ213375 | PQ362744 | PQ626053 |
|  | PP736377 | PP860319 | PQ037774 | PQ340158 | PQ453768 | **UDB0799158** |
| OTN153-C12 | OR543629 | OR758880 | OW987848 | PP388894 | PP928999 | PQ584344 |
|  | OR543680 | OR808067 | PP336534 | PP388895 | PQ248570 | PQ584388 |
|  | OR543688 | OR879298 | PP345901 | PP455394 | PQ380917 | PQ584390 |
|  | OR708582 | OR936267 | PP357977 | PP494210 | PQ482027 | PQ584392 |
|  | OR758512 | OW983243 | PP357978 | PP766016 | PQ482028 | **UDB035253** |
| OTN154-C7 | FJ614629 | MG736121 | MT664153 | ON024355 | OP970594 | OQ733296 |
|  | HQ654261 | MH855173 | MT664154 | OP970590 | OP970595 | OQ733297 |
|  | KC254054 | MN493085 | OM955965 | OP970591 | OP970596 | OQ733298 |
|  | KU935671 | MN493099 | OM956030 | OP970592 | OQ402578 | OW988385 |
|  | MG274309 | MN841912 | OM956031 | OP970593 | OQ733295 | PP982547 |
| OTN154-C11 | AF414969 | KU935671 | MN493099 | OM956031 | OP970594 | OQ733296 |
|  | AF414972 | MG274309 | MT664153 | OP970590 | OP970595 | OQ733297 |
|  | FJ614629 | MG736121 | MT664154 | OP970591 | OP970596 | OQ733298 |
|  | HQ654261 | MH855173 | OM955965 | OP970592 | OQ402578 | OW988385 |
|  | KC254054 | MN493085 | OM956030 | OP970593 | OQ733295 | PP982547 |
| OTN158-C1 | JQ724507 | PQ415824 | PQ480448 | PQ577849 | PQ620098 | **UDB035686** |
|  | JX135869 | PQ415894 | PQ480504 | PQ578936 | PQ626053 | **UDB035710** |
|  | KJ589555 | PQ415930 | PQ480532 | PQ584381 | PQ637630 | **UDB0778763** |
|  | KX641958 | PQ453768 | PQ512104 | PQ596434 | PQ647323 | **UDB0780758** |
|  | ON584342 | PQ480444 | PQ564755 | PQ614852 | **UDB027698** | **UDB0799158** |
| OTN159-C1 | MW764317 | PP844441 | PQ305589 | PQ415931 | PQ480524 | PQ584380 |
|  | PP844368 | PP869094 | PQ350587 | PQ415995 | PQ480546 | PQ608602 |
|  | PP844370 | PP959222 | PQ357203 | PQ454688 | PQ480550 | PQ657562 |
|  | PP844394 | PP959230 | PQ400032 | PQ480489 | PQ480562 | PQ676541 |
|  | PP844423 | PQ148138 | PQ415893 | PQ480509 | PQ556188 | **UDB0799152** |
| OTN161-C2 | PQ158794 | PQ158837 | PQ158869 | PQ158924 | PQ168970 | PQ415890 |
|  | PQ158806 | PQ158839 | PQ158877 | PQ158985 | PQ331210 | PQ415956 |
|  | PQ158822 | PQ158848 | PQ158884 | PQ158998 | PQ381061 | PQ415976 |
|  | PQ158833 | PQ158862 | PQ158886 | PQ159006 | PQ381171 | PQ678942 |
|  | PQ158834 | PQ158868 | PQ158918 | PQ159018 | PQ381249 | PQ765241 |
| OTN163-CD1 | GU066613 | KT887847 | KY776338 | MH316141 | MK534497 | OP942450 |
|  | KM066554 | KT887862 | KY776385 | MH316142 | MN701677 | OQ457271 |
|  | KR296913 | KT887864 | KY776421 | MH512953 | MN701678 | OQ784287 |
|  | KT336528 | KT887865 | KY776422 | MH512955 | MN701681 | PQ268904 |
|  | KT887843 | KY776308 | MH316139 | MH512962 | MN701688 | PQ774993 |
| OTN172-C1 | MG664760 | PQ158973 | PQ350443 | PQ358276 | PQ512104 | **UDB027698** |
|  | MG686511 | PQ164571 | PQ350592 | PQ362742 | PQ584381 | **UDB035686** |
|  | MW299369 | PQ213375 | PQ350706 | PQ362743 | PQ596434 | **UDB035710** |
|  | PQ158719 | PQ215955 | PQ357213 | PQ362744 | PQ620098 | **UDB0780758** |
|  | PQ158859 | PQ340158 | PQ358272 | PQ453768 | PQ626053 | **UDB0799158** |
| OTN173-C2 | MF473012 | PP351391 | PP734741 | PP860319 | PQ065981 | PQ358276 |
|  | MG664761 | PP464178 | PP735259 | PP903752 | PQ164571 | PQ512104 |
|  | PP100120 | PP464179 | PP736376 | PP903779 | PQ213375 | PQ584381 |
|  | PP156760 | PP496186 | PP736377 | PQ037774 | PQ340158 | PQ620098 |
|  | PP351370 | PP523931 | PP800754 | PQ037791 | PQ357213 | PQ626053 |
| OTN177-C2 | OP070796 | OP584623 | OP970627 | OR816156 | PQ148138 | PQ480509 |
|  | OP178980 | OP584630 | OQ236707 | OW983063 | PQ350587 | PQ480524 |
| **Isolates** | **GenBank/UNITE accession numbers** | | | | | |
| OTN77-C2 | OP178982 | OP584635 | OQ437288 | OW985322 | PQ357203 | PQ480546 |
|  | OP178987 | OP584636 | OR816154 | OW986725 | PQ400032 | PQ480550 |
|  | OP179013 | OP584640 | OR816155 | OW987343 | PQ480489 | PQ480562 |
| OTN178-CD2 | MW764428 | OK510230 | OP811218 | OU989427 | OU989432 | PQ130143 |
|  | MW764502 | OK510236 | OR335841 | OU989428 | OW983333 | PQ357305 |
|  | MW826135 | OK510237 | OR335845 | OU989429 | OW985574 | **UDB027988** |
|  | MW826164 | OK510276 | OR432196 | OU989430 | PP524001 | **UDB028318** |
|  | MZ078713 | OP178983 | OU989426 | OU989431 | PP809468 | **UDB0780744** |
| OTN178-CD3 | AY373874 | HE615091 | KP329747 | KT291048 | MK793768 | OW985348 |
|  | FJ870911 | HE653030 | KT291003 | KT291071 | MK793770 | OW987722 |
|  | FJ878628 | HE653031 | KT291016 | KX960787 | MN650839 | OW987791 |
|  | FN397275 | KC466531 | KT291041 | MK793766 | OK242755 | OW988123 |
|  | GU594779 | KF367476 | KT291042 | MK793767 | OL631233 | PP549965 |
| OTN179-CD3 | MG686511 | PP903779 | PQ065978 | PQ164571 | PQ512104 | **UDB027698** |
|  | MW299369 | PQ001992 | PQ065981 | PQ213375 | PQ584381 | **UDB035686** |
|  | PP903752 | PQ037774 | PQ158719 | PQ340158 | PQ596434 | **UDB035710** |
|  | PP903766 | PQ037791 | PQ158859 | PQ357213 | PQ620098 | **UDB0780758** |
|  | PP903769 | PQ065969 | PQ158973 | PQ358276 | PQ626053 | **UDB0799158** |
| OTN181-CD1 | GQ229080 | KF800488 | MH864210 | MT530108 | MZ374592 | OP179060 |
|  | HM242264 | KR025540 | MT446187 | MT732891 | MZ374593 | OP788028 |
|  | HQ829056 | KU687114 | MT447527 | MZ374582 | MZ374606 | OQ402321 |
|  | KC157713 | MG857645 | MT453285 | MZ374583 | MZ545388 | OZ012183 |
|  | KF367471 | MG857648 | MT530043 | MZ374588 | OK161079 | OZ012423 |
| OTN182-C1 | PQ584484 | PQ584511 | PQ584529 | PQ584551 | PQ584564 | PQ584577 |
|  | PQ584488 | PQ584513 | PQ584538 | PQ584553 | PQ584565 | PQ584580 |
|  | PQ584493 | PQ584516 | PQ584539 | PQ584555 | PQ584567 | PQ584583 |
|  | PQ584494 | PQ584522 | PQ584541 | PQ584558 | PQ584568 | PQ584584 |
|  | PQ584510 | PQ584525 | PQ584546 | PQ584559 | PQ584575 | PQ584585 |
| OTN185-C6 | OQ713829 | OR145102 | OR690694 | OW987612 | PP702202 | PQ152252 |
|  | OQ789353 | OR259030 | OR899609 | OW988005 | PP702206 | PQ357298 |
|  | OR041599 | OR335219 | OW985022 | OW988416 | PP702213 | PQ373189 |
|  | OR077532 | OR438016 | OW985682 | OZ012240 | PP716877 | PQ614699 |
|  | OR098601 | OR652390 | OW986432 | PP422187 | PQ047489 | PQ637414 |
| OTN185-CD5 | MK818537 | MT889840 | MT889847 | MW729033 | MZ568180 | OP681423 |
|  | MN114156 | MT889841 | MT889848 | MZ568176 | MZ568182 | OP970632 |
|  | MT236941 | MT889844 | MT889849 | MZ568177 | MZ568183 | PP464180 |
|  | MT889834 | MT889845 | MT889850 | MZ568178 | OM218650 | PP464181 |
|  | MT889836 | MT889846 | MT889851 | MZ568179 | OM218654 | PP731792 |
| BS1-C3 | AB158652 | KY103600 | MN310410 | MZ710159 | OW987767 | PQ565378 |
|  | EF568064 | KY103604 | MN787511 | MZ962716 | PQ357300 | PQ565379 |
|  | GU246247 | MG554339 | MT520564 | NR_111253 | PQ565375 | PQ565380 |
|  | KP132302 | MG554358 | MT892805 | OM850419 | PQ565376 | PQ565381 |
|  | KY103597 | MH855891 | MZ422983 | OW984520 | PQ565377 | **UDB0799269** |
| BS1-C4 | AB369902 | DQ339570 | GU566224 | KF367497 | MK793754 | MT732848 |
|  | DQ339550 | DQ681326 | JQ316516 | KT759283 | MK793755 | OM722119 |
|  | DQ339551 | DQ681327 | JX270361 | KX148634 | MK808150 | OW982488 |
|  | DQ339553 | GQ999396 | KF367494 | KX588061 | MK808298 | PP780189 |
|  | DQ339557 | GU566212 | KF367496 | KY859382 | MN431387 | PQ323887 |
| BS1-C6 | MF473012 | PP735259 | PP860258 | PQ037791 | PQ357213 | PQ512104 |
|  | PP464179 | PP736376 | PP860319 | PQ065981 | PQ358272 | PQ584381 |
|  | PP496186 | PP736377 | PP903752 | PQ164571 | PQ358276 | PQ620098 |
|  | PP523931 | PP758283 | PP903779 | PQ213375 | PQ362744 | PQ626053 |
|  | PP734741 | PP800754 | PQ037774 | PQ340158 | PQ453768 | **UDB0799158** |
| BS2-C3 | LR792755 | MF872427 | MT374162 | OR478178 | OR758804 | OR758812 |
|  | LR792769 | MF872428 | OP028070 | OR478179 | OR758805 | OR805437 |
|  | MF063329 | MF872439 | OR052407 | OR478180 | OR758807 | OR805440 |
|  | MF872424 | MT362047 | OR052408 | OR478181 | OR758809 | OR805441 |
| **Isolates** | **GenBank/UNITE accession numbers** | | | | | |
| BS2-C3 | MF872425 | MT372486 | OR478176 | OR478182 | OR758811 | OW982600 |
| BS2-C4 | PQ146539 | PQ200223 | PQ269740 | PQ303239 | PQ516909 | PQ722562 |
|  | PQ151425 | PQ203975 | PQ269741 | PQ303966 | PQ579192 | PQ775893 |
|  | PQ151778 | PQ225953 | PQ269744 | PQ350412 | PQ608600 | PQ788639 |
|  | PQ198235 | PQ232117 | PQ285818 | PQ380174 | PQ660940 | **UDB035210** |
|  | PQ198238 | PQ252957 | PQ285819 | PQ416772 | PQ678783 | X94173 |
