# Supplementary material for: Screening of environmental fungi from Crete reveals candidates for biological control of mosquitoes
Source: J Med Entomol. 2026 May 26;63(3):tjag069. doi: 10.1093/jme/tjag069 (PMC13202464; doi:10.1093/jme/tjag069)

**Supplementary Material S3.** Maximum-likelihood phylogenetic trees of the 46 fungal isolates obtained in the present study. Node labels show bootstrap values calculated from 1,000 replicates, and branch lengths correspond to the number of substitutions per site.

Isolate OTN24-CD4

Substitution model: TN

Outgroup species: *Toxicocladosporium leucadendri*


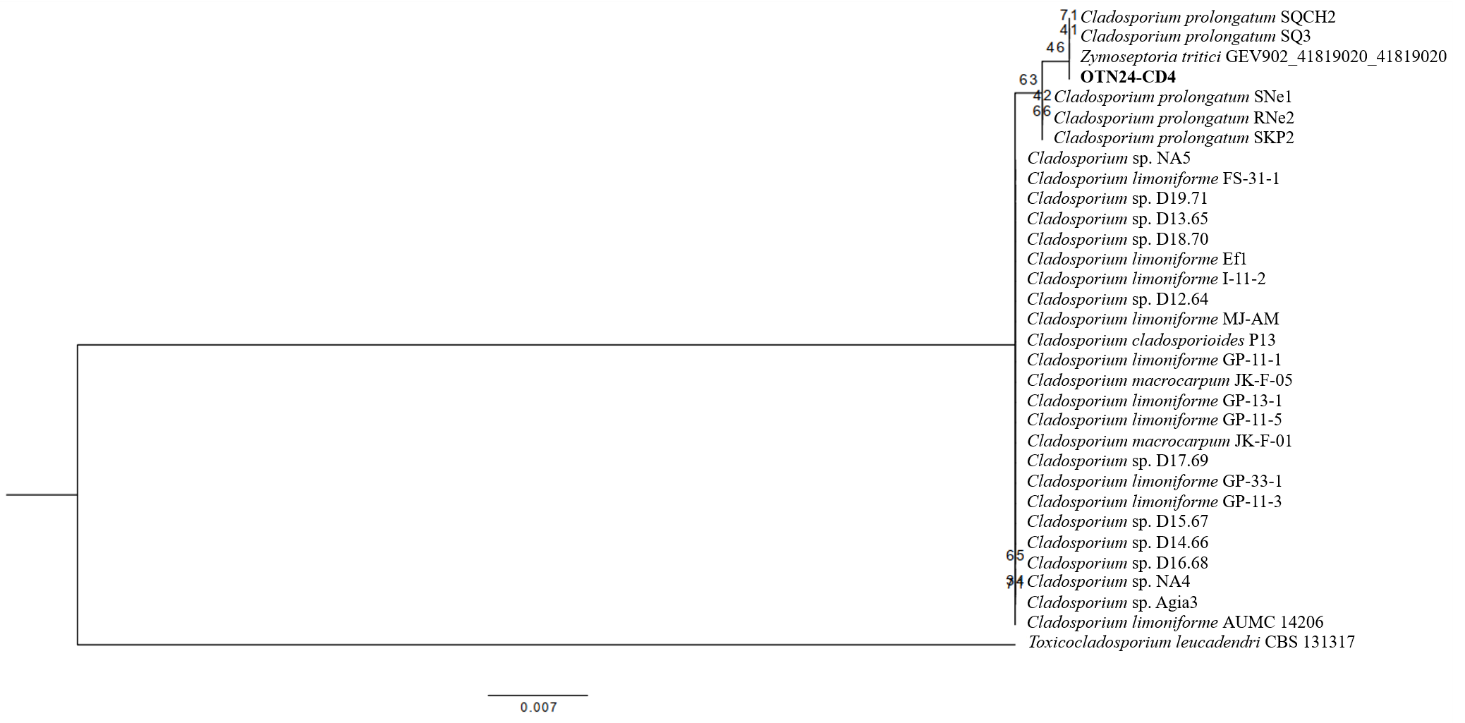


Isolate OTN58-CD1

Substitution model: HKY+R3

Outgroup species: *Toxicocladosporium leucadendri*


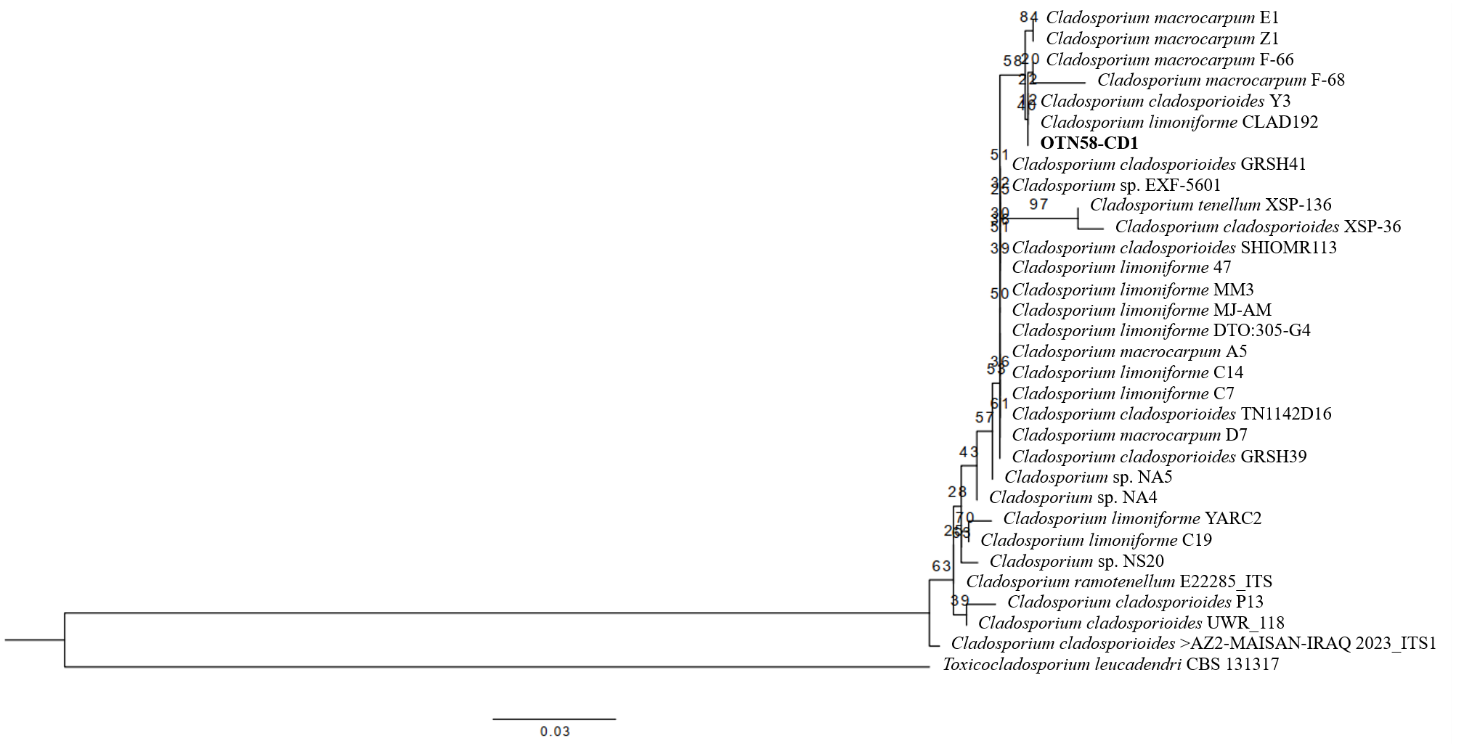


Isolate OTN75-CD1

Substitution model: HKY

Outgroup species: *Toxicocladosporium leucadendri*


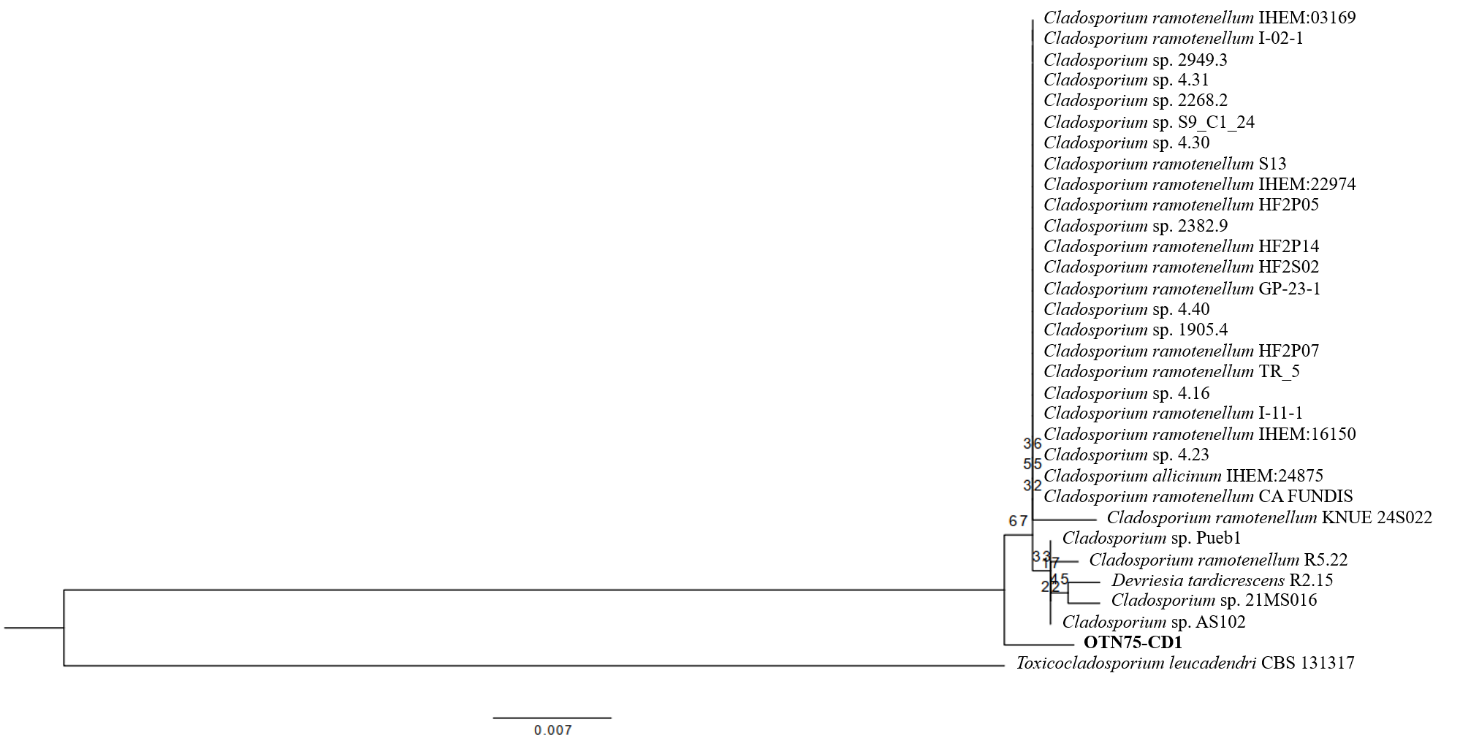


Isolate OTN83-CD5

Substitution model: JC+G4

Outgroup species: *Saccothecium australianum*


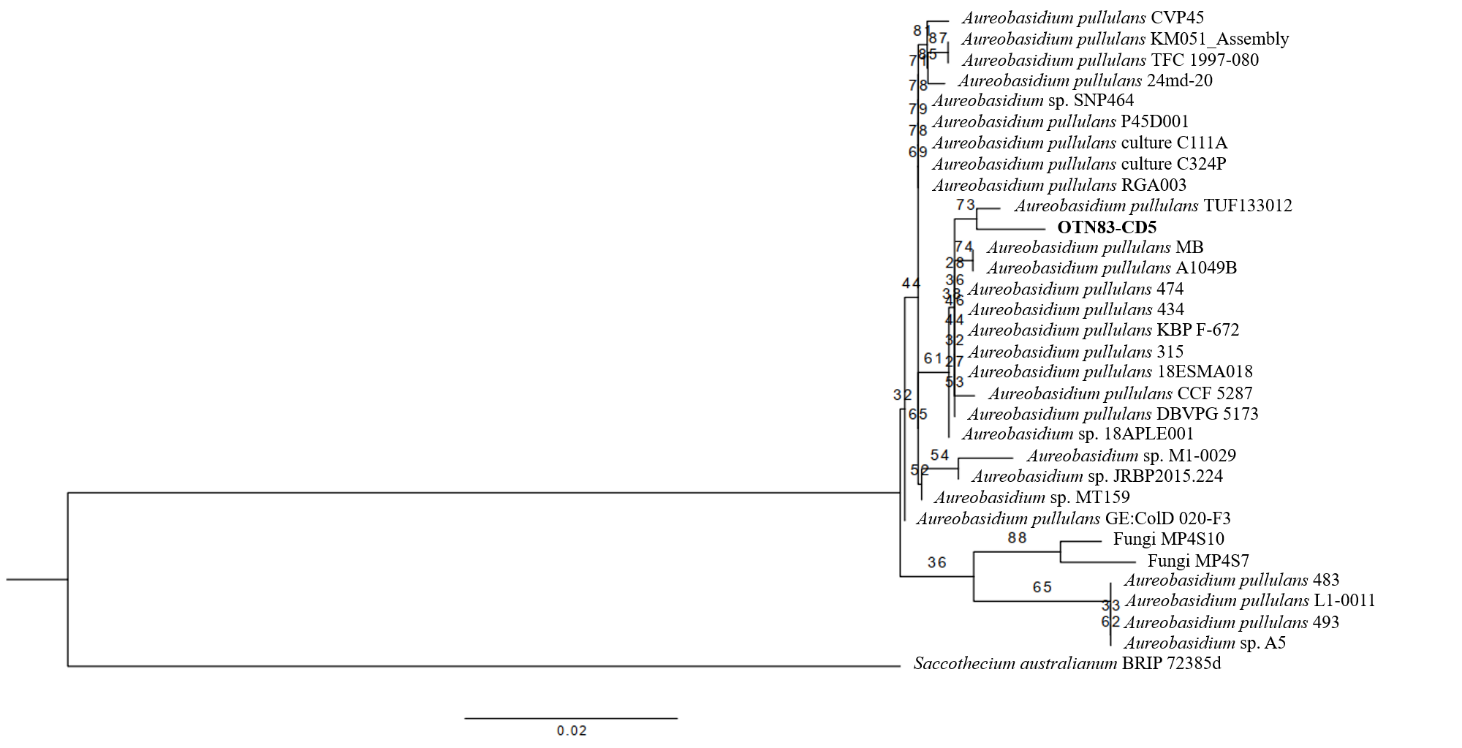


Isolate OTN99-C4

Substitution model: F81

Outgroup species: *Saccothecium australianum*


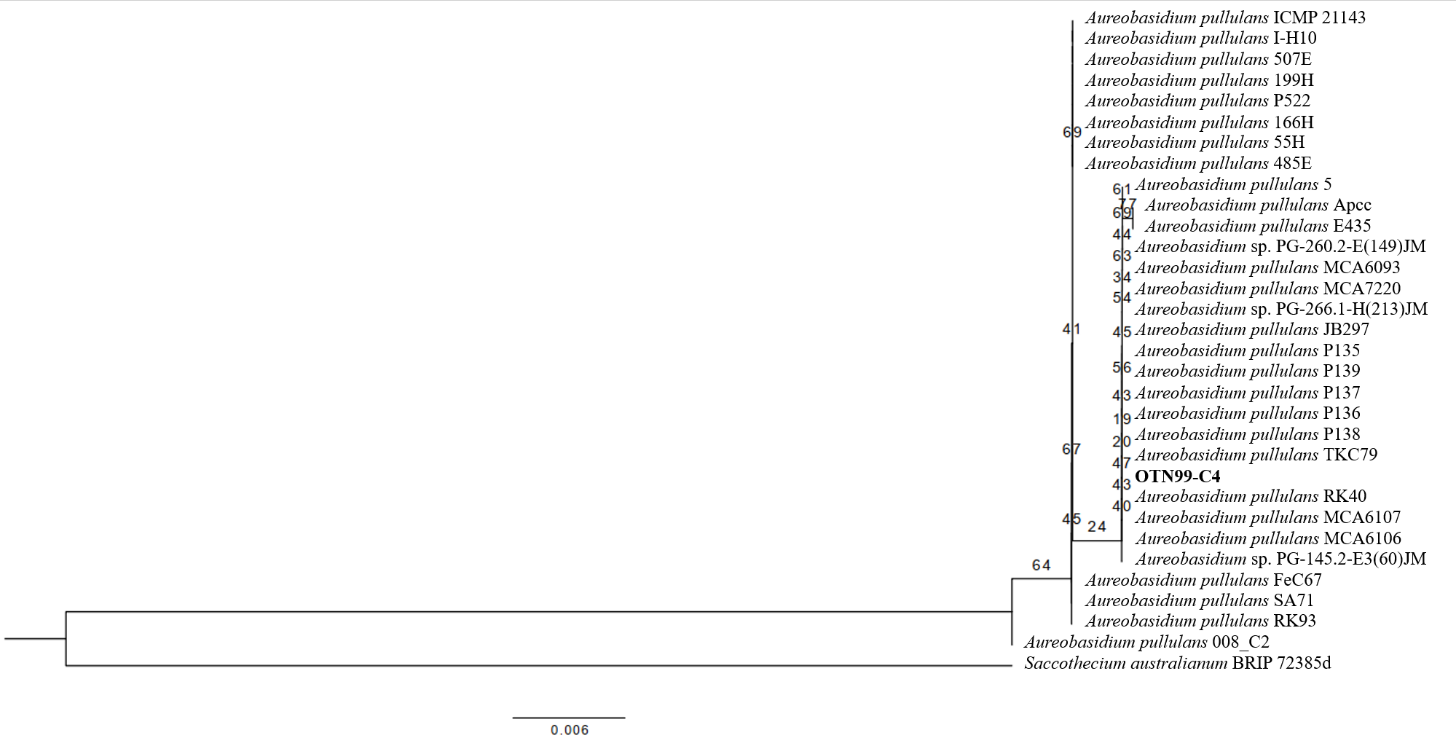


Isolate OTN100-C1

Substitution model: HKY+G4

Outgroup species: *Toxicocladosporium leucadendri*


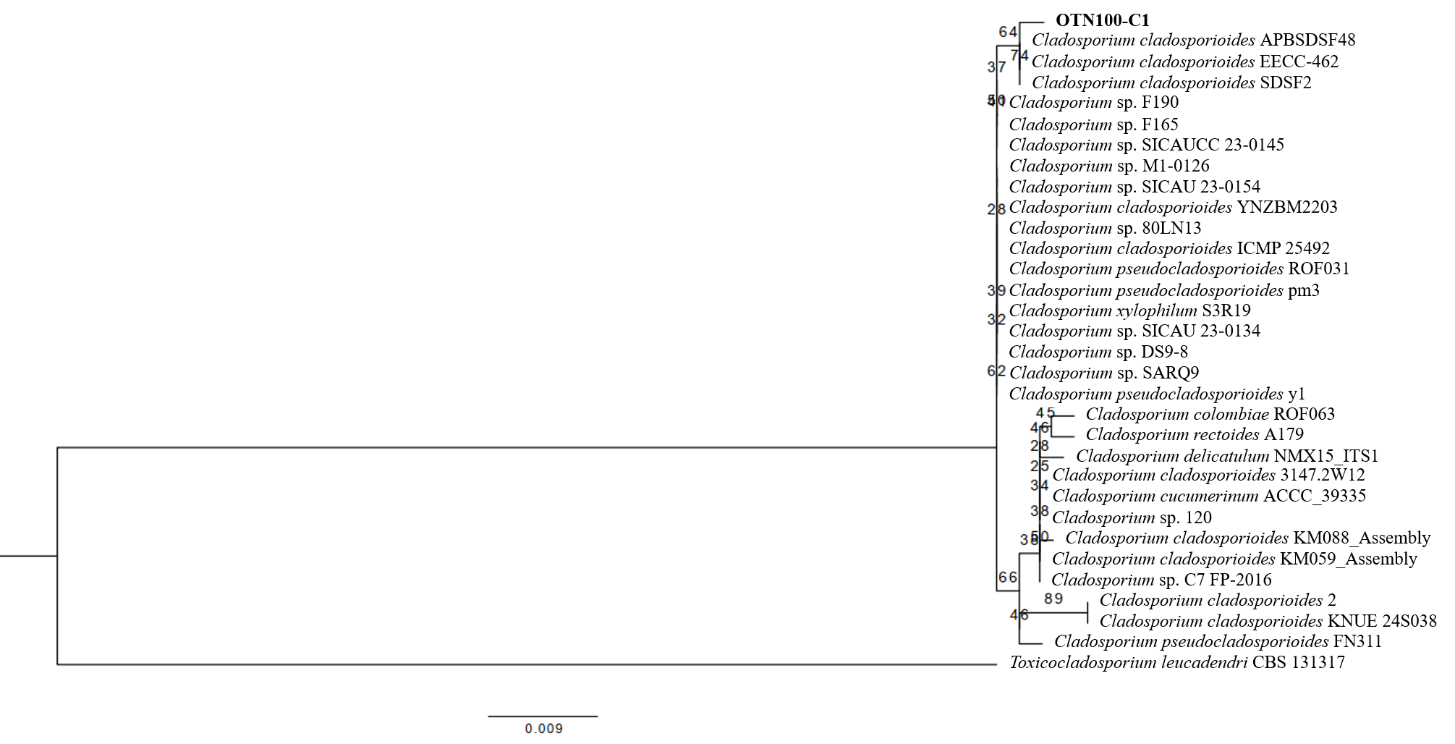


Isolate OTN102-C2

Substitution model: HKY

Outgroup species: *Toxicocladosporium leucadendri*


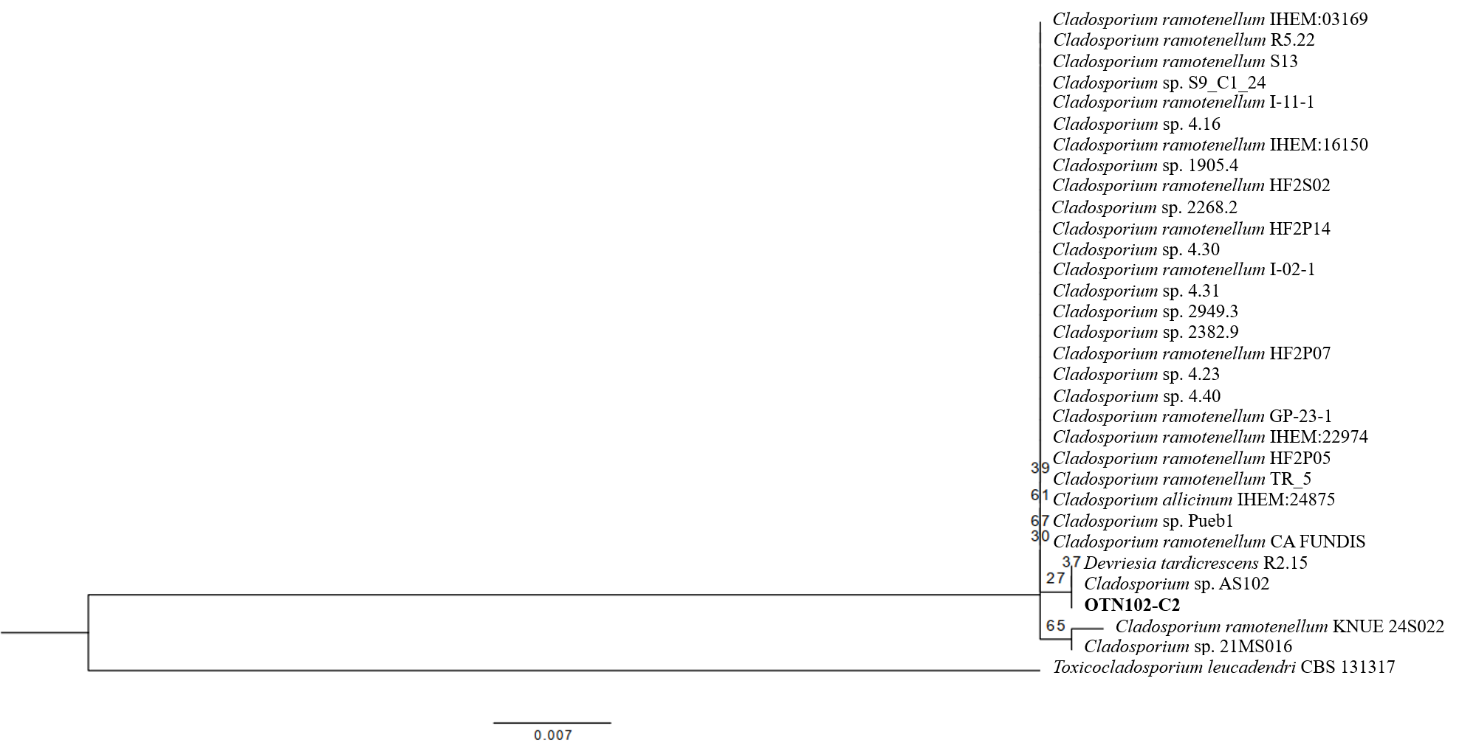


Isolate OTN103-C1

Substitution model: TN

Outgroup species: *Toxicocladosporium leucadendri*


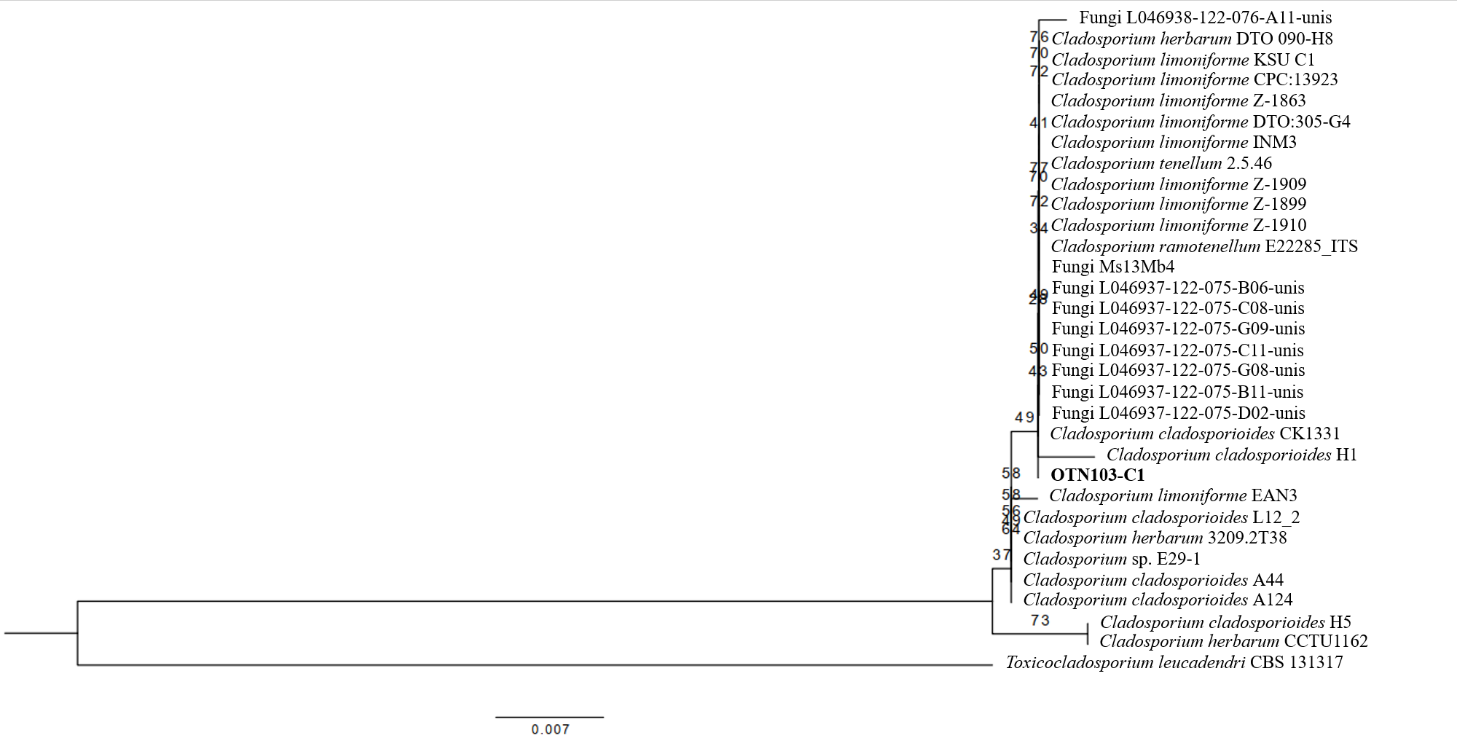


Isolate OTN108-CD3

Substitution model: K2P

Outgroup species: *Penicillium chrysogenum*


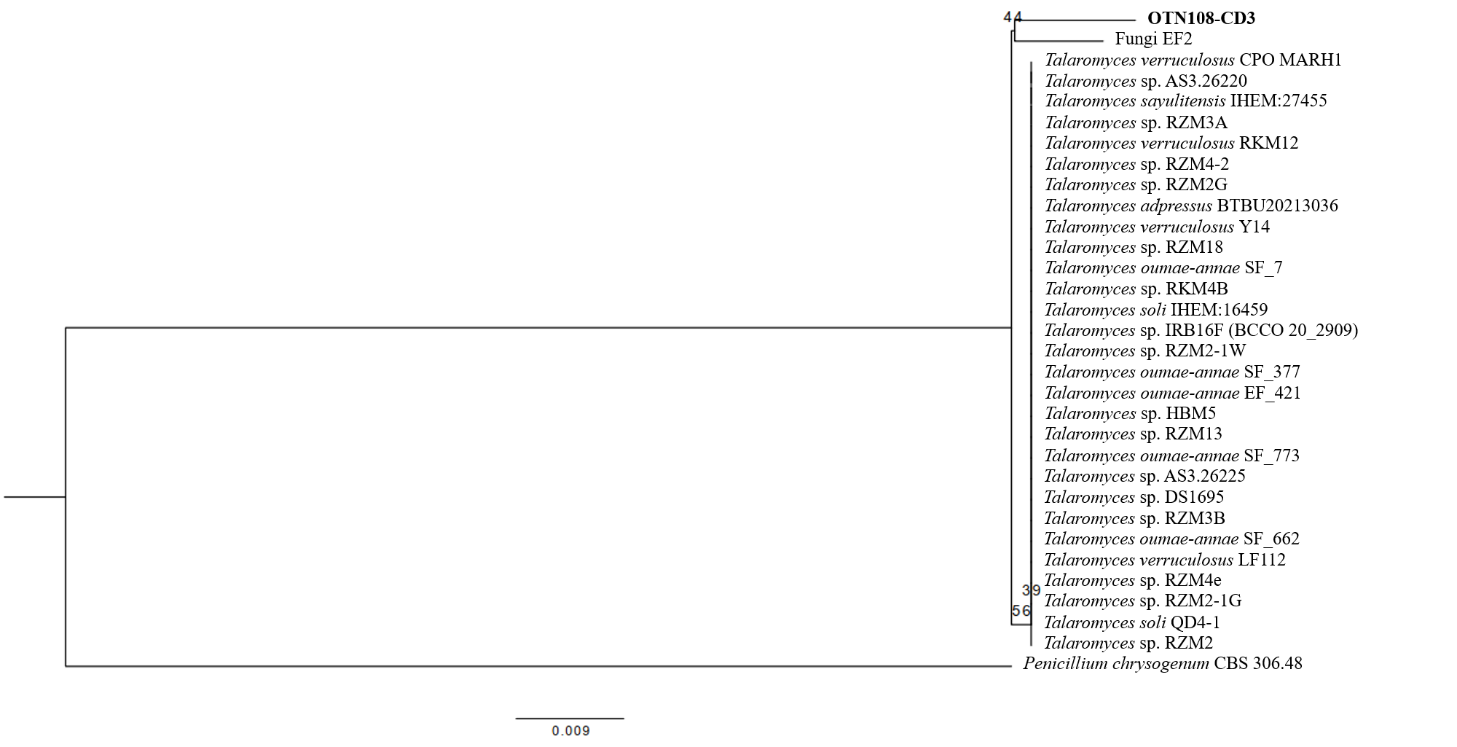


Isolate OTN122-CD1

Substitution model: HKY+F

Outgroup species: *Entomortierella parvispora*


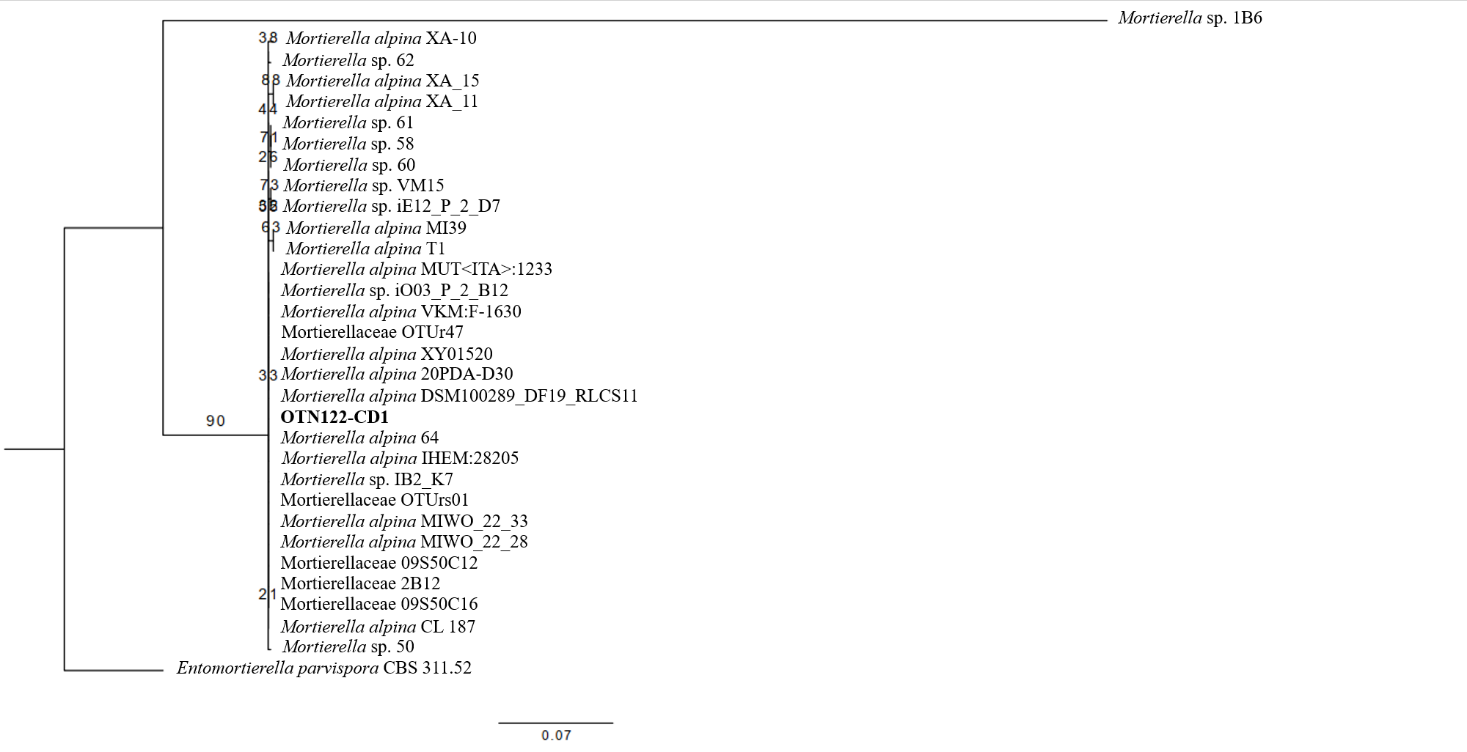


Isolate OTN123-C3

Substitution model: HKY+F

Outgroup species: *Debaryomyces hansenii*


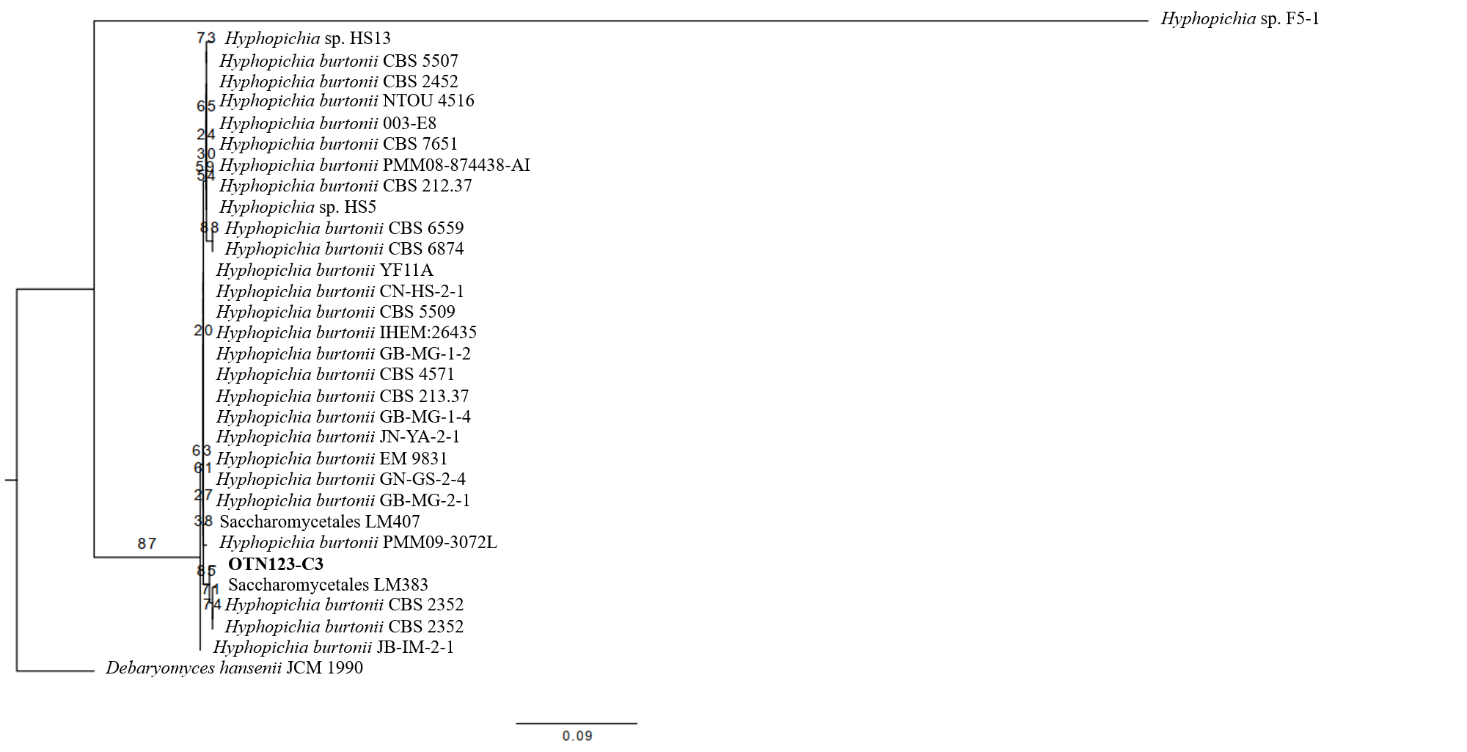


Isolate OTN123-C4

Substitution model: HKY

Outgroup species: *Cephalosporium aphidicola*


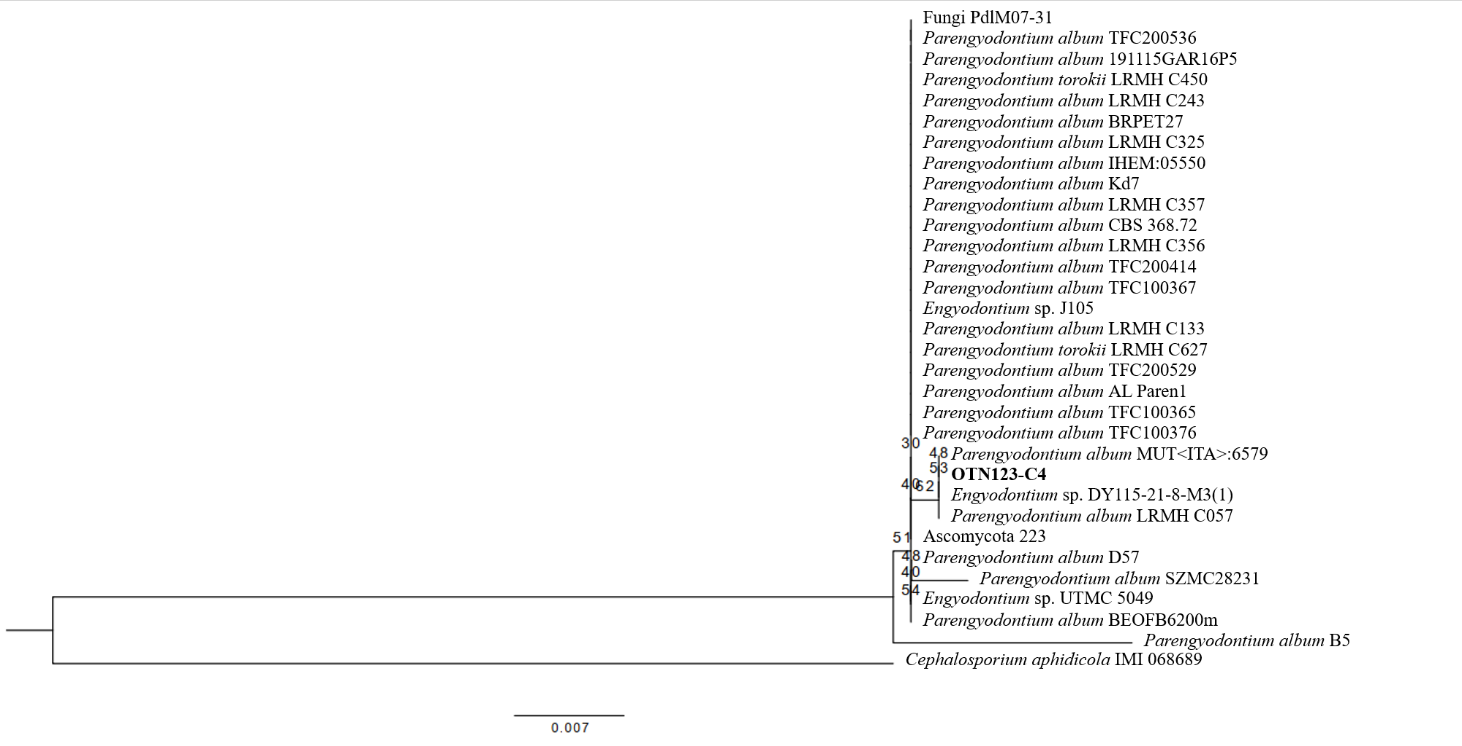


Isolate OTN123-CD4

Substitution model: HKY

Outgroup species: *Sporobolomyces jilinensis*


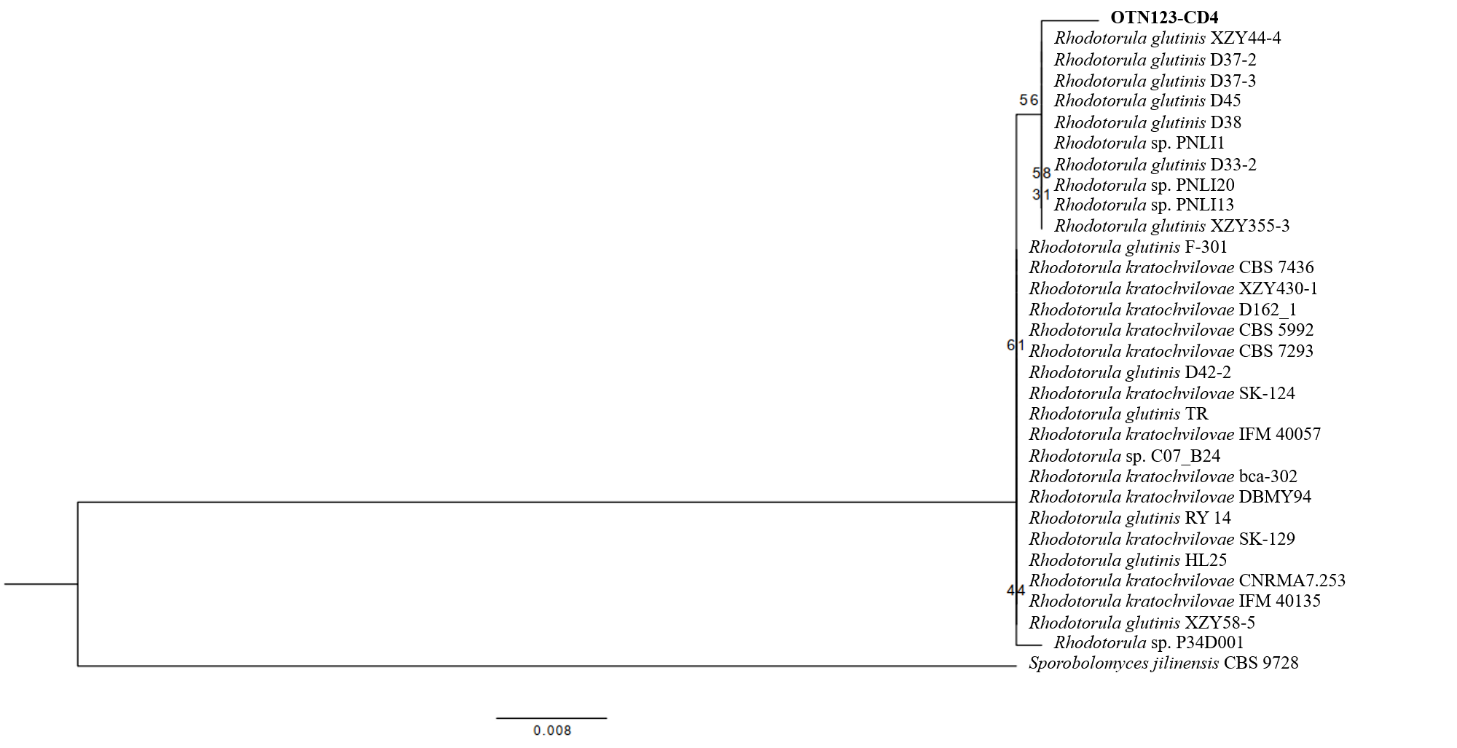


Isolate OTN128-C1

Substitution model: F81

Outgroup species: *Calonectria naviculata*


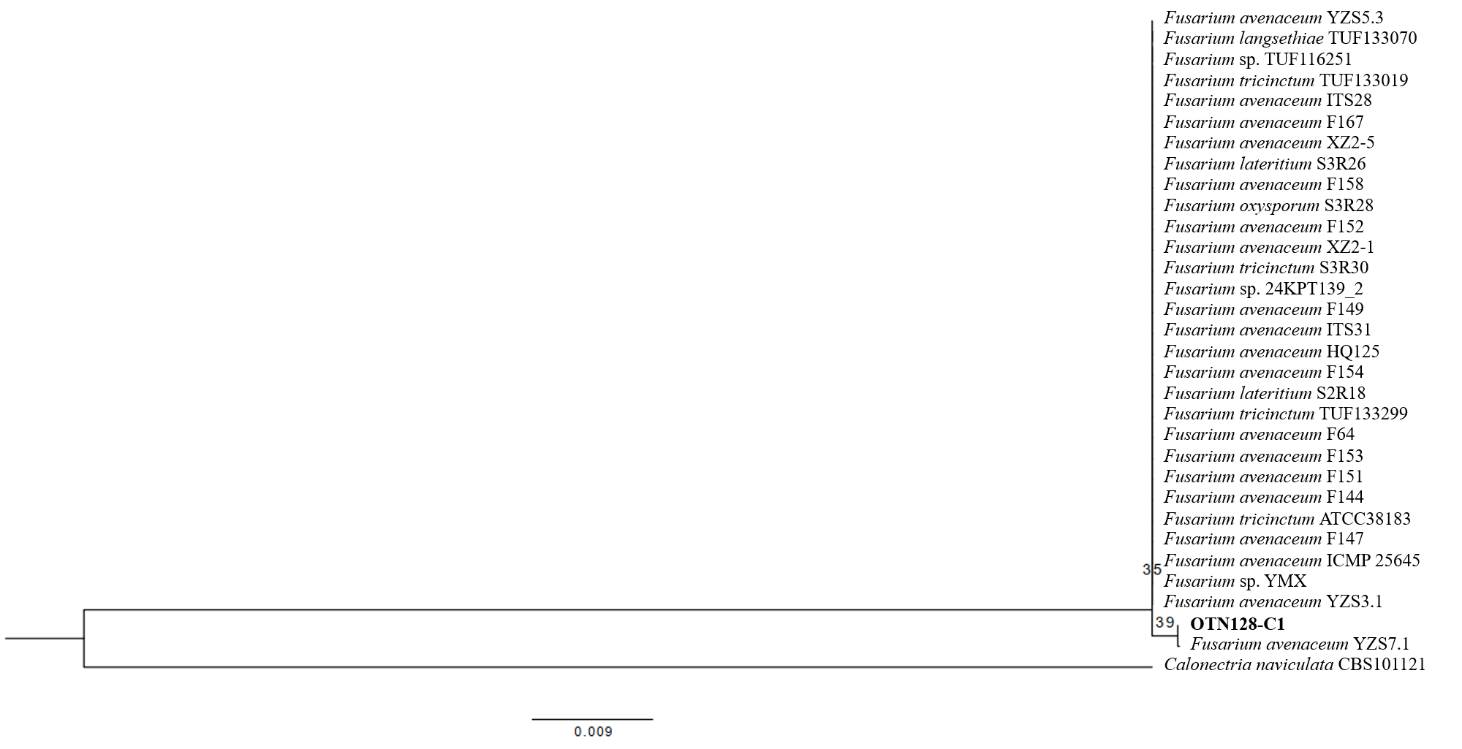


Isolate OTN128-CD4

Substitution model: HKY

Outgroup species: *Toxicocladosporium leucadendri*


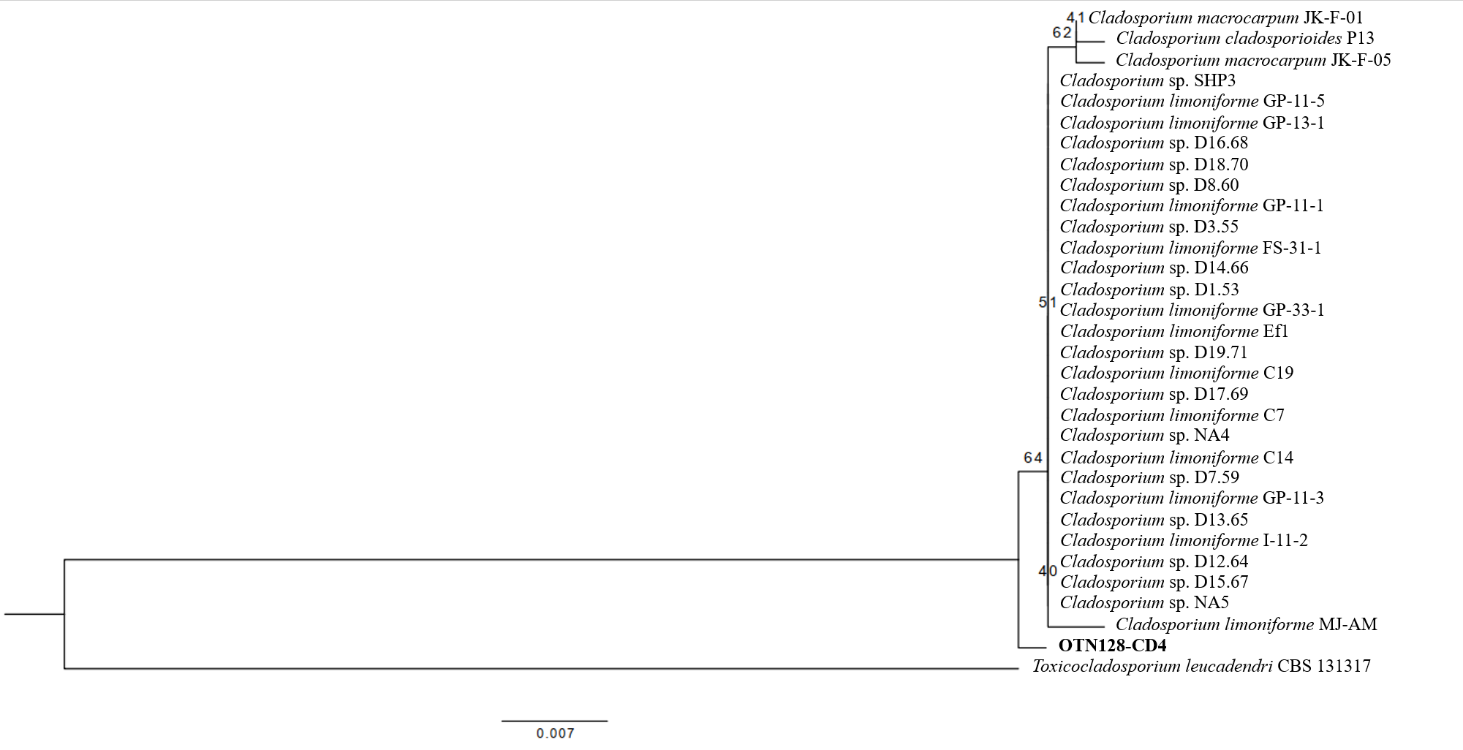


Isolate OTN129-CD5

Substitution model: F81+I

Outgroup species: *Aspergillus inflatus*


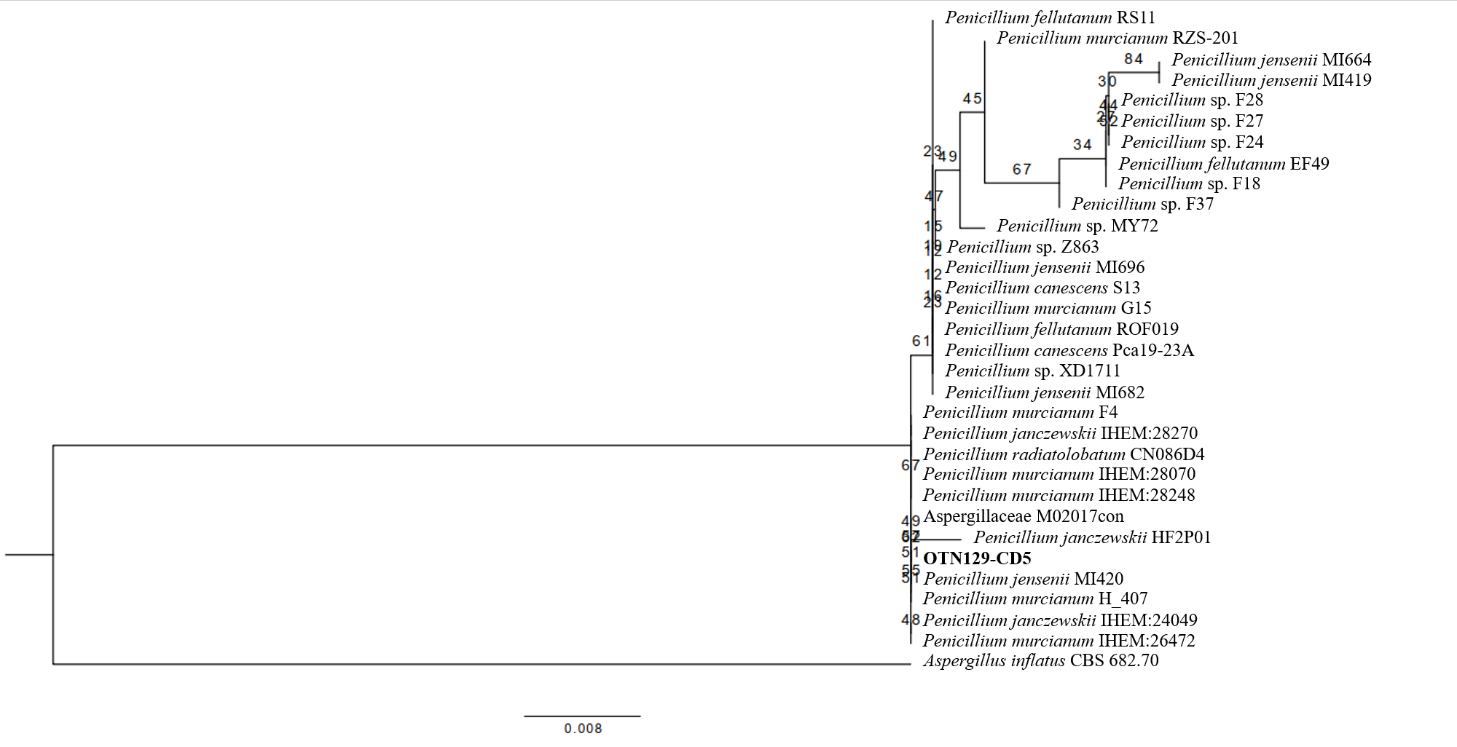


Isolate OTN137-C5

Substitution model: HKY

Outgroup species: *Toxicocladosporium leucadendri*


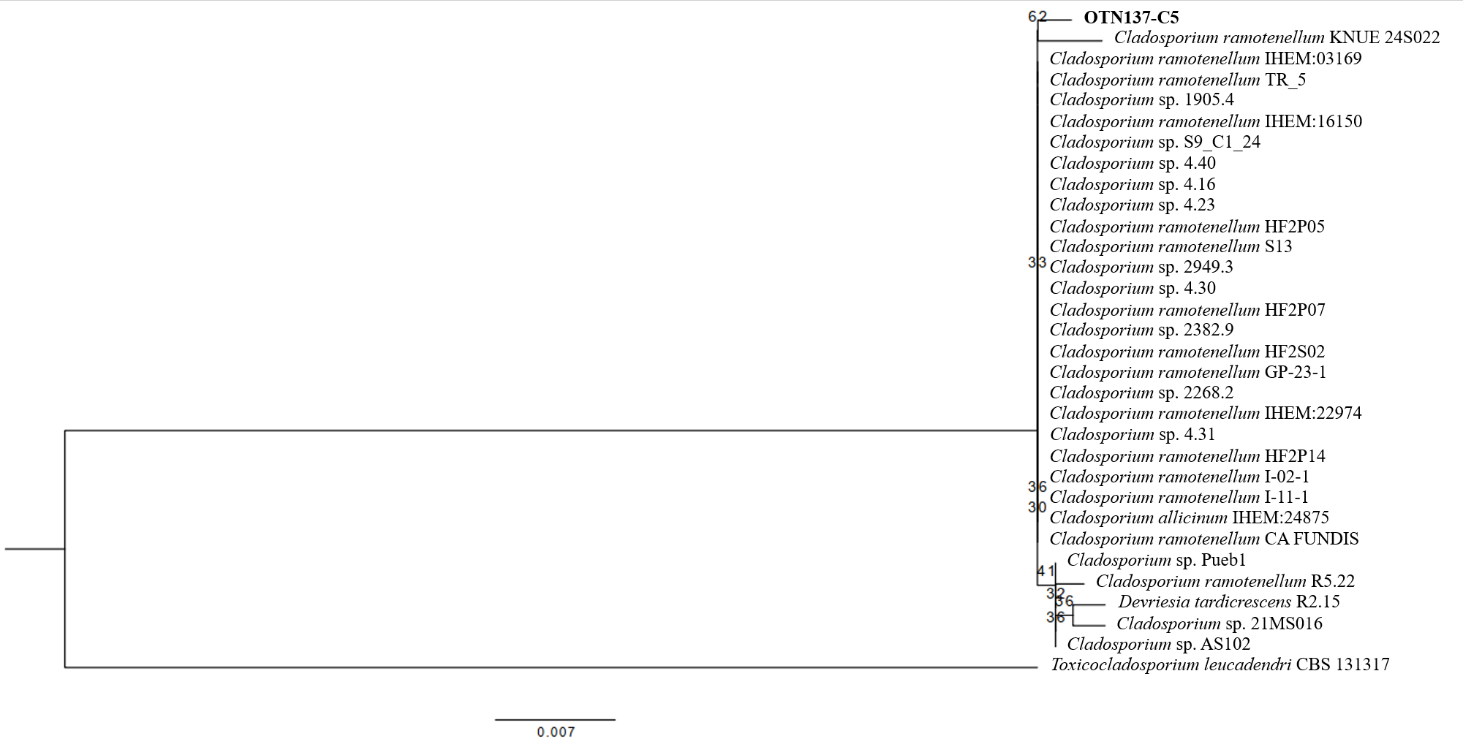


Isolate OTN139-C2

Substitution model: K2P

Outgroup species: *Toxicocladosporium leucadendri*


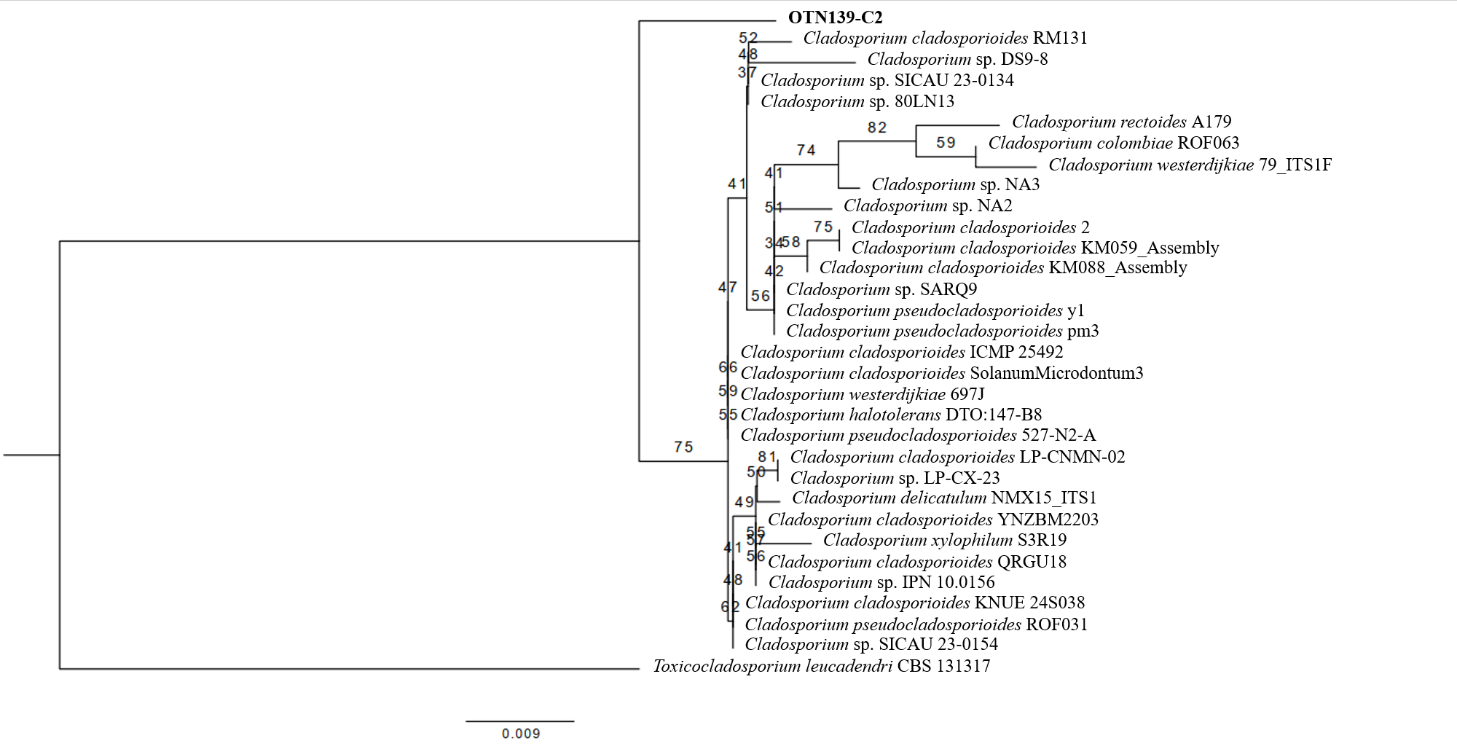


Isolate OTN145-C3

Substitution model: TPM3u+F

Outgroup species: *Entomortierella parvispora*


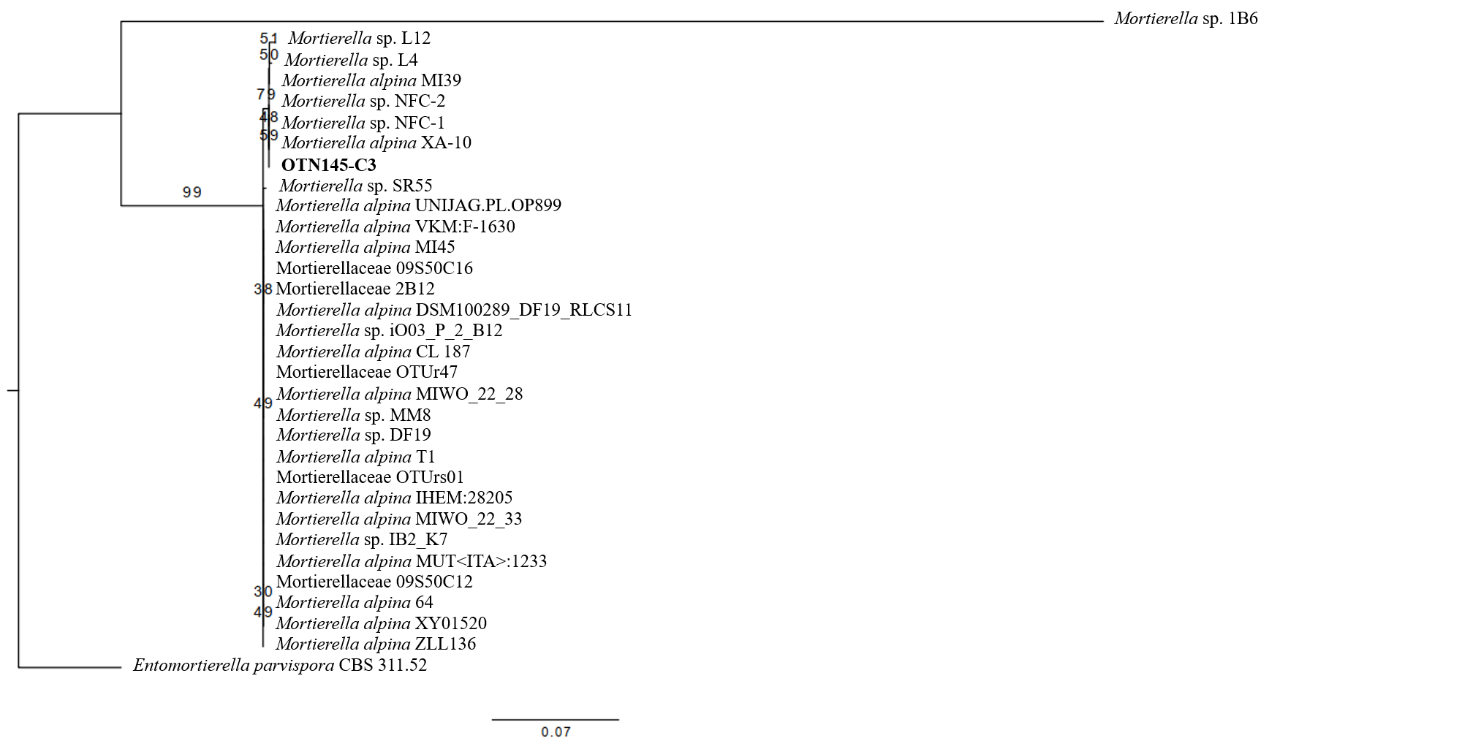


Isolate OTN145-CD5

Substitution model: HKY+F

Outgroup species: *Aspergillus inflatus*


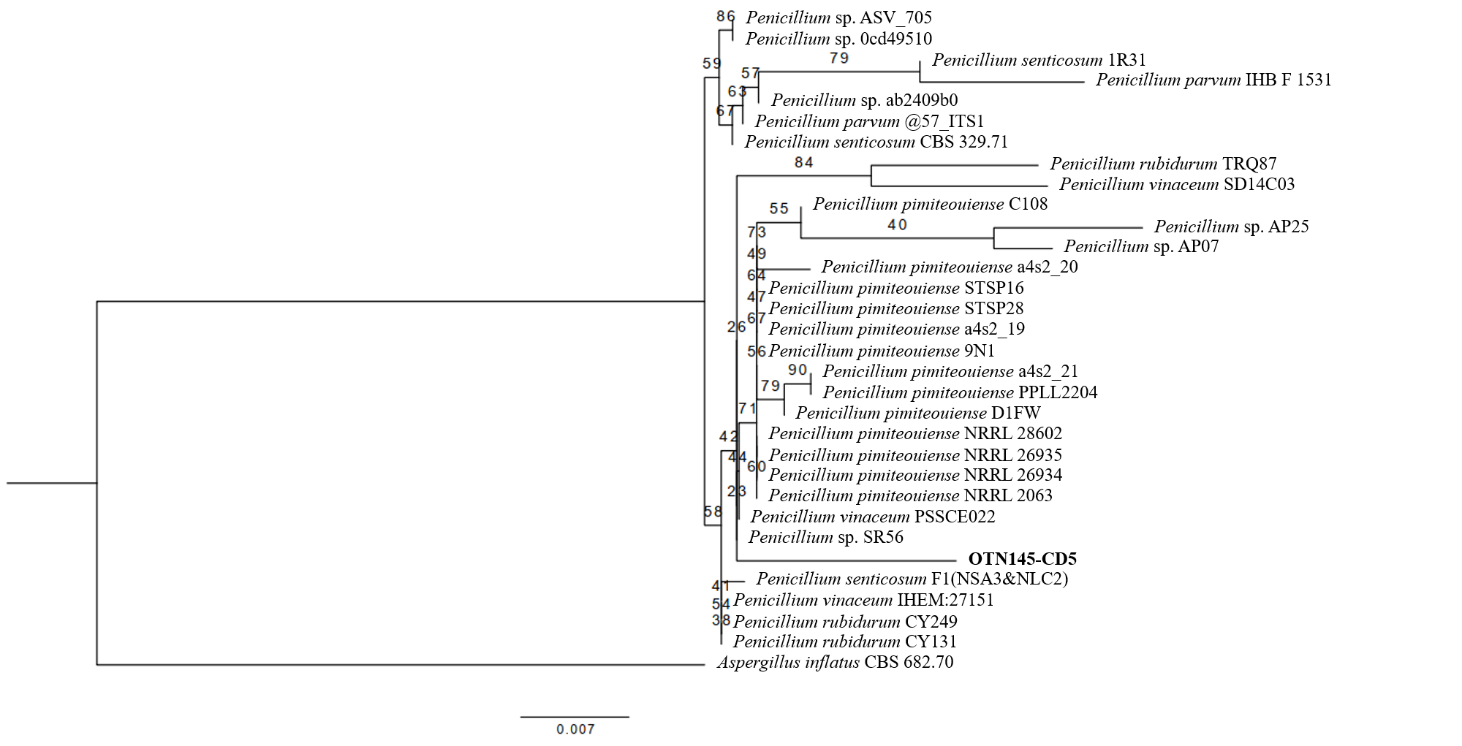


Isolate OTN148-C1

Substitution model: F81

Outgroup species: *Toxicocladosporium leucadendri*


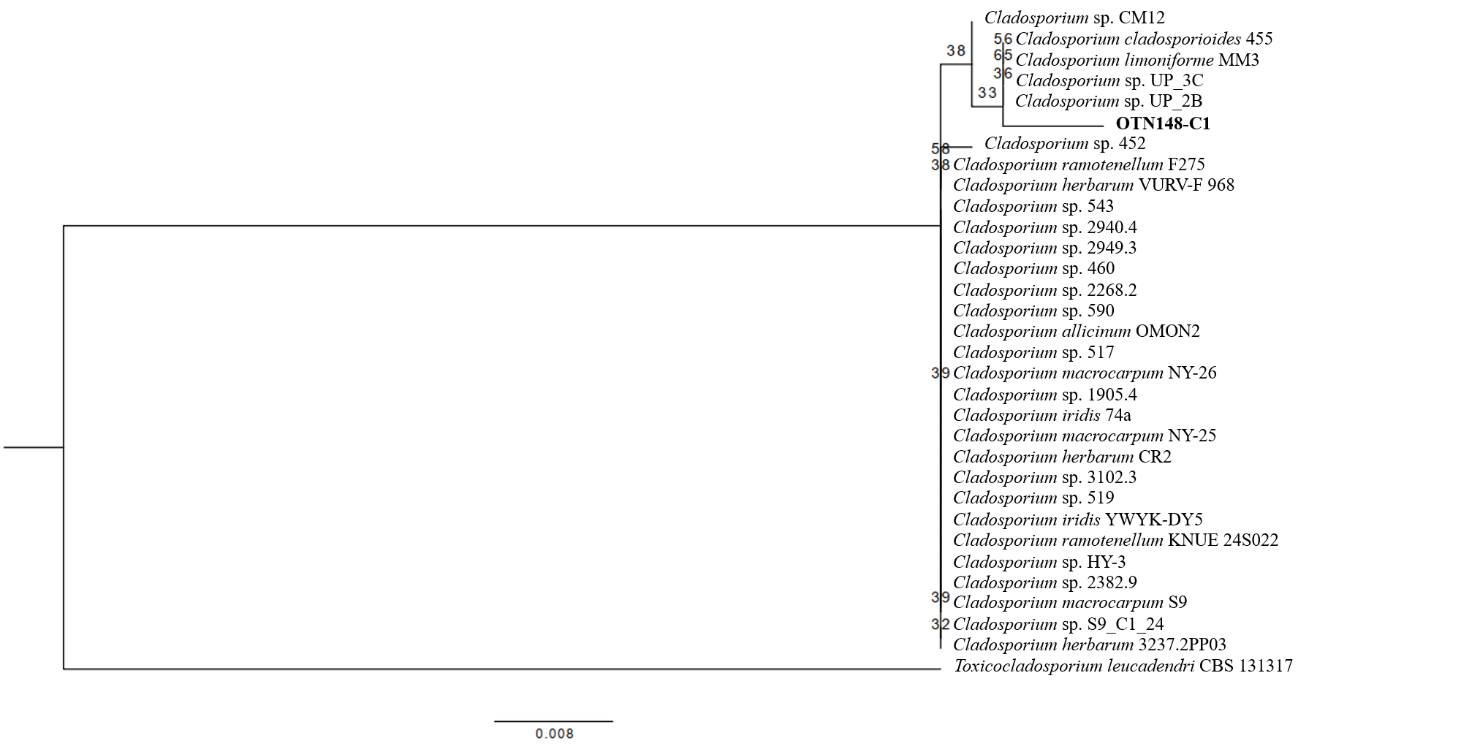


Isolate OTN149-C4

Substitution model: K2P

Outgroup species: *Toxicocladosporium leucadendri*


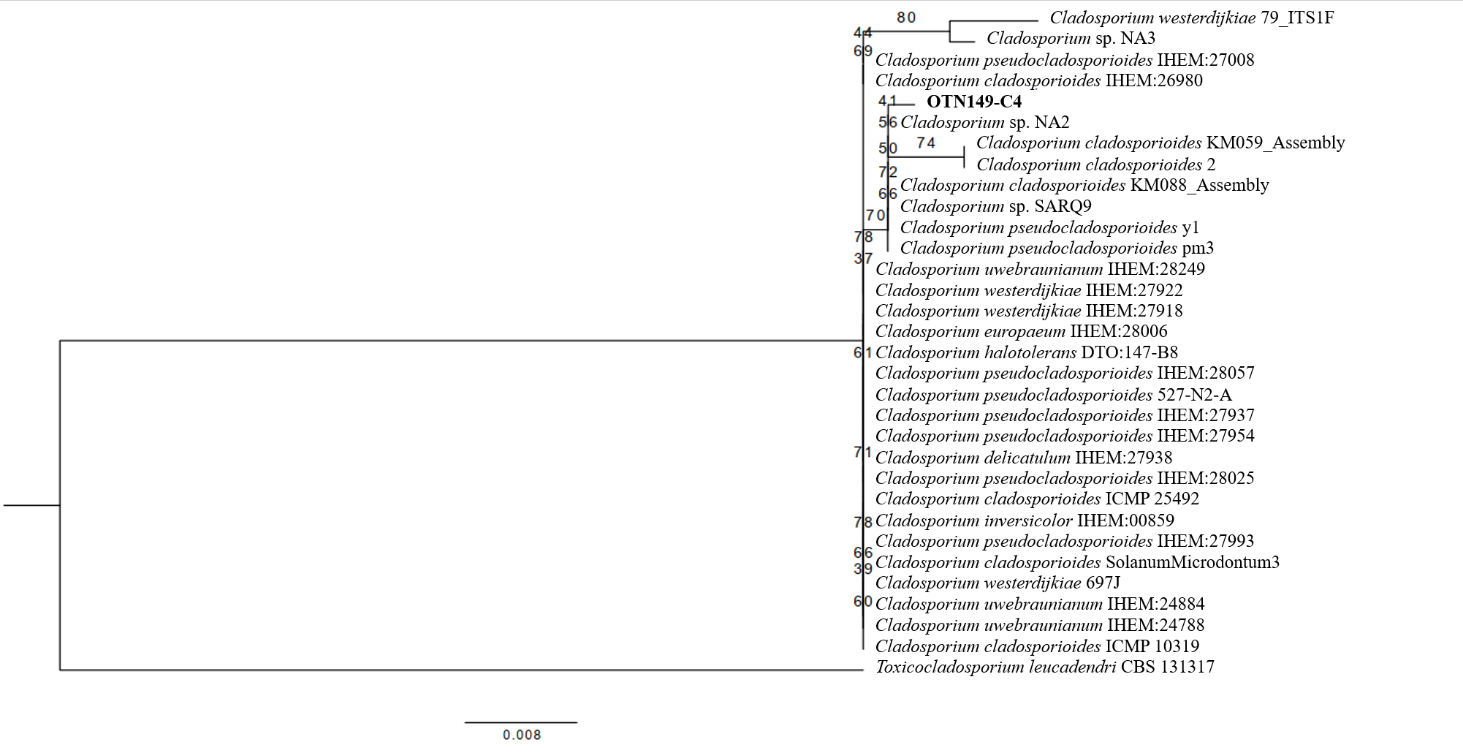


Isolate OTN150-C1

Substitution model: JC

Outgroup species: *Toxicocladosporium leucadendri*


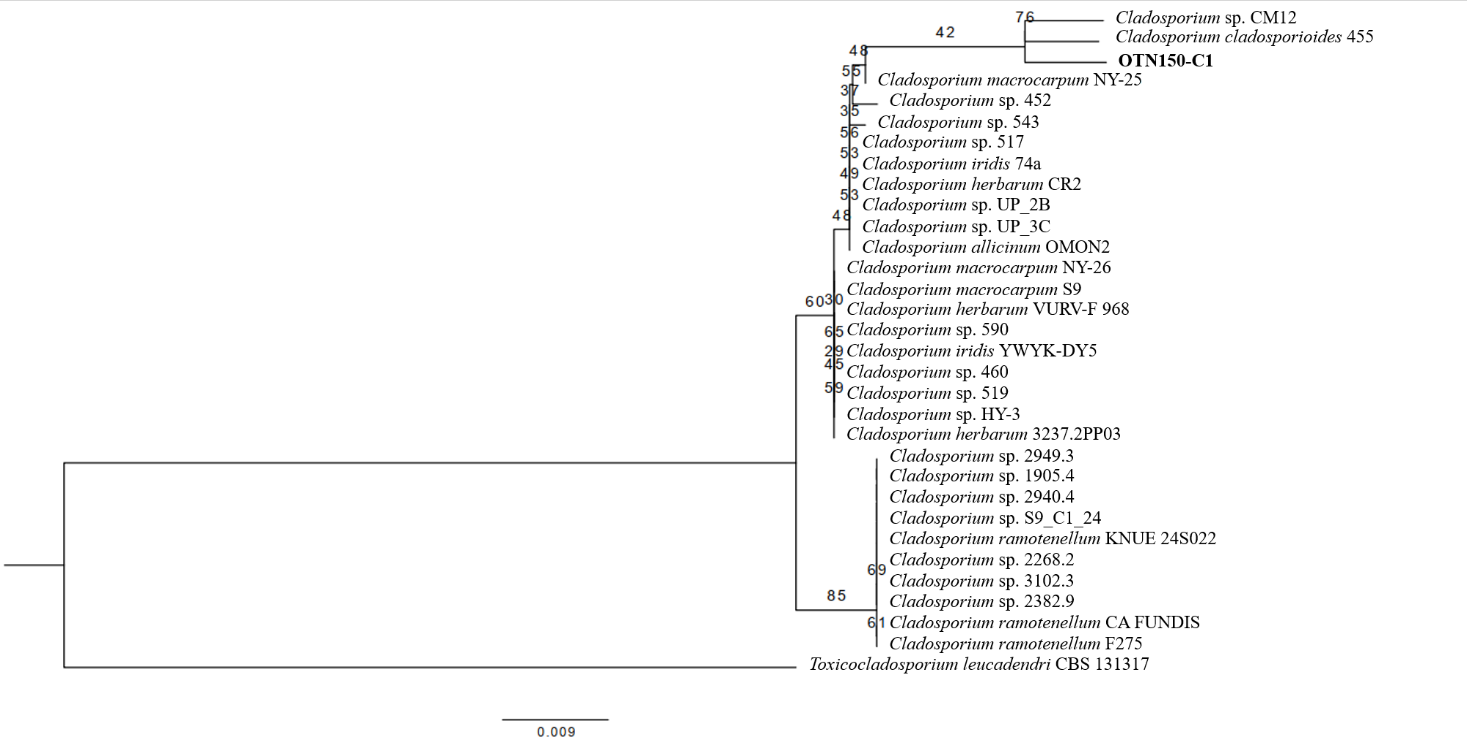


Isolate OTN151-C1

Substitution model: JC

Outgroup species: *Toxicocladosporium leucadendri*


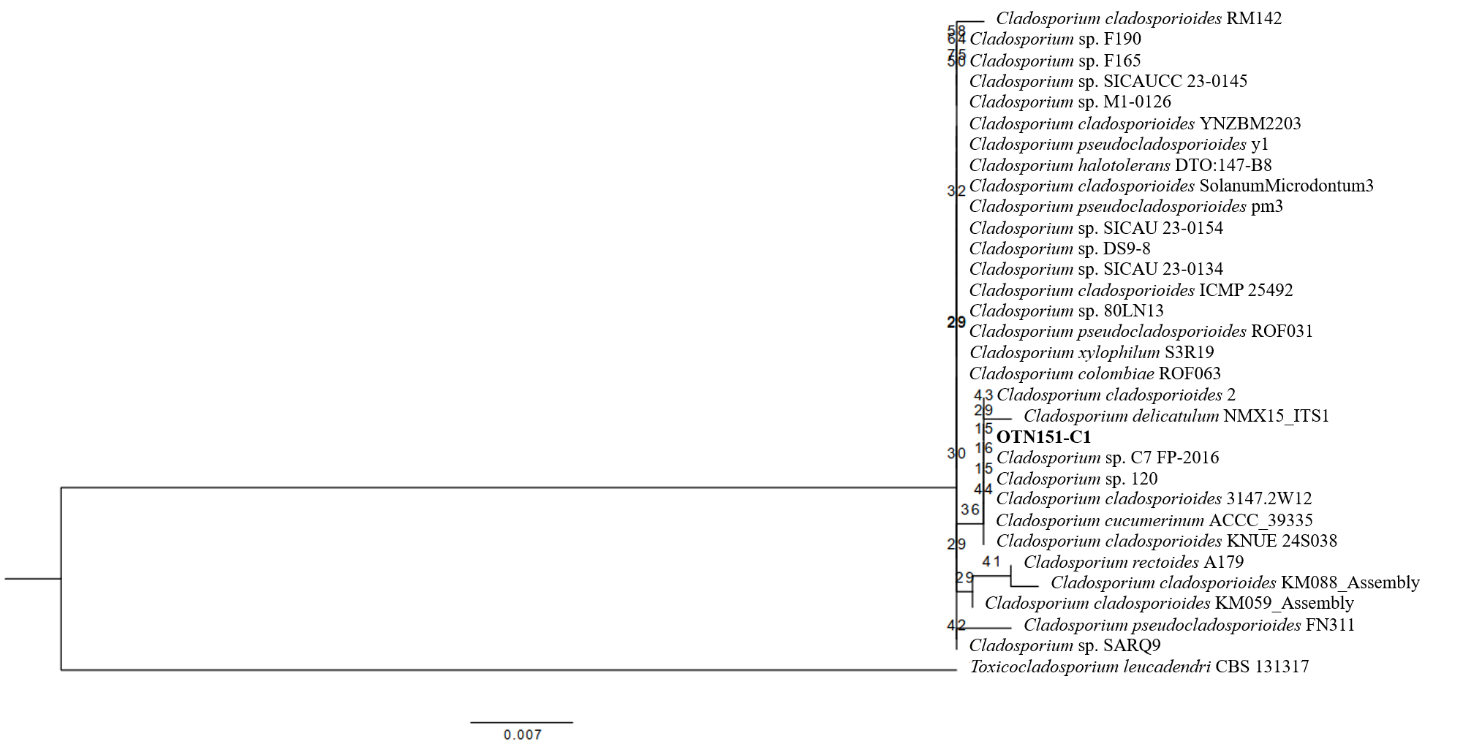


Isolate OTN153-C12

Substitution model: HKY+I

Outgroup species: *Calonectria naviculata*


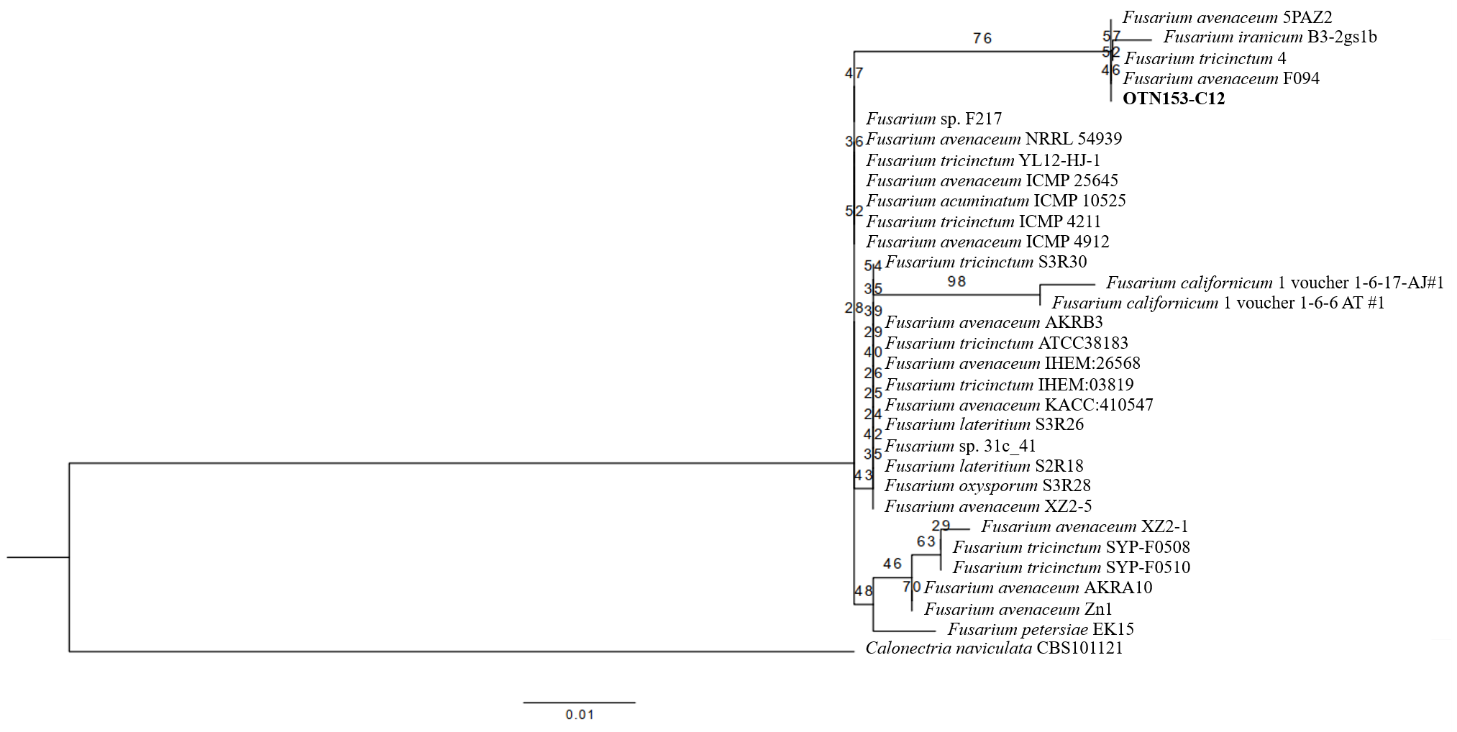


Isolate OTN154-C7

Substitution model: JC

Outgroup species: *Calonectria naviculata*


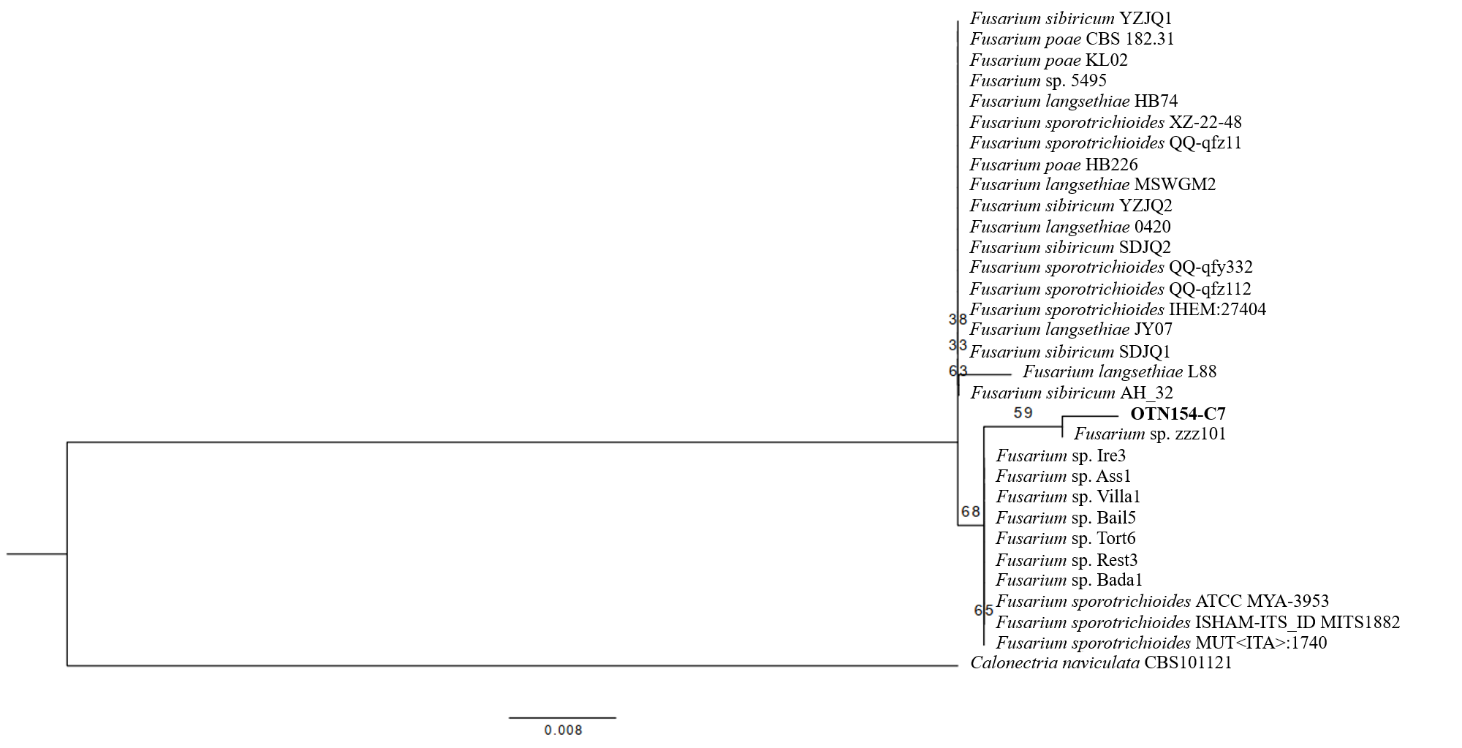


Isolate OTN154-C11

Substitution model: JC

Outgroup species: *Calonectria naviculata*


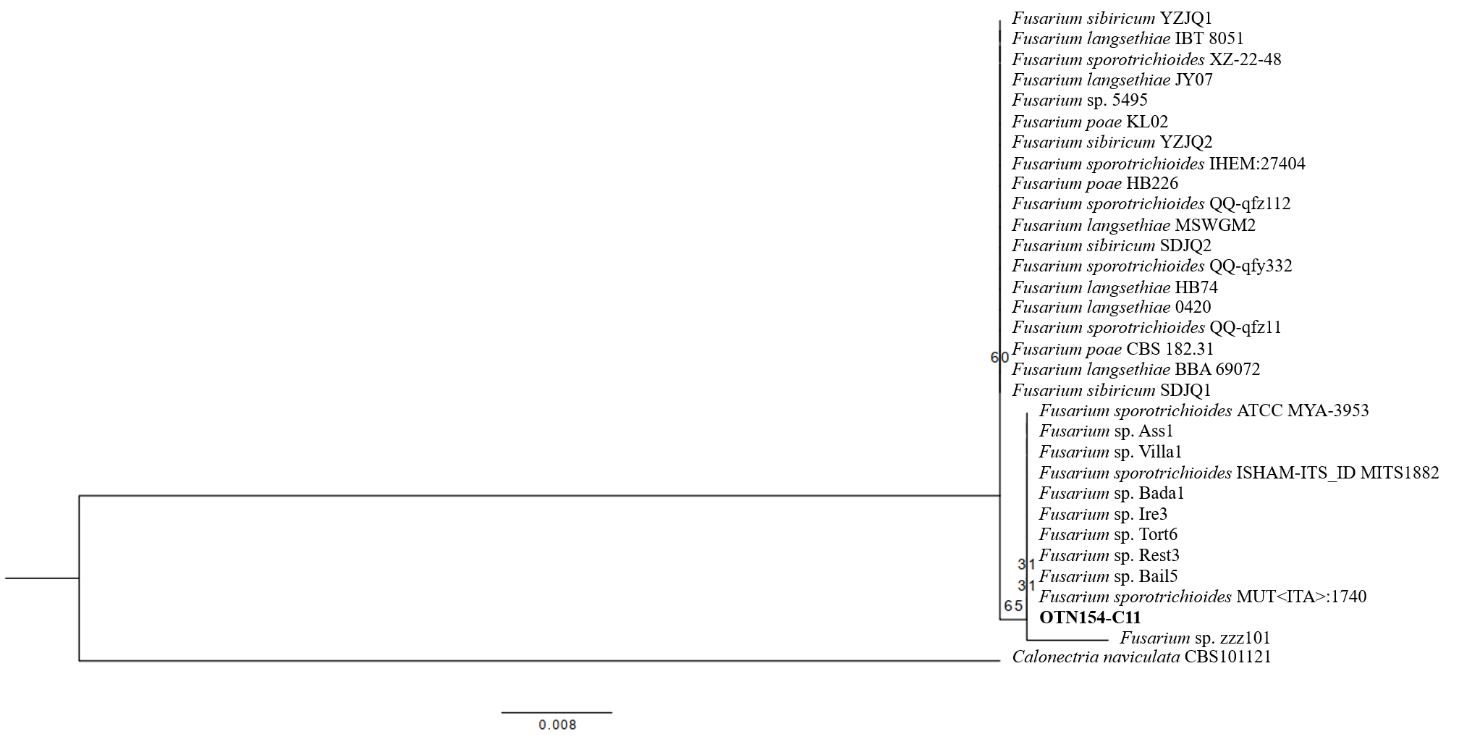


Isolate OTN158-C1

Substitution model: JC+I

Outgroup species: *Toxicocladosporium leucadendri*


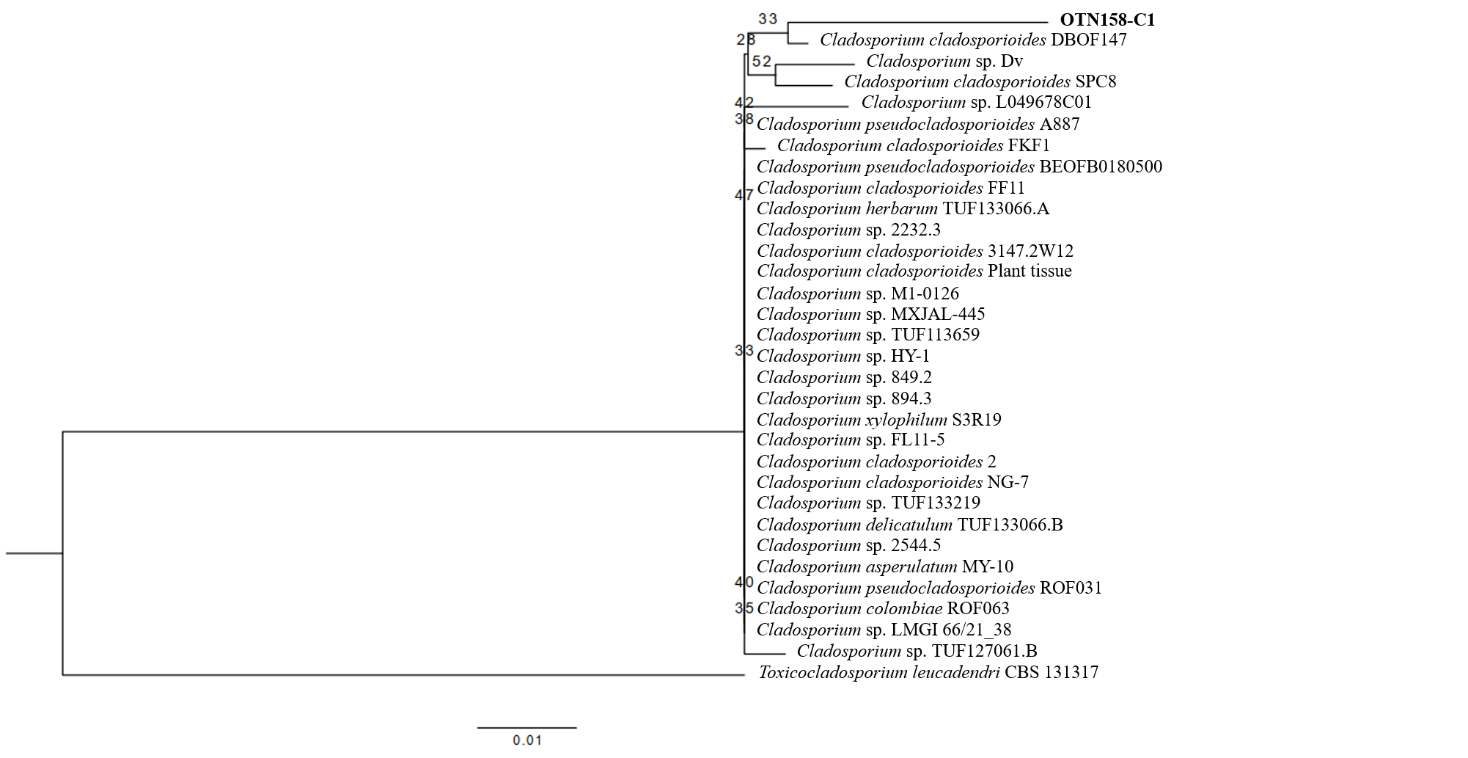


Isolate OTN159-C1

Substitution model: HKY+G4

Outgroup species: *Toxicocladosporium leucadendri*


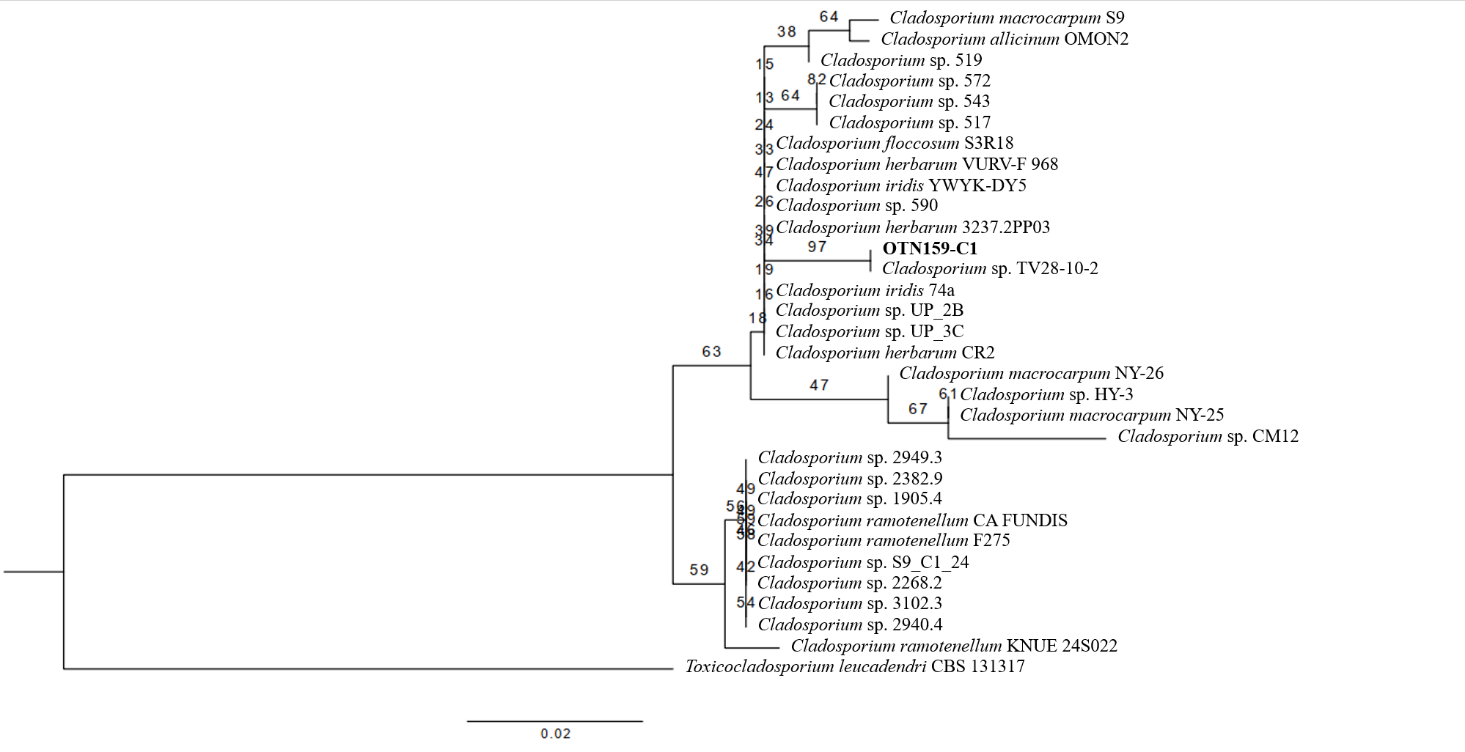


Isolate OTN161-C2

Substitution model: F81

Outgroup species: *Didymella exigua*


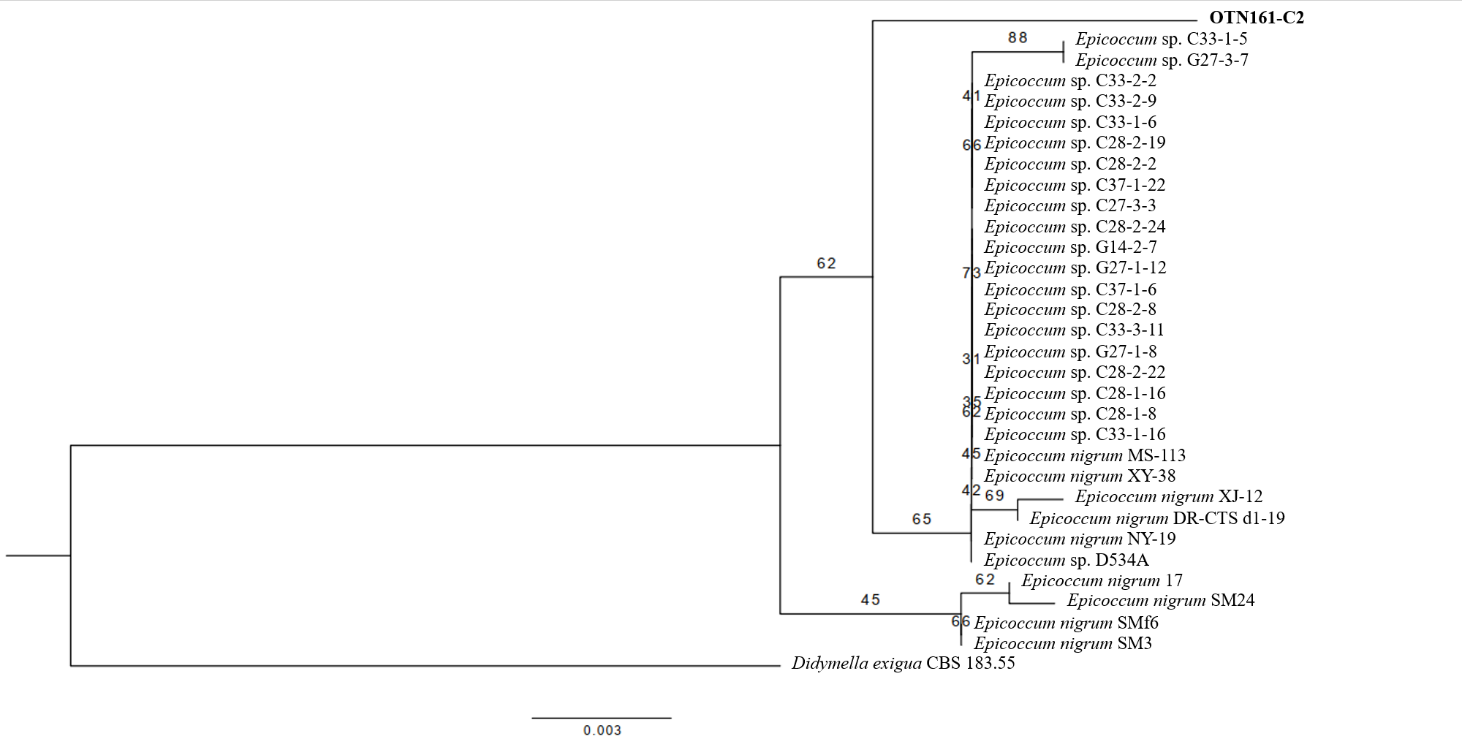


Isolate OTN163-CD1

Substitution model: HKY+F+I

Outgroup species: *Aspergillus inflatus*


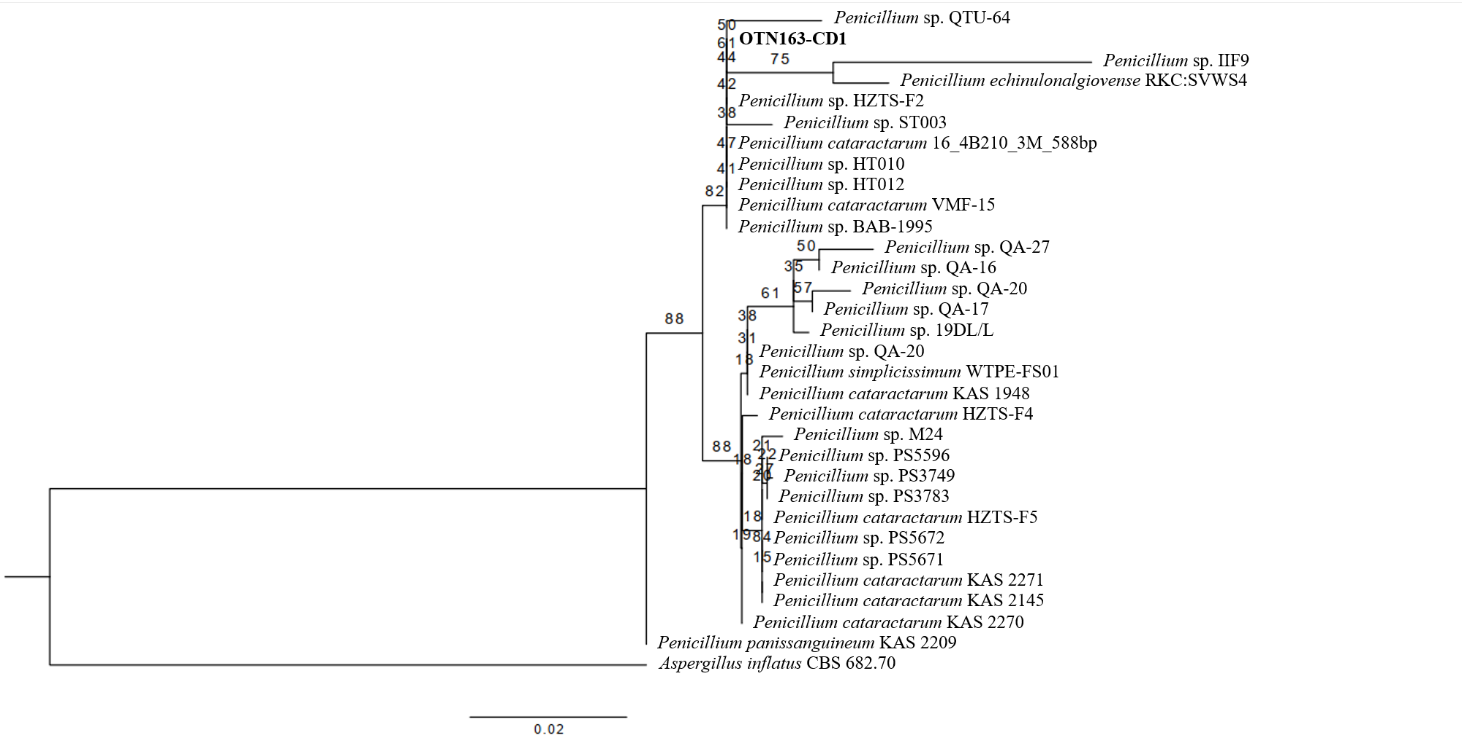


Isolate OTN172-C1

Substitution model: HKY

Outgroup species: *Toxicocladosporium leucadendri*


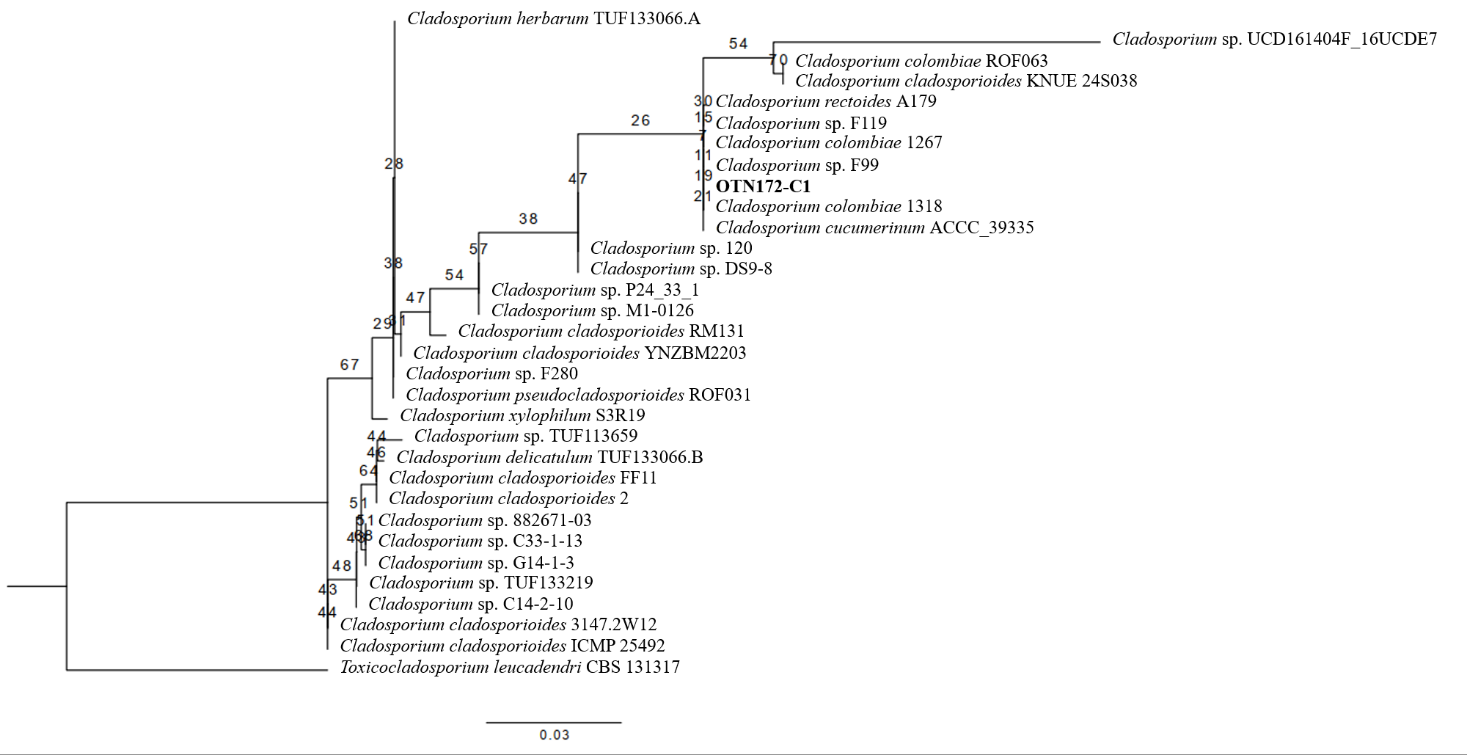


Isolate OTN173-C2

Substitution model: HKY

Outgroup species: *Toxicocladosporium leucadendri*


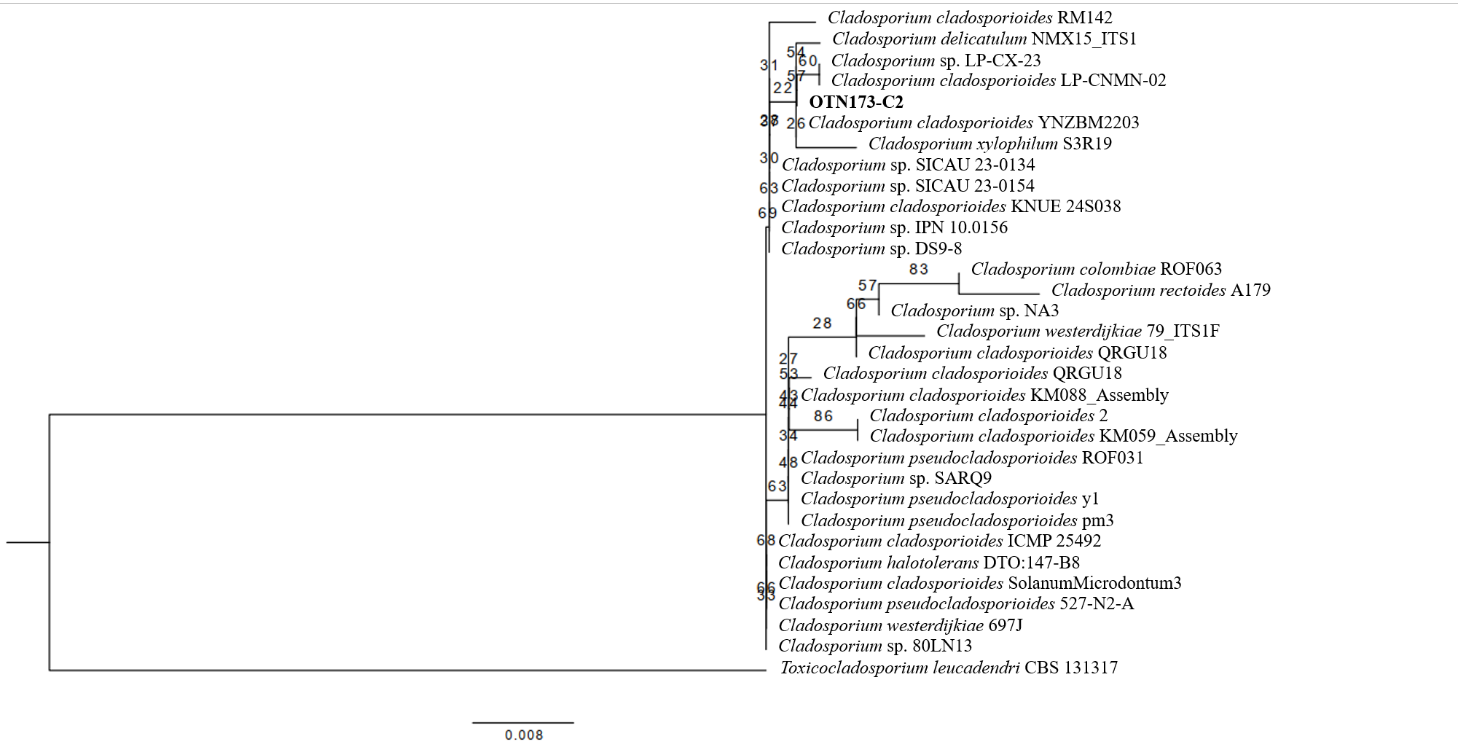


Isolate OTN177-C2

Substitution model: HKY

Outgroup species: *Toxicocladosporium leucadendri*


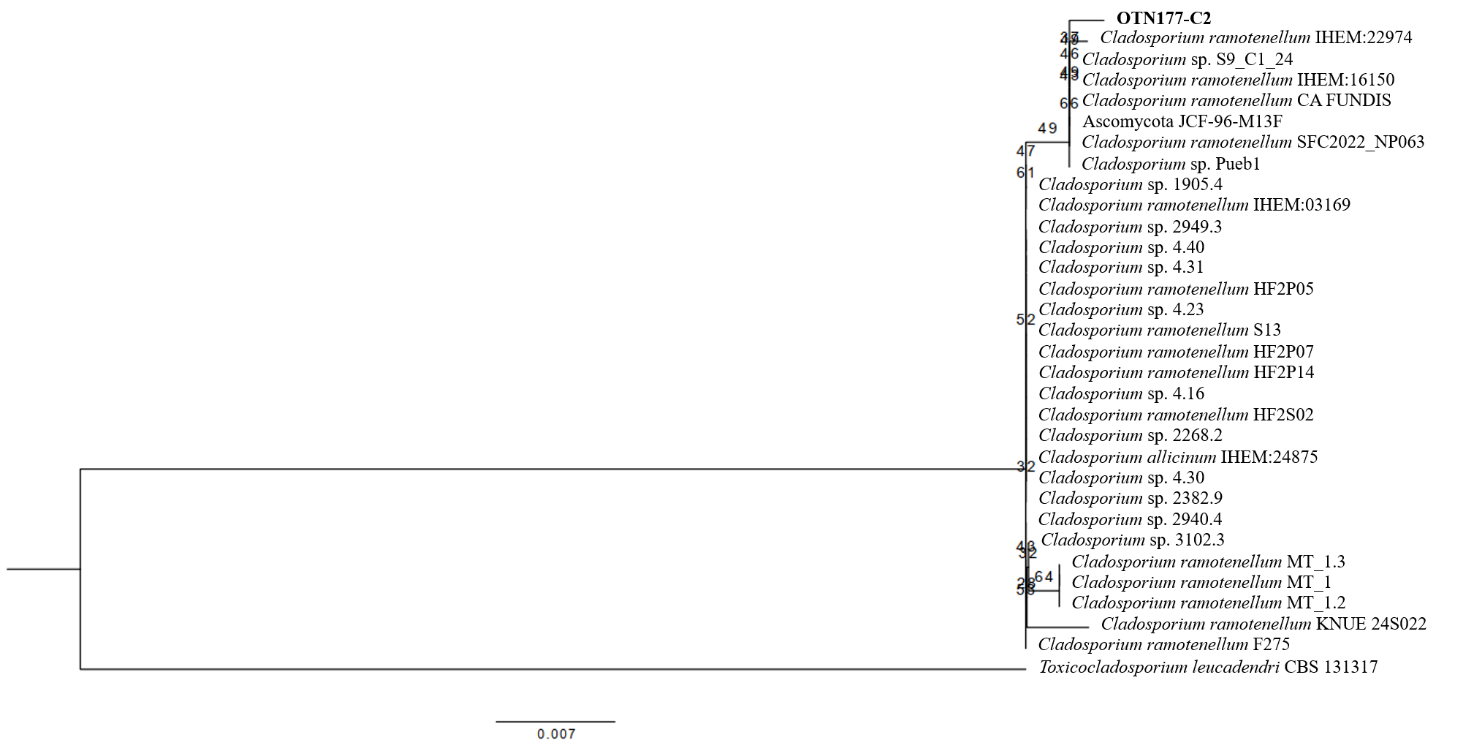


Isolate OTN178-CD2

Substitution model: K2P

Outgroup species: *Aspergillus inflatus*


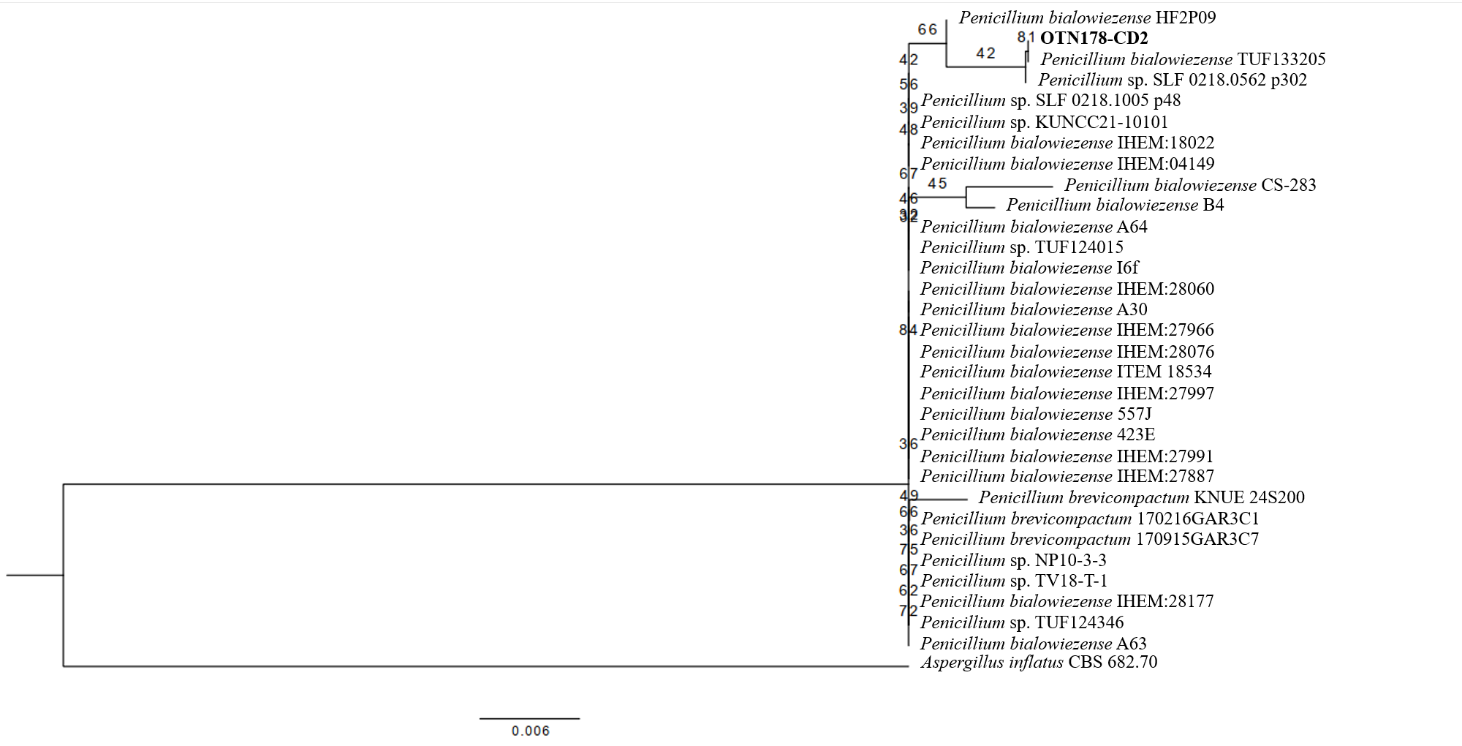


Isolate OTN178-CD3

Substitution model: TN

Outgroup species: *Penicillium chrysogenum*


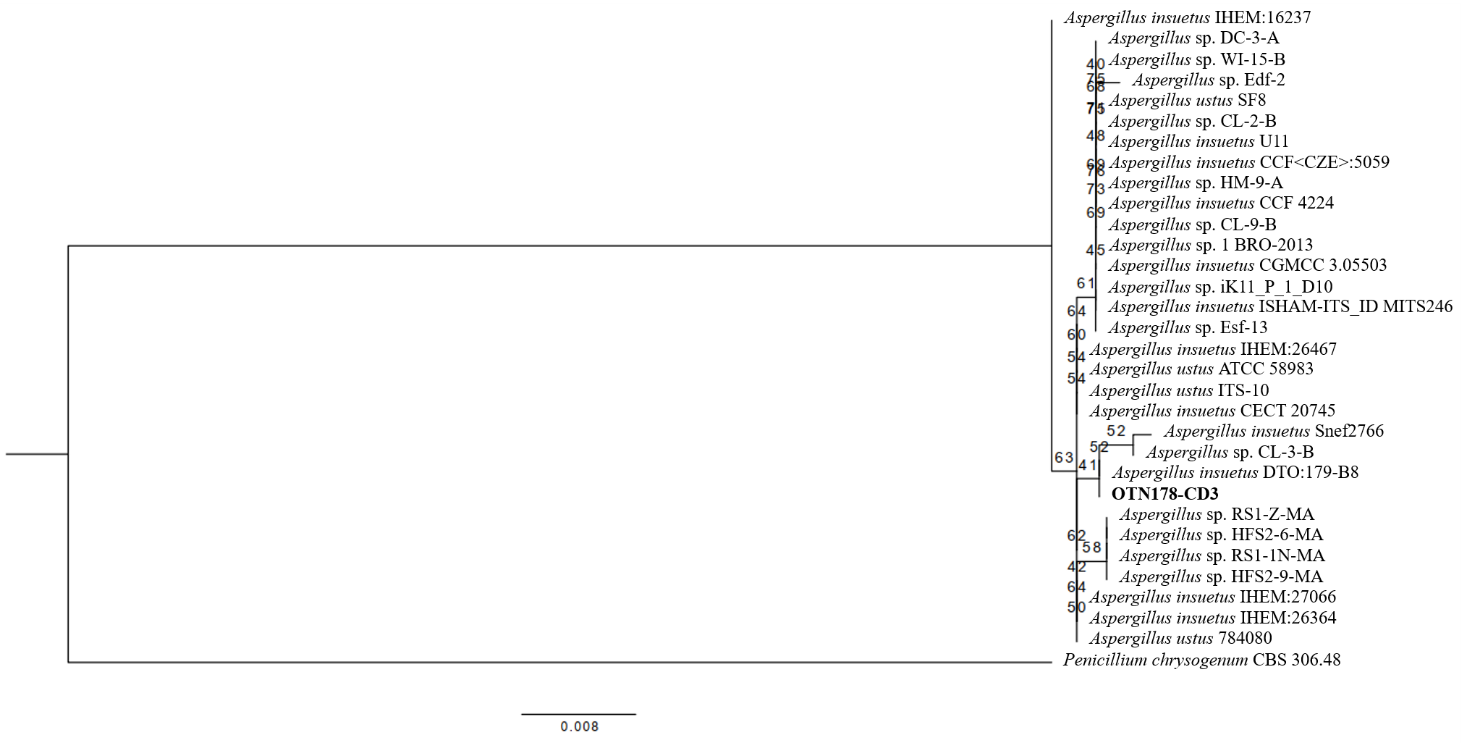


Isolate OTN179-CD3

Substitution model: K2P

Outgroup species: *Toxicocladosporium leucadendri*


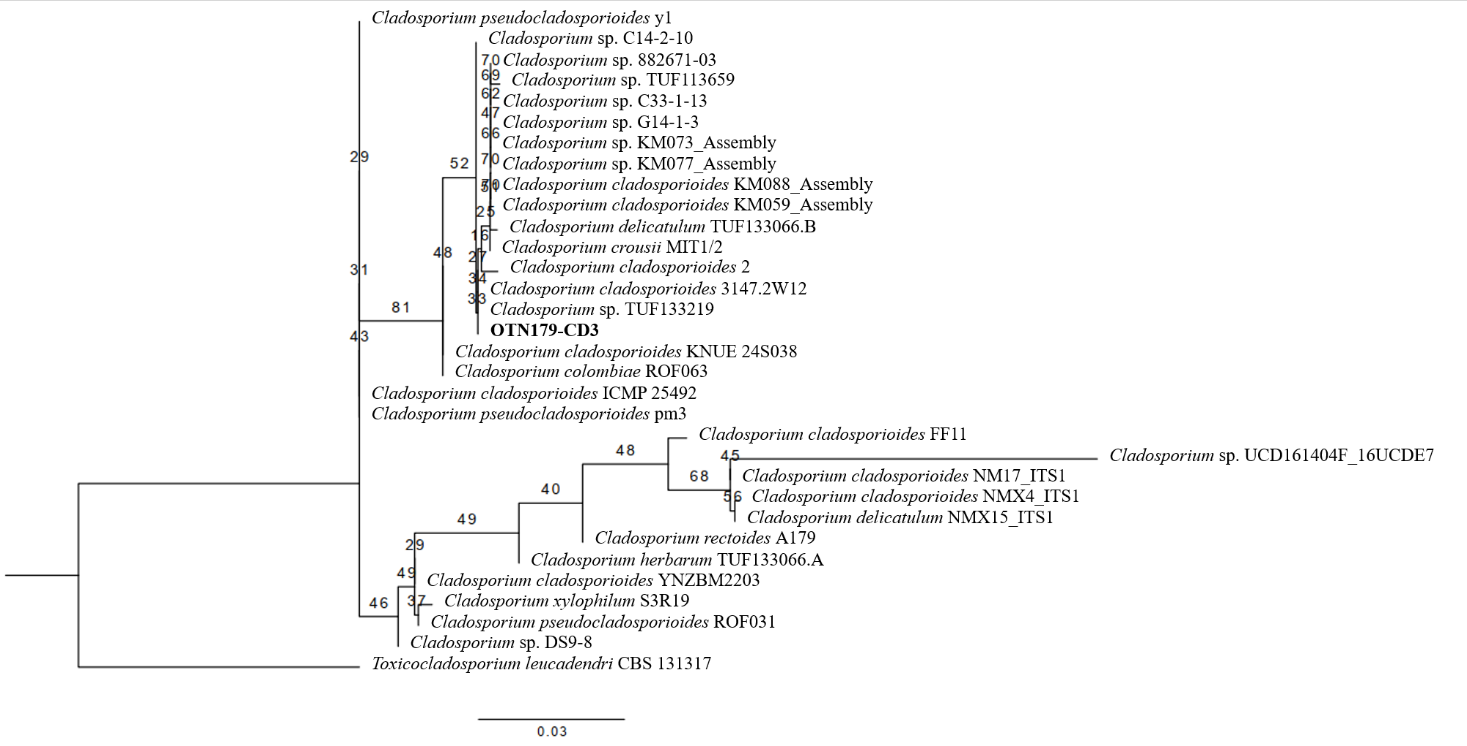


Isolate OTN181-CD1

Substitution model: HKY+F

Outgroup species: *Hirsutella thompsonii*


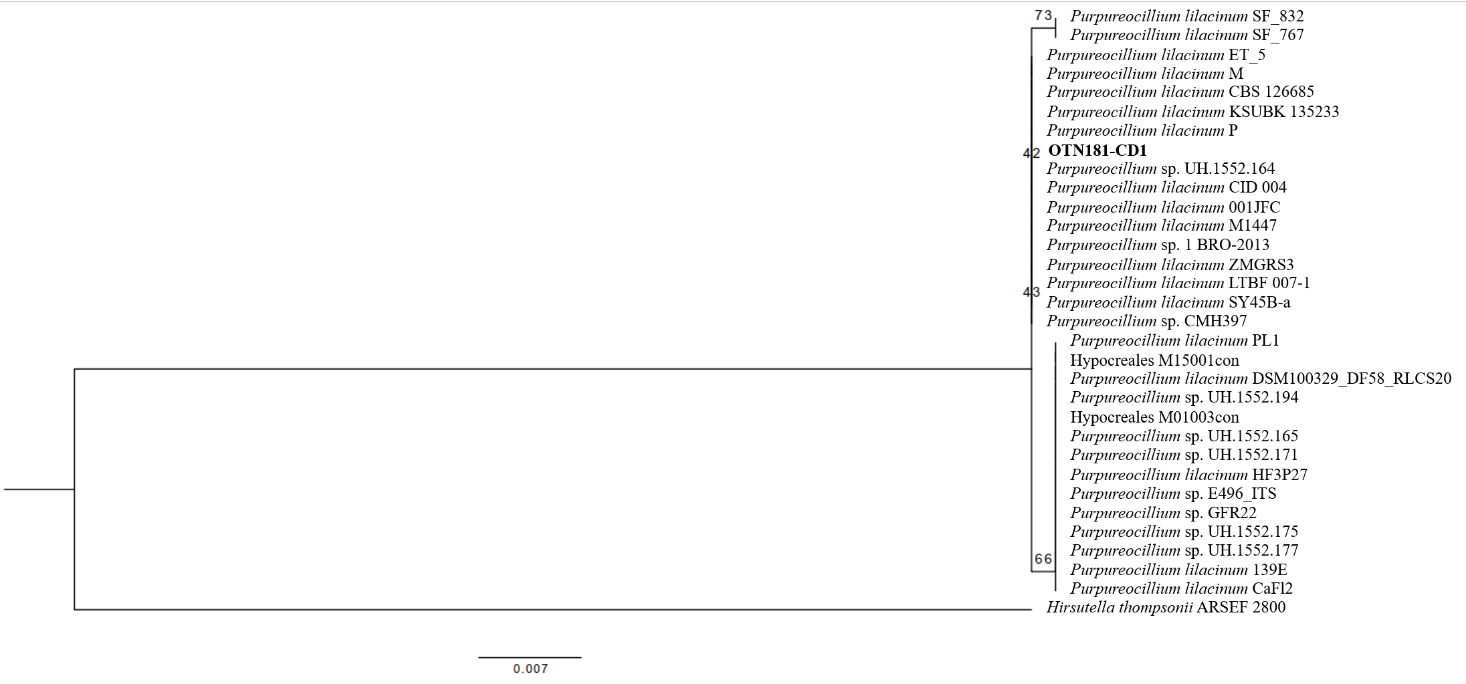


Isolate OTN182-C1

Substitution model: F81-I

Outgroup species: *Nigrospora* sp.


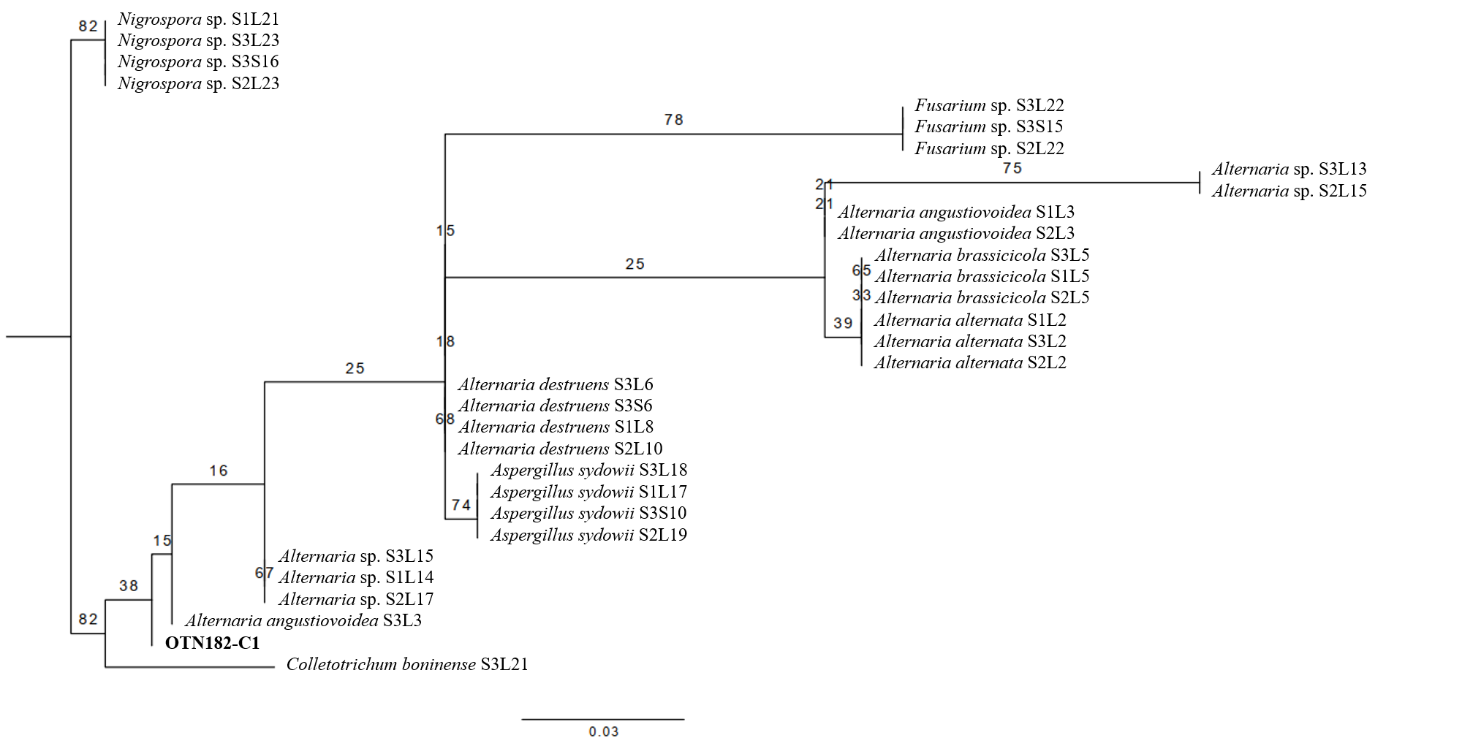


Isolate OTN185-C6

Substitution model: TN

Outgroup species: *Penicillium chrysogenum*


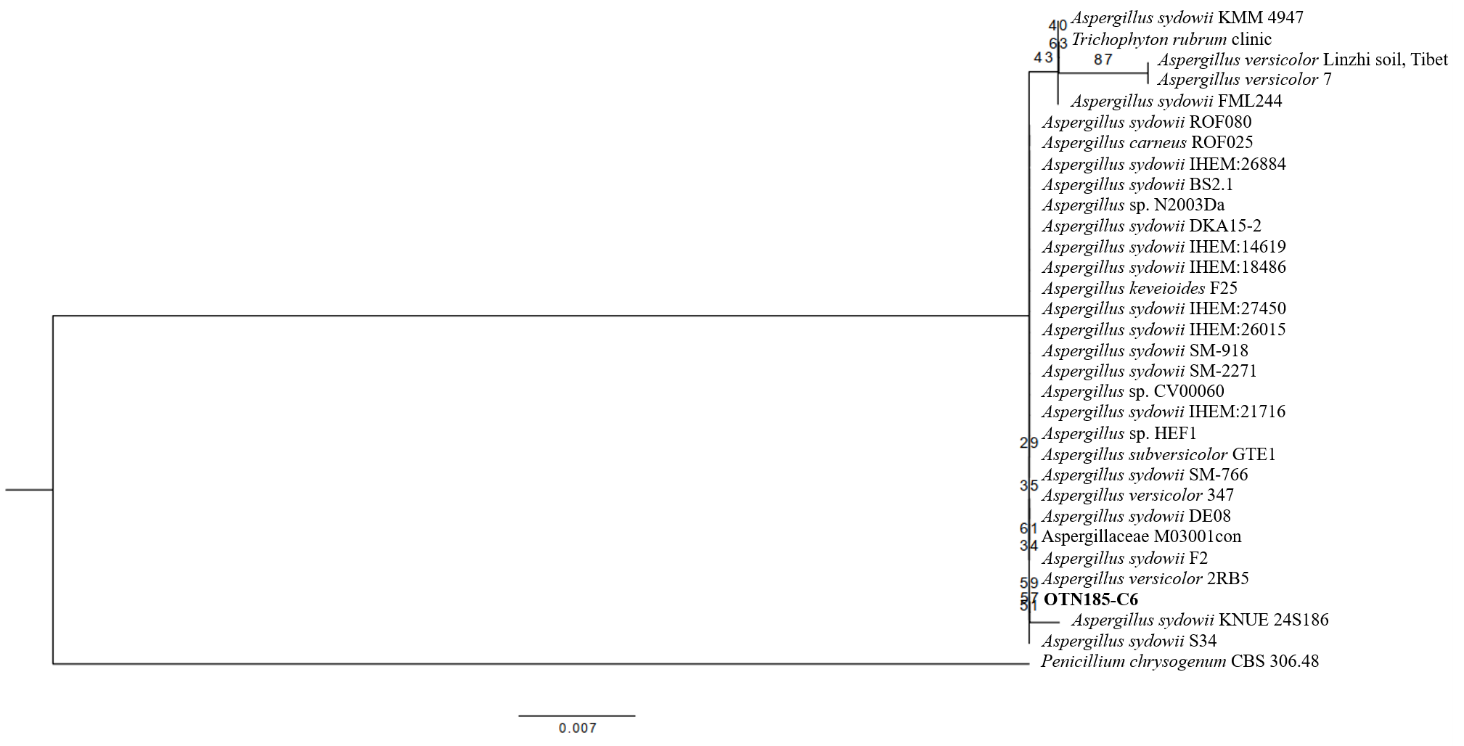


Isolate OTN185-CD5

Substitution model: HKY

Outgroup species: *Toxicocladosporium leucadendri*


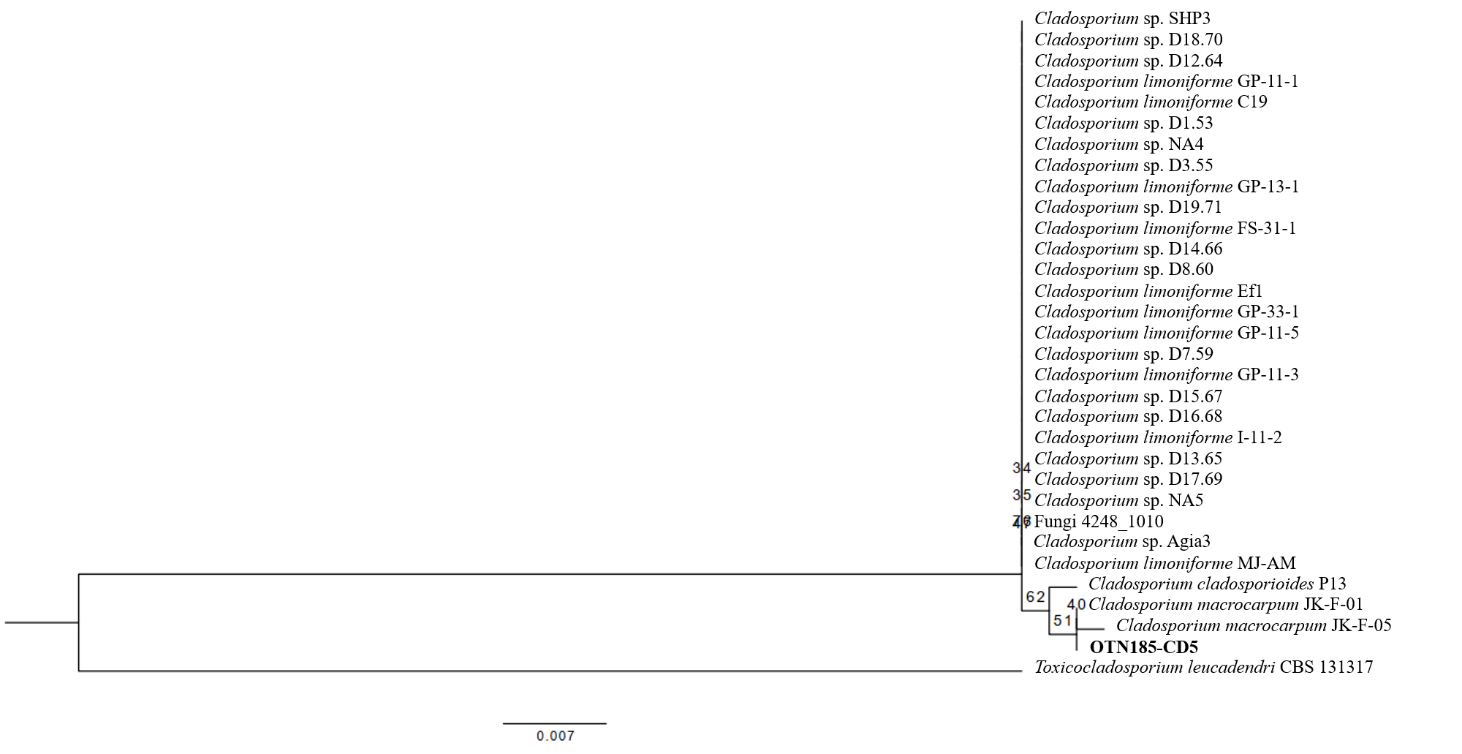


Isolate BS1-C3

Substitution model: HKY+F+G4

Outgroup species: *Debaryomyces hansenii*


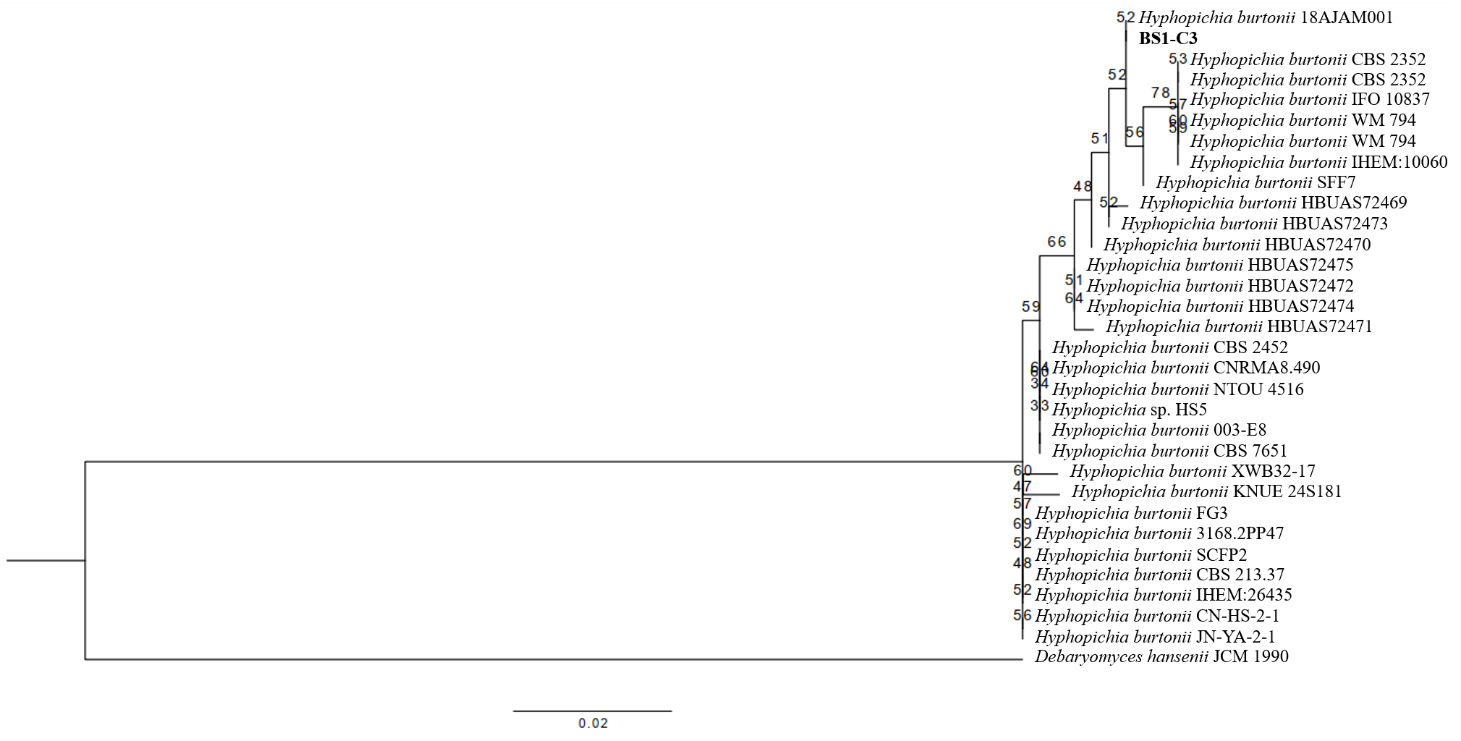


Isolate BS1-C4

Substitution model: TPM2u+F

Outgroup species: *Aspergillus inflatus*


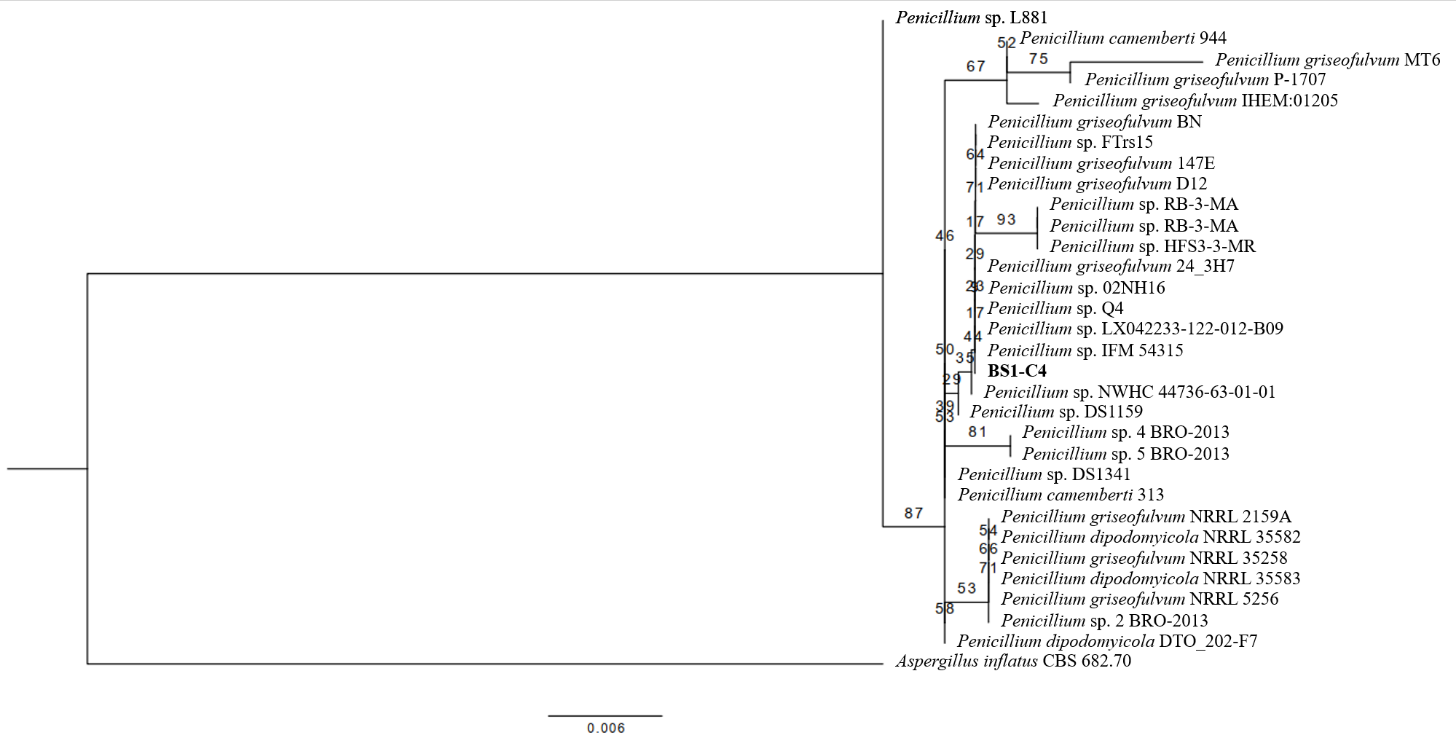


Isolate BS1-C6

Substitution model: K2P

Outgroup species: *Toxicocladosporium leucadendri*


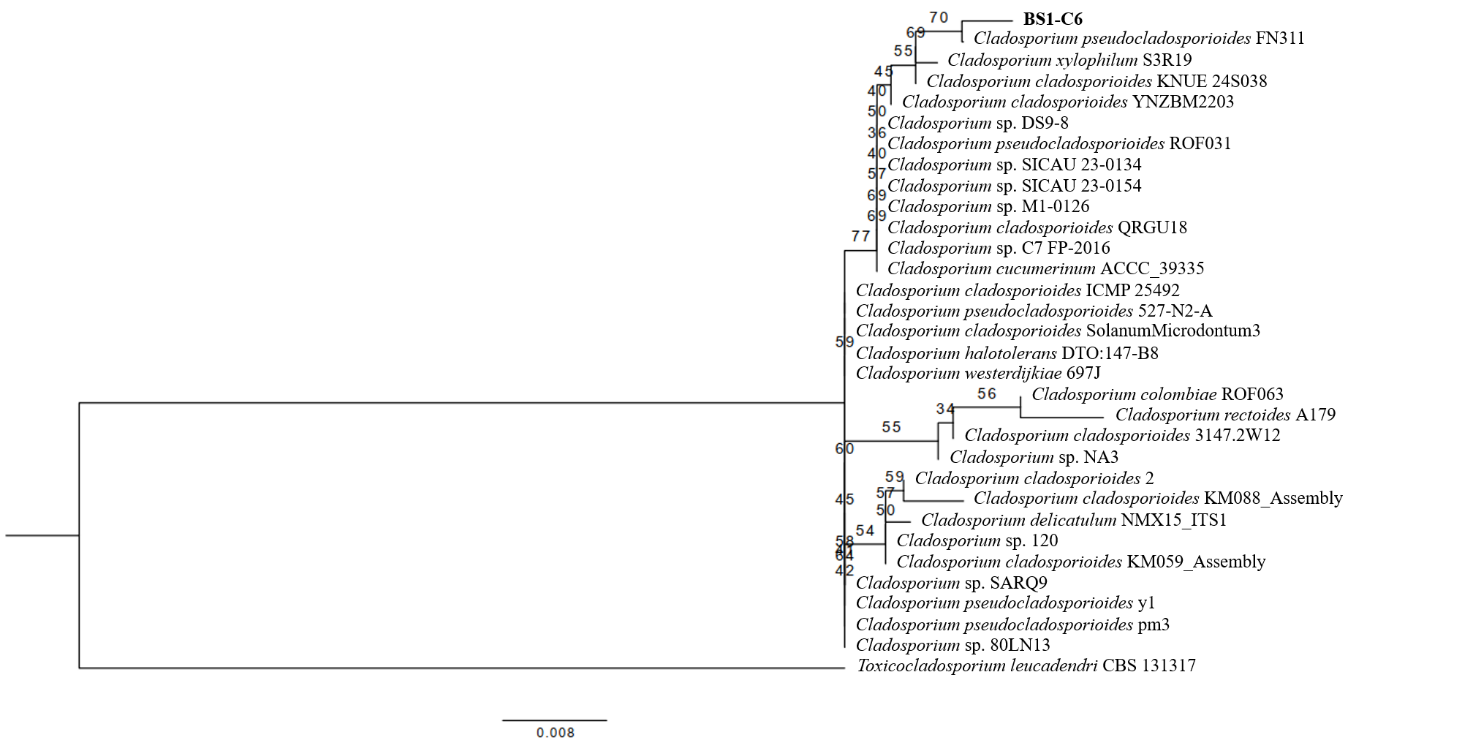


Isolate BS2-C3

Substitution model: HKY+I

Outgroup species: *Metarhizium acridum*


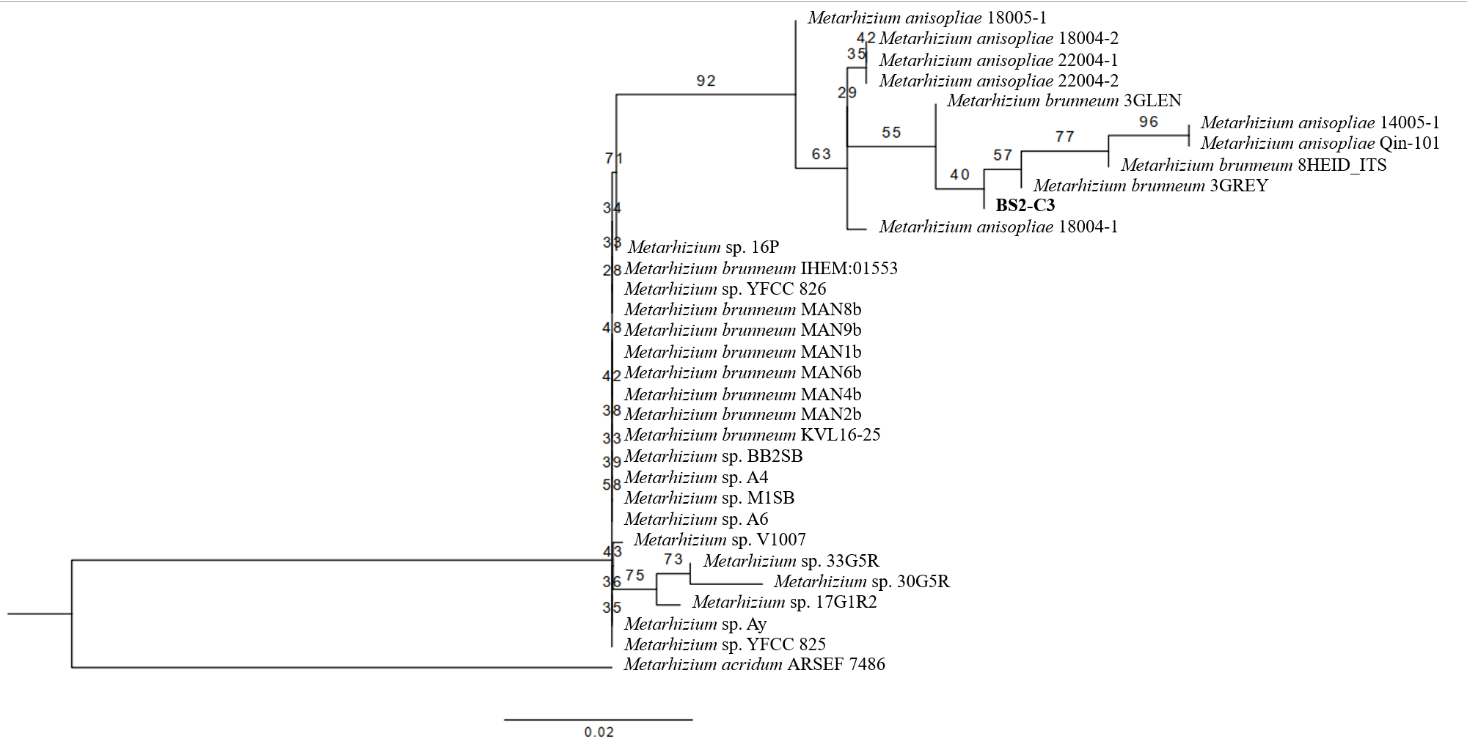


Isolate BS2-C4

Substitution model: JC+G4

Outgroup species: *Calonectria naviculata*


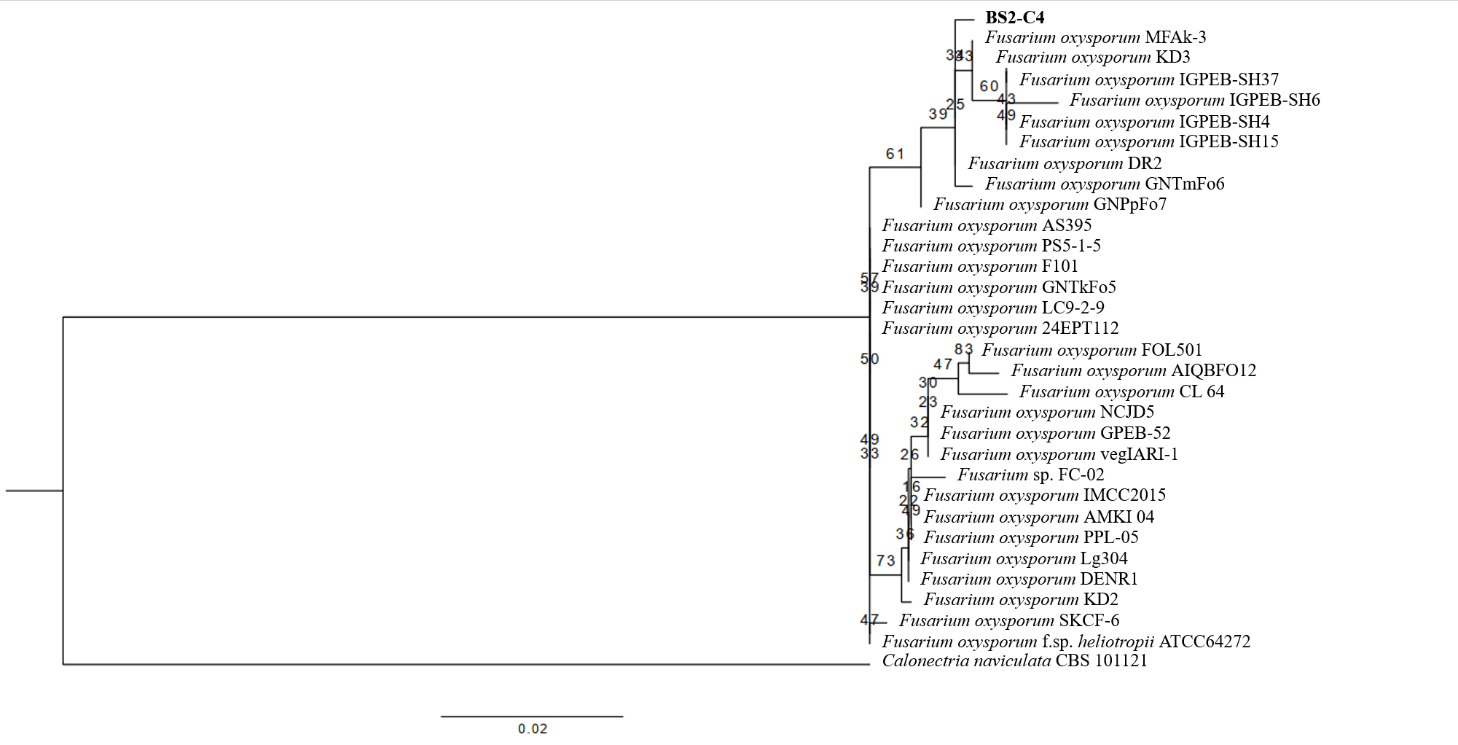

Supplement: tjag069_Supplementary_Data [file tjag069_supplementary_data.zip › Supplementary Material S3.docx]
