# Supplementary material for: Screening of environmental fungi from Crete reveals candidates for biological control of mosquitoes
Source: J Med Entomol. 2026 May 26;63(3):tjag069. doi: 10.1093/jme/tjag069 (PMC13202464; doi:10.1093/jme/tjag069)
Supplement: tjag069_Supplementary_Data [file tjag069_supplementary_data.zip › Supplementary Material S1.docx]

**Supplemental Material S1.** Sampling sites with their corresponding GPS coordinates. Site selection was based on habitat type (riverside, forest, grassland, and agricultural areas), vegetation composition (olive groves, grass patches, shrubs, pines and oak trees, herbaceous plants), soil characteristics (moisture, organic matter), and insect presence.

| Sampling site number | Geographic coordinates |
| --- | --- |
| 1 | 35.30608° N, 25.10164° E |
| 2 | 35.38224° N, 24.69957° E |
| 3 | 35.15358° N, 24.47312° E |
| 4 | 35.31125° N, 24.30060° E |
| 5 | 35.21907° N, 25.44639° E |
| 6 | 35.25593° N, 25.38465° E |
| 7 | 35.04567° N, 25.39884° E |
| 8 | 35.16457° N, 24.99958° E |
| 9 | 35.12839° N, 24.94576° E |
| 10 | 35.14410° N, 24.89824° E |
| 11 | 35.39407° N, 24.13104° E |
| 12 | 35.42372° N, 24.21851° E |
| 13 | 35.45193° N, 23.92086° E |
| 14 | 35.43757° N, 24.06841° E |
| 15 | 35.22799° N, 25.36628° E |
| 16 | 35.21045° N, 25.30650° E |
| 17 | 35.33042° N, 24.67745° E |


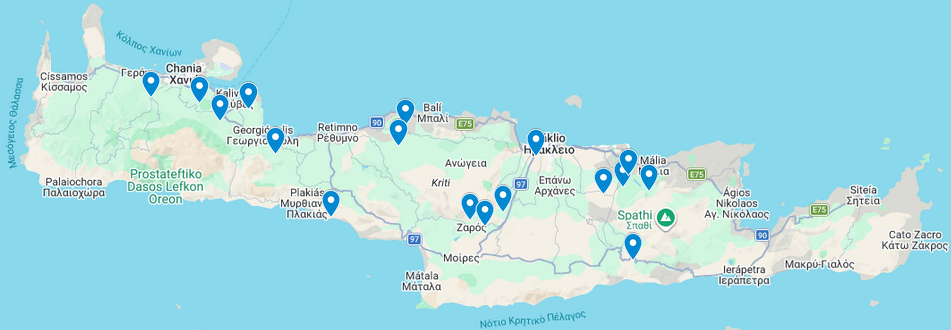


Map sourced from Google Maps (n.d.): Map of Crete with locations of sample collection sites plotted as destinations. Retrieved from:

<https://www.google.com/maps/d/u/0/viewer?mid=11oabtorRJaZPHspqt09HTk8GRnqxvyg&ll=35.30824180491517%2C24.687733182732465&z=13> on 27 March 2026 using Google Map Data© 2026.
